# Diverse biofilm-forming Sphingomonadaceae represent twelve novel species isolated from glaciers on the Tibetan Plateau

**DOI:** 10.1099/ijsem.0.006913

**Published:** 2025-09-08

**Authors:** Dou Han, Yu-Hua Xin, Qing Liu

**Affiliations:** 1 State Key Laboratory of Microbial Diversity and Innovative Utilization, Institute of Microbiology, Chinese Academy of Sciences, Beijing 100101, PR China; 2 College of Biological Sciences, China Agricultural University, Beijing 100093, PR China; 3 China General Microbiological Culture Collection Center (CGMCC), Institute of Microbiology, Chinese Academy of Sciences, Beijing 100101, PR China; 4 Beijing Key Laboratory of Genetic Element Biosourcing & Intelligent Design for Biomanufacturing, Beijing 100101, PR China

**Keywords:** aerobic anoxygenic phototrophic bacteria, biofilm, cold adaptation, glacier-inhabiting bacteria, *Sphingomonas*

## Abstract

The family *Sphingomonadaceae*, encompassing the genus *Sphingomonas* and related taxa, comprises diverse Gram-negative, aerobic, rod-shaped bacteria found in varied habitats, including air, soil, water and glaciers. Recent genomic-based taxonomic revisions have reclassified some *Sphingomonas* species into new genera, such as *Parasphingomonas* and *Alteristakelama*, due to polyphyletic relationships within the family *Sphingomonadaceae*. Certain *Sphingomonadaceae* species are known for forming biofilms or functioning as aerobic anoxygenic phototrophic bacteria, traits that enhance resilience in extreme environments like the cryosphere. In this study, we isolated 12 novel strains from Tibetan Plateau glaciers, revealing significant phenotypic and genotypic diversity. Based on phylogenomic analyses, six strains were classified within *Sphingomonas*, five within *Parasphingomonas* and one within *Alteristakelama*. These strains exhibit broad pH (4–11), salt tolerance (0–3.0%) and temperature adaptability (0–37 °C), alongside varied metabolic capabilities, including diverse carbon source utilization and enzyme activities. Eleven strains exhibit biofilm formation, and some possess genes for carotenoid biosynthesis and photosynthesis. The 16S rRNA gene sequence similarities with their closest relatives ranged from 97.6% to 99.9%, while the average nt identity and digital DNA–DNA hybridization values between these strains and known species with validly published names were below 89.80% and 36.60%, respectively. Polyphasic analyses, encompassing phylogenetic, phenotypic and genotypic analyses, confirm that these strains represent 12 novel species within the family *Sphingomonadaceae*. We propose the following names: *Alteristakelama amylovorans* sp. nov., *Sphingomonas sorbitolis* sp. nov., *Sphingomonas fucosidasi* sp. nov., *Sphingomonas sandaracina* sp. nov., *Sphingomonas rhamnosi* sp. nov., *Sphingomonas arabinosi* sp. nov., *Sphingomonas flavida* sp. nov., *Parasphingomonas frigoris* sp. nov., *Parasphingomonas halimpatiens* sp. nov., *Parasphingomonas zepuensis* sp. nov., *Parasphingomonas caseinilytica* sp. nov. and *Parasphingomonas puruogangriensis* sp. nov. This study enhances our understanding of *Sphingomonadales* diversity and its ecological adaptations in extreme environments.

## Introduction

The genus *Sphingomonas* was first proposed by Yabuuchi *et al*. [1] with *Sphingomonas paucimobilis* as the type species, and its description has been emended several times [2,7]. It encompasses Gram-negative, strictly aerobic, orange-, yellow- or off-white-pigmented, rod-shaped and sphingolipid-containing bacteria [2,5]. At the time of writing this manuscript (July 2025), the genus *Sphingomonas* comprised 171 species with validly published names [8], highlighting its diversity. However, recent genomic-based taxonomic studies have revealed polyphyly within the family *Sphingomonadaceae*, leading to significant reclassification. Wang *et al*. [9] restructured the order *Sphingomonadales* into 13 families, including 9 novel ones, and reassigned 163 species into new genera, such as *Flavisphingomonas*, *Parasphingomonas* and *Alteristakelama*, based on phylogenomic analyses, average aa identity and evolutionary distance thresholds. This reclassification clarifies the taxonomic framework of *Sphingomonas* and related genera, enhancing insights into their ecological and biotechnological roles.

Some bacteria can adapt to their environment by producing extracellular polymeric substances, which form a protective matrix encapsulating biofilm communities [10]. Members of *Sphingomonas* and related genera are notable for their ability to form biofilms, attracting widespread research interest. These bacteria are widely distributed and have been isolated from various habitats, such as air [2], lakes [11], soil [12,13], plant roots [14] and glaciers [15]. The biofilm-forming characteristics of *Sphingomonas* and related genera may result in metal corrosion in pipeline systems, as well as biological contamination in drinking water and industrial water distribution systems [16,17]. De Vries *et al*. [18] found that biofilm-forming strains within *Sphingomonas* exhibit high tolerance to varying pH levels, salt concentrations and temperatures.

*Sphingomonadaceae* has also been found to be dominant in the cryosphere, such as in the ice cores from the Tibetan Plateau [19]. It was also one of the major bacterial groups in the Arctic and Antarctica [20,22]. Biofilm formation is considered one of the survival strategies for bacteria in cold environments. Smith *et al*. [23] found that microbial communities on the surface of Antarctic glaciers exist in biofilm form, covering ~35% of the ice melt surface. However, it remains unclear whether the biofilm-forming ability of this group is related to its ecological distribution in the cryosphere. Additionally, carotenoid production has been suggested to play a role in the adaptation of bacteria living on glacier surfaces, where they are exposed to intense light irradiation [24]. As a group of aerobic anoxygenic phototrophic bacteria [25], *Sphingomonadaceae* in glacial environments may enhance the efficiency of organic carbon utilization. Additionally, its ability to harness light energy could provide a survival advantage over other types of bacteria.

During a survey of bacterial diversity on the surface of glaciers located on the Tibetan Plateau, P.R. China, we isolated 12 cold-adapted *Sphingomonadaceae* strains. Based on the revised taxonomy by Wang *et al*. [9], 6 strains were classified within *Sphingomonas*, 5 within *Parasphingomonas* and 1 within *Alteristakelama*, with 11 exhibiting biofilm-forming capabilities. This study is of significant importance for understanding the diversity, phenotypic characteristics and adaptation mechanisms of *Sphingomonas* and related genera in glacial surface environments.

## Methods

### Isolation and culture conditions

Between 11 and 16 October 2016, ice and cryoconite samples were collected from ~20 cm in depth across various locations on the Tibetan Plateau, P.R. China. The sampling sites included the Laigu Glacier (29° 18′ 32″ N 96° 49′ 7″ E), Renlongba Glacier (29° 15′ 42″ N 96° 56′ 9″ E), Zepu Glacier (30° 16′ 36″ N 95° 15′ 3″ E), Gawalong Glacier (29° 45′ 57″ N 95° 42′ 37″ E) and Puruogangri Glacier (33° 55′ 6″ N 89° 2′ 17″ E). Throughout the sampling process, sterile gloves were worn to ensure contamination prevention. The samples were carefully excavated using a sterile axe, placed into sterile sampling bags and then transported to the laboratory in an incubator packed with dry ice. The samples were diluted in series with sterile water and then spread onto Reasoner’s 2A agar (BD Difco) with fourfold dilution and onto peptone, yeast extract and glucose (PYG) medium [15]. After incubation at 14 °C, more than 2,000 bacterial isolates were collected. In this study, 12 strains listed in Table 1 were obtained and purified by repeated streaking on PYG agar. These strains were routinely incubated at 20 °C and preserved in aqueous glycerol suspensions (10%, v/v) in a liquid nitrogen storage tank.

**Table 1.** Twelve novel species proposed in this study

Strain	Proposal name	CGMCC no.	KACC no.	Isolation source	Genome accession no.
LT1P40^T^	*Alteristakelama amylovorans*	1.11403	23651	Cryoconite	JAXOJT000000000
LB2R24^T^	*Sphingomonas sorbitolis*	1.11562	23650	Ice	JAXOJS000000000
LB3N6^T^	*Sphingomonas fucosidasi*	1.11646	23649	Ice	JAXOJR000000000
ZB1N12^T^	*Sphingomonas arabinosi*	1.23968	23645	Ice	JAXOJN000000000
PB2P12^T^	*Sphingomonas sandaracina*	1.25174	23642	Ice	JAXOJK000000000
PB2P19^T^	*Sphingomonas rhamnosi*	1.25194	23641	Ice	JAXOJJ000000000
PB1R3^T^	*Sphingomonas flavida*	1.25232	23639	Ice	JAXOJH000000000
RB3P16^T^	*Parasphingomonas frigoris*	1.11860	23648	Ice	JAXOJQ000000000
RT2P30^T^	*Parasphingomonas halimpatiens*	1.23559	23647	Cryoconite	JAXOJP000000000
ZT3P38^T^	*Parasphingomonas zepuensis*	1.23914	23646	Cryoconite	JAXOJO000000000
GB1N7^T^	*Parasphingomonas caseinilytica*	1.24759	23643	Ice	JAXOJL000000000
PB4P5^T^	*Parasphingomonas puruogangriensis*	1.25204	23640	Ice	JAXOJI000000000

### 16S rRNA sequencing and phylogeny

The genomic DNA of the 12 strains was extracted using the TaKaRa MiniBEST Bacteria Genomic DNA Extraction Kit v3.0 (TaKaRa), following the manufacturer’s instructions. The 16S rRNA gene sequences of these strains were then amplified and sequenced using the bacterial universal primers 27F and 1492R [26]. The PCR was performed with an initial denaturation step of 4 min at 94 °C, followed by 30 cycles of 1 min at 94 °C, 1 min at 55 °C and 1 min at 72 °C each, followed by a final extension of 10 min at 72 °C. By comparing the obtained 16S rRNA gene sequences with those available on the EzBioCloud server [27], the closest phylogenetic neighbour for each strain was identified. Multiple sequence alignments were conducted using the MAFFT software v7.520 with default parameters [28]. The phylogenetic tree was constructed using the neighbour-joining algorithm with Kimura’s two-parameter model in the mega software package v5.2 [29]. The tree topologies were evaluated by bootstrap values based on 1,000 resamplings.

### Genome features and phylogenomic analysis

The whole genomes of the 12 novel isolates, as well as *Sphingomonas oligophenolica* CGMCC 1.10181^T^ and *Sphingomonas qilianensis* CGMCC 1.15349^T^, were sequenced using an Illumina HiSeq 4000 platform (Illumina, San Diego, CA, USA), generating 150 bp paired-end reads following the manufacturer’s protocols. The short reads were *de novo* assembled using SPAdes v3.14 [30]. The CheckM2 v1.0.2 program was used to check the completeness and contamination values of the genomes [31]. The quality of the assemblies was assessed using QUAST v5.0.2 [32]. Gene prediction and annotation were performed using the Prokka software v1.14 [33]. A total of 145 genomic sequences of type strains of *Sphingomonas* and related genera were downloaded from GenBank for comparative analysis. The pairwise average nt identity (ANI) values between the analysed strains were calculated using the FastANI program [34]. Clustering of the pairwise ANI matrix was performed using the ‘bactaxR’ package in R with the average linkage hierarchical clustering method [35]. The digital DNA–DNA hybridization (dDDH) value was determined using the TYGS as implemented on the DSMZ website [36]. Ninety-two core genes were identified from the genomic sequences using the UBCG program [37]. Multiple sequence alignments of the concatenated core gene sequences were conducted using the MAFFT software v7.520, and the gaps were removed using the Gblocks program [38]. The maximum-likelihood phylogenomic tree was constructed using IQ-TREE software [39] with 1,000 bootstrap replicates, employing the best model of GTR+F+R5. The Kyoto The CAZy database v11 was used for carbohydrate-active enzyme (CAZyme) identification [40]. The correlation between the carbon source utilization capacity and the number of CAZymes was evaluated using the Pearson correlation coefficient, with the corresponding *P*-value calculated to assess the significance of the correlation.

### Physiology and chemotaxonomy

The morphology of colonies was assessed following cultivation on PYG agar for 4 days. Cellular characteristics were examined using transmission electron microscopy with an HT7800 TEM/Regulus 8100 transmission electron microscope (Hitachi Ltd., Tokyo, Japan). Motility was observed by oil-immersion phase-contrast microscopy after incubation on PYG agar. Growth was evaluated across pH levels (ranging from pH 4.0 to 11.0 at intervals of 1 pH unit) and various NaCl concentrations (0–3.5% w/v at 0.5% intervals) in PYG broth at 20 °C for 8 days. Appropriate biological buffers (0.2 M Na_2_HPO_4_/NaH_2_PO_4_ for pH 4–8 and 0.2 M Na_2_CO_3_/NaHCO_3_ for pH 9–10) were used to adjust the PYG broth. Starch, casein and Tween 80 hydrolysis was performed following the method outlined by Smibert and Krieg [41]. Carbon source utilization was tested using the GEN III MicroStation (Biolog) or an API 50CH strip (bioMérieux, Marcy-l’Étoile, France). Enzyme activities and biochemical tests were conducted using the API 20E, 20NE and ZYM strips (bioMérieux) according to the manufacturer’s protocols. For cellular fatty acid composition analysis, cells were harvested from colonies on PYG agar plates after incubation at 20 °C. The fatty acids were extracted following the MIDI 6.0 protocol [42] and identified using the Agilent 6890 N GC system (Agilent Technologies, Santa Clara, CA, USA) with the TSBA6 database.

### Detection of biofilm-forming ability

Biofilm formation of the 12 strains was assayed using a modified crystal violet staining method under static conditions [43]. A non-inoculated medium served as the negative control, while *Pseudomonas fluorescens* CGMCC 1.1802^T^ was used as the positive control. The strains were cultured overnight and then diluted in fresh PYG medium to an OD_600_ of 0.1. The diluted bacterial solution was inoculated into a 96-well plate with 200 µl per well, in triplicate. The inoculated bacterial solution was then incubated at 20 °C for 2 weeks. After incubation, the free and loosely attached bacteria were aspirated, and the wells were gently washed three times with normal saline. Subsequently, 120 µl of 0.1% crystal violet was added to stain the firmly attached bacterial cells in the wells for 30 min at room temperature. After staining, the crystal violet solution was aspirated, and the unattached crystal violet was washed away with normal saline. The crystal violet adhered to the 96-well plate was dissolved in 220 µl of 30% acetic acid, and the OD of the solution at 590 nm was measured using a multi-label microplate detection system (BMG LABTECH, DEU).

## Results and discussion

### 16S rRNA gene sequence phylogenetic analysis

The 16S rRNA gene sequence similarities of the 12 strains with their closest neighbours ranged from 97.6% to 99.9% (Table 2), indicating that these strains belong to the genera *Alteristakelama*, *Parasphingomonas* and *Sphingomonas*. A phylogenetic analysis based on 16S rRNA gene sequences of all 12 strains and their relatives in the genus *Sphingomonas* and related genera was reconstructed, showing each to be distributed across multiple clades of the phylogenetic tree (Fig. S1, available in the online Supplementary Material). Despite the high 16S rRNA gene sequence similarities between glacier-inhabiting strains and non-glacial isolate phylogenetic neighbours, most of these strains formed independent, well-separated branches in the tree, such as strain LT1P40^T^. However, strains LB3N6^T^ and *Sphingomonas faeni* MA-olki^T^ (99.9%), as well as strains RB3P16^T^ and *Parasphingomonas glacialis* C16y^T^ (99.6%), formed small branches. These results indicate that *Sphingomonadaceae* exhibits phylogenetic diversity in glacial environments.

**Table 2.** The 16S rRNA gene sequence similarity, ANI and dDDH values between the 12 isolates and their closest relatives Length of successfully aligned nt was produced for comparison of 16S identity.

Strain	Nearest phylogenetic neighbour	16S rRNA gene sequence similarity (%)	Length of successfully aligned nucleotides (nt)	ANI (%)	dDDH (%)
LT1P40^T^	*Alteristakelama koreensis* NBRC 16723^T^	97.56	1,317	81.75	22.30
LB2R24^T^	*S. faeni* MA-olki^T^	99.11	1,339	89.11	35.40
	LB3N6^T^	99.63	1,348	89.80	36.60
LB3N6^T^	*S. faeni* MA-olki^T^	99.93	1,350	88.16	33.90
RB3P16^T^	*P. glacialis* C16y^T^	99.56	1,347	88.32	32.40
	*Parasphingomonas psychrolutea* MDB1-A^T^	97.86	1,324	82.99	23.60
RT2P30^T^	*S. oligophenolica* JCM 12082^T^	98.75	1,267	81.11	21.70
	*Parasphingomonas echinoides* ATCC 14820^T^	98.24	1,340	80.42	20.20
ZT3P38^T^	*S. oligophenolica* JCM 12082^T^	99.29	1,256	82.80	23.80
ZB1N12^T^	*S. faeni* MA-olki^T^	99.4	1,321	89.42	35.80
	*Sphingomonas aurantiaca* MA101b^T^	99.25	1,318	87.31	31.70
GB1N7^T^	*Parasphingomonas aliaeris* DH-S5^T^	99.16	1,298	83.73	25.20
PB2P12^T^	*S. faeni* MA-olki^T^	99.54	1,298	87.77	32.30
PB2P19^T^	*Sphingomonas aerolata* NW12^T^	98.79	1,302	83.64	24.20
	*Sphingomonas ginsenosidivorax* KHI67^T^	98.04	1,298	87.17	29.70
PB4P5^T^	*Parasphingomonas qilianensis* CGMCC 1.15349^T^	99.62	1,327	85.65	29.10
	*Parasphingomonas hylomeconis* GZJT-2^T^	99.47	1,325	86.71	30.20
PB1R3^T^	*Sphingomonas sanguinis* NBRC 13937^T^	99.24	1,308	88.14	32.30

### Genome features and phylogenomic analysis

To further determine their taxonomic status, the genomes of these strains, as well as strains *S. oligophenolica* CGMCC 1.10181^T^ and *S. qilianensis* CGMCC 1.15349^T^, were sequenced. The completeness of all the genome sequences was above 99.5%, with contamination lower than 2.5%. The basic information of the sequenced genomes is listed in Table S1. Except for strains RB3P16^T^ and ZT3P38^T^, fewer than 100 contigs were assembled from the raw sequenced data of these strains. The genomic sizes of these strains ranged from 3.42 to 6.26 Mb and the G+C content was 64.4–66.2 mol%. Notably, the genome size of strain *S. oligophenolica* CGMCC 1.10181^T^ (6.26 Mb) was much larger than that of other species.

**Fig. 1.**
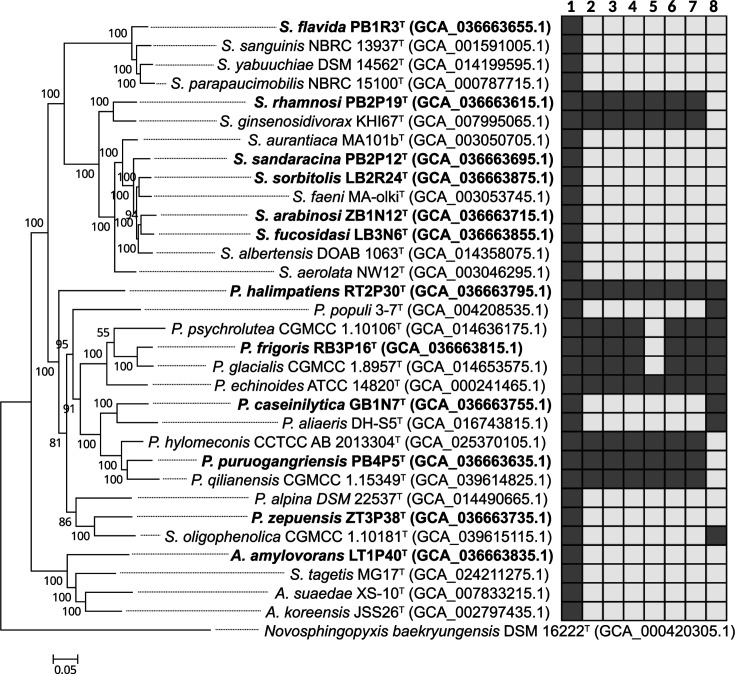
Phylogenomic tree, constructed based on 92 core genes, delineates the evolutionary relationships among the 12 novel strains and their closely related type strains within the genera *Alteristakelama*, *Parasphingomonas* and *Sphingomonas*. Genomic sequence accession numbers are given in parentheses. *Novosphingopyxis baekryungensis* DSM 16222^T^ was used as an outgroup. Numbers 1–8 represent the genes *crtBIY*, *crtCDF*, *pufL*, *pufM*, *pufB*, *pufC*, *bchIDXYZMLBN* and *bchE*. Black and white squares represent the presence or absence of these genes, respectively. Bootstrap values (>50%) based on 1,000 replicates are shown at the branch nodes. Bar, 0.05 nt substitutions per site.

Ninety-two core genes were extracted from genomic sequences of *Sphingomonas* and related genera for phylogenomic tree construction using the maximum-likelihood method (Figs 1 and S2). Strains LT1P40^T^ clustered into the branch of the genus *Alteristakelama*, while strains GB1N7^T^, PB4P5^T^, RB3P16^T^, ZT3P38^T^ and RT2P30^T^ grouped into the branch of the genus *Parasphingomonas*, indicating that they could be classified into the new genera *Alteristakelama* and *Parasphingomonas*, respectively, which were described by Wang *et al*. [9]. The other six strains were classified into the genus *Sphingomonas* with 100% bootstrap support (Fig. S2), indicating that they belong to the genus *Sphingomonas*. A smaller tree, including the 12 novel strains and their close relatives, was reconstructed to illustrate their phylogenetic relationships, showing that they were well separated from their close relatives, thus suggesting their potential as novel species (Fig. 1).

The pairwise ANI and dDDH values between these strains and the 145 species of the genus *Sphingomonas* and related genera with validly published names were calculated (Table S2). The highest ANI and dDDH values between the 12 novel strains and their closest relatives ranged from 80.4% to 89.8% and 20.2% to 36.6%, respectively, as listed in Tables 2 and S2. The pairwise ANI and dDDH values among the known species were also calculated. The highest values were found between *S. parapaucimobilis* NBRC 15100^T^ and *Sphingomonas yabuuchiae* DSM 14562^T^ (ANI: 91.8%, dDDH: 44.1%), followed by *Sphingomonas sanguinis* NBRC 13937^T^ and *S. yabuuchiae* DSM 14562^T^ (ANI: 89.2%, dDDH: 35.1%) and *Sphingomonas albertensis* DOAB 1063^T^ and *S. faeni* MA-olki^T^ (ANI: 88.2%, dDDH: 34.2%). All these values were below the suggested threshold (95–96% for ANI and 70% for dDDH value) for delineating bacterial species [44,45]. Cluster analysis of the ANI values also indicated that these 12 strains do not belong to any known species (Fig. S3). Based on these findings, we concluded that they represent 12 novel species belonging to the genera *Sphingomonas*, *Alteristakelama* and *Parasphingomonas*. The proposed names are listed in Table 1. We compared the 12 strains against 1,262 *Sphingomonas* genomes from the NCBI using ANI calculations. As detailed in Table S3, only strains LB2R24^T^, LB3N6^T^ and PB1R3^T^ exhibited ANI values >95 % with other genomes, indicating multiple representative genomes for these species. The remaining nine potential novel species currently each have a single representative strain.

### Phenotypic characteristics

The phenotypic and chemotaxonomic characteristics of 12 novel species were evaluated (Tables S3 and S4). These strains formed yellow or orange colonies and were identified as Gram-stain-negative, rod-shaped bacteria that tested positive for both catalase and oxidase. They exhibited a wide range of pH tolerance, with the ability to withstand acidic conditions of pH 4.0 or 5.0. However, their tolerance to alkaline conditions varied, with some strains growing only up to a pH of 8.0, while others could grow at pH 11.0. All strains could grow at 0% NaCl concentration, although the maximum NaCl tolerance concentration varied widely from 0.05% to 3.0%, showing similar characteristics to those of known species (Table S4).

These 12 strains exhibit both similarities and differences in growth temperature when compared with known species. Many of the new strains share low-temperature growth ranges with species such as *Parasphingomonas psychrolutea* (0–25 °C) and *P. glacialis* (1–30 °C), suggesting their adaptation to cold environments. However, some strains, like PB1R3^T^, can grow at higher temperatures up to 37 °C, similar to *S. oligophenolica* and *Sphingomonas ginsenosidivorax*, which is uncommon among these related species. Additionally, strains such as RT2P30^T^ and ZT3P38^T^ can grow at 35 °C, while others like LB2R24^T^ and LB3N6^T^ show a more limited range, with growth restricted to a maximum of 25 °C. Overall, these new strains demonstrate both low-temperature adaptability and diverse temperature tolerance, with some showing the ability to grow at higher temperatures (Table S4). The carbon source utilization, enzyme activities and other biochemical characteristics further highlight the phenotypic diversity of these novel strains (Table S5). The detailed phenotypic features for each strain can be found in the species descriptions and in Table S5.

**Fig. 2.**
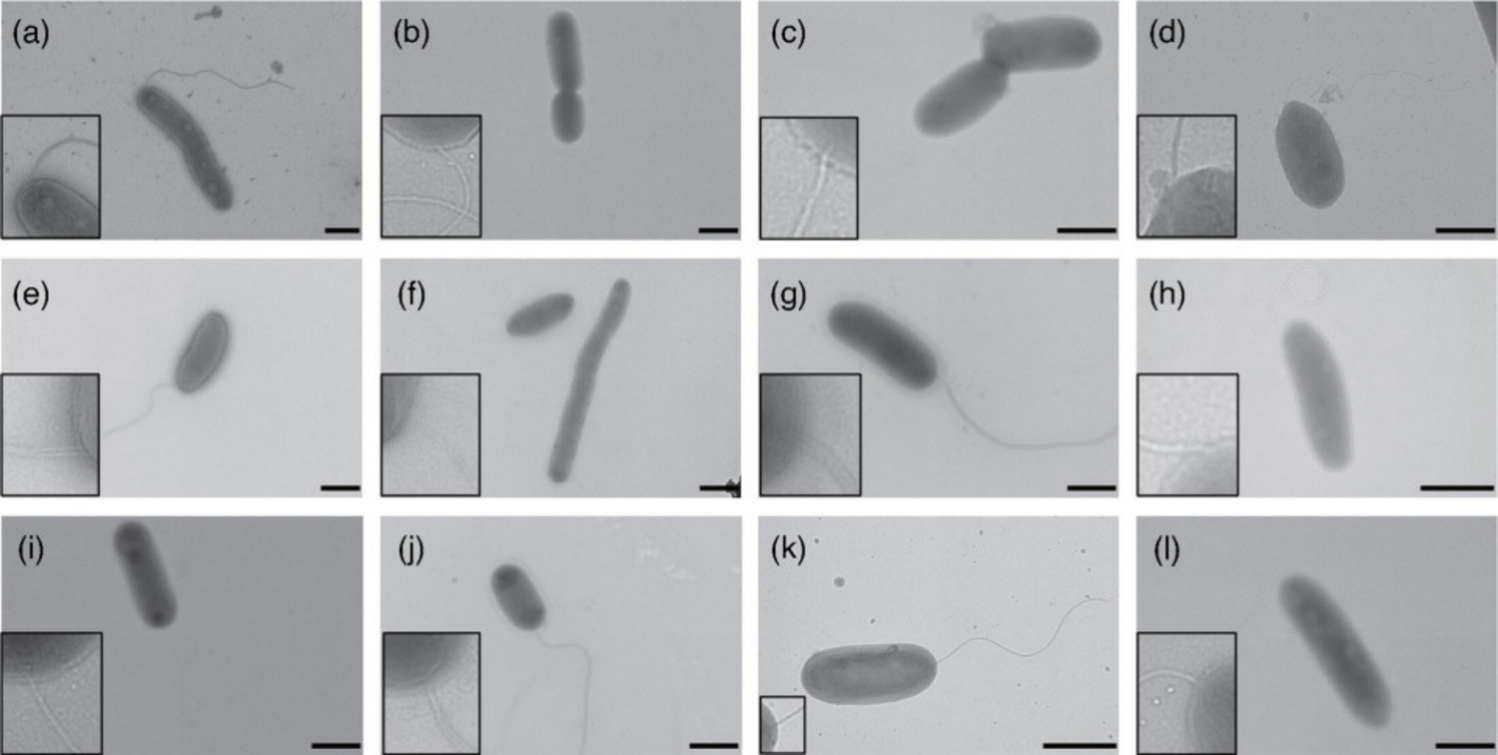
Transmission electron micrograph of negatively stained cells of the type strains of 12 novel species. Letters (a)–(l) represent LT1P40^T^, LB2R24^T^, LB3N6^T^, RB3P16^T^, RT2P30^T^, ZT3P38^T^, ZB1N12^T^, GB1N7^T^, PB2P12^T^, PB2P19^T^, PB4P5^T^ and PB1R3^T^, respectively. The black line represents a scale of 1 µm. The inset panels in the lower left corner display an enlarged view of the flagellum.

**Fig. 3.**
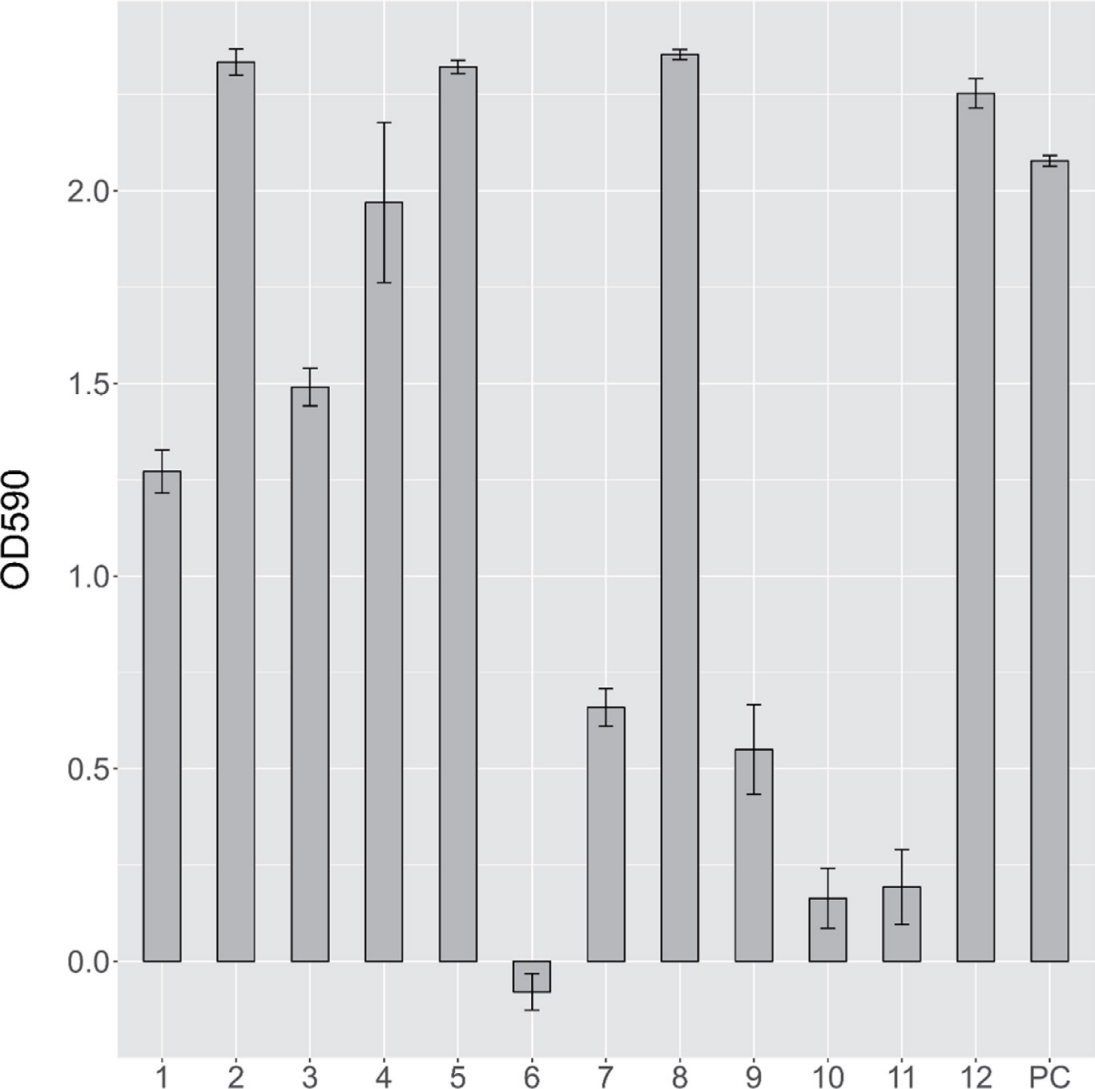
Biofilm formation of the 12 strains: OD590 values of the crystal violet as a measurement of attached biomass. Numbers 1–12 represent LT1P40^T^, LB2R24^T^, LB3N6^T^, RB3P16^T^, RT2P30^T^, ZT3P38^T^, ZB1N12^T^, GB1N7^T^, PB2P12^T^, PB2P19^T^, PB4P5^T^ and PB1R3^T^, respectively. Strain *Pseudomonas fluorescens* CGMCC 1.1802^T^ was used as a positive control (PC). The final results were obtained by subtracting the OD of the negative control group from that of the experimental (inoculated) group.

The fatty acid composition of the 12 novel strains is listed in Table 3. All strains contained summed feature 8 (C_18 : 1_* ω*7c/C_18 : 1_* ω*6c) as the major fatty acid. Other unsaturated fatty acids, such as summed feature 3 (C_16 : 1_* ω7*c/_C16 : 1_* ω*6c) or C_17 : 1_* ω*6c, were found at abundant levels in certain strains. Cells of the 12 strains possessed a single flagellum, indicating swimming motility (Fig. 2). In the oligotrophic environment of glaciers, motility likely plays a crucial role in both resource acquisition and dispersal. Additionally, the biofilm formation capabilities of the 12 strains were assessed under static conditions using a 96-well plate assay. The final results were obtained by subtracting the OD of the negative control group from that of the experimental (inoculated) group. All strains, except ZT3P38^T^, formed biofilms (Fig. 3), with varying amounts of attached biomass. Strains LB2R24^T^, RT2P30^T^, GB1N7^T^ and PB1R3^T^ exhibited the strongest biofilm formation ability, with OD590 values significantly higher than those of the remaining strains and the positive control (*P*<0.005).

**Table 3.** Cellular fatty acid composition (per cent) of the type strains of 12 novel species Numbers 1–12 represent LT1P40^T^, LB2R24^T^, LB3N6^T^, RB3P16^T^, RT2P30^T^, ZT3P38^T^, ZB1N12^T^, GB1N7^T^, PB2P12^T^, PB2P19^T^, PB4P5^T^ and PB1R3^T^, respectively. Values are percentages of the total fatty acids. tr, traces (<1% of the total fatty acids); –, not detected.

Fatty acid	1	2	3	4	5	6	7	8	9	10	11	12
**Saturated**												
C_14 : 0_	tr	1.7	1.6	2.0	tr	tr	tr	tr	1.7	1.1	tr	1.2
C_16 : 0_	9.7	7.0	10.2	12.2	13.8	14.8	10.2	18.2	8.6	8.1	14.0	6.2
C_17 : 0_	1.7	tr	–	tr	tr	tr	–	tr	–	tr	–	tr
C_18 : 0_	tr	tr	tr	1.2	1.3	tr	tr	tr	tr	tr	tr	1.5
**Unsaturated**												
C_16 : 1_* ω*5c	tr	3.1	3.7	1.2	10.7	tr	1.7	1.0	2.5	2.5	1.6	tr
C_17 : 1_* ω*6c	27.7	tr	tr	tr	2.9	tr	tr	1.8	tr	tr	tr	tr
C_17 : 1_* ω*8c	3.5	tr	tr	–	tr	–	–	tr	–	tr	–	tr
C_18 : 1_* ω*5c	tr	1.5	1.7	1.4	1.6	1.0	1.6	tr	1.2	tr	tr	2.3
**Branched**												
Iso-C_16 : 0_ 3-OH	tr	tr	–	tr	tr	tr	1.1	tr	1.5	tr	tr	tr
C_18 : 1_* ω*7c 11-methyl	2.1	3.8	6.8	4.5	2.5	6.6	5.8	8.9	5.7	3.1	4.9	–
**Hydroxy fatty acids**												
C_14 : 0_ 2-OH	4.9	7.1	7.2	3.9	11.0	11.6	11.3	10.6	6.0	8.4	8.1	5.9
C_15 : 0_ 2-OH	6.6	–	tr	–	1.1	tr	–	tr	–	tr	–	tr
C_16 : 1_ 2-OH	–	1.1	tr	tr	–	tr	1.5	tr	tr	3.0	tr	–
C_18 : 1_ 2-OH	–	tr	tr	tr	–	tr	1.7	tr	1.7	tr	–	tr
Summed feature*												
3	9.9	23.1	20.0	6.4	1.8	2.9	14.5	4.7	23.6	20.0	16.6	3.6
8	30.7	46.4	45.0	65.6	51.6	60.1	47.4	49.9	45.4	48.4	50.7	77.4

*Summed features represent groups of two fatty acids that could not be separated by GC with the MIDI system to the following standard text: Summed features are fatty acids that cannot be resolved reliably from another fatty acid using the chromatographic conditions chosen. The MIDI system groups these fatty acids together as one feature with a single percentage of the total. Summed features consist of the following: 3, C_16 : 1_* ω7*c/_C16 : 1_* ω*6*c*, and 8, C_18 : 1_* ω7*c/_C18 : 1_* *ω*6c.*

### CAZyme annotation

Genes encoding CAZymes were annotated using the CAZy database, with the number of genes ranging from 98 to 163 per strain. Glycoside hydrolases (GHs) were the most abundant (48–93 genes). The number of GHs correlated significantly with the number of utilized carbon sources (Fig. S4). CAZymes in the GH family are involved in carbohydrate degradation [46], suggesting a causal relationship between carbon source utilization capacity and the number of GH-encoding genes. The ability to metabolize carbohydrates may be gained or lost through horizontal gene transfer [47].

### Detection of carotenoid biosynthesis and photosynthesis gene cluster

The genes *crtB*, *crtI* and *crtY* encoding phytoene synthase, phytoene desaturase and lycopene *β*-cyclase were present in all 12 isolates and type strains of related species, suggesting that *β*-carotene may be the major pigment. The discovery of carotenoid genes in these strains suggests that the yellow or orange colony colours observed are likely due to the carotenoids they produce. Four strains (RB3P16^T^, RT2P30^T^, PB2P19^T^ and PB4P5^T^) contained *crtC*, *crtD* and *crtF* genes, which are involved in spirilloxanthin synthesis. These four strains also contained photosynthesis gene clusters (PGCs), with *puf* genes encoding the reaction centre proteins and *bch* genes involved in BChl*a* biosynthesis. Strains RT2P30^T^, PB2P19^T^ and PB4P5^T^ contained *pufL*, *pufM*, *pufB* and *pufC*, while strain RB3P16^T^ contained *pufL*, *pufM* and *pufC* but not *pufB*. The *bchIDXYZMLBNE* genes were present in strains RB3P16^T^ and RT2P30^T^, whereas PB2P19^T^ and PB4P5^T^ contained *bchIDXYZMLBN* genes. The PGCs were also present in *S. ginsenosidivorax* KHI67^T^, *P. psychrolutea* CGMCC 1.10106^T^, *P. glacialis* CGMCC 1.8957^T^, *Parasphingomonas echinoides* ATCC 14820^T^, *Parasphingomonas hylomeconis* CCTCC AB 2013304^T^ and *Parasphingomonas qilianensis* CGMCC 1.15349^T^. A phylogenetic tree was constructed based on the PGC sequence (Fig. S5). The topology of the PGC tree showed significant divergence from that of the phylogenomic tree. For instance, strains RT2P30^T^, PB4P5^T^, RB3P16^T^, *P. qilianensis* CGMCC 1.15349^T^, *P. hylomeconis* CCTCC AB 2013304^T^, *P. psychrolutea* CGMCC 1.10106^T^, *P. glacialis* CGMCC 1.8957^T^ and *P. echinoides* ATCC 14820^T^ were present on the same branch in the phylogenomic tree. In contrast, the PGC tree grouped PB4P5^T^, *P. qilianensis* CGMCC 1.15349^T^ and *P. hylomeconis* CCTCC AB 2013304^T^ with PB2P19^T^ and *S. ginsenosidivorax* KHI67^T^ on a distinct branch. The mosaic distribution of PGC across the species tree, combined with differences between the species and PGC trees, suggests a complex evolutionary history for PGC (Fig. 1). This pattern implies that these genes were likely acquired via horizontal gene transfer rather than vertical descent.

## Conclusion

In this study, 12 *Sphingomonadaceae* strains isolated from glaciers in China were identified as novel species using polyphasic taxonomy and phylogenomic analysis. These novel species demonstrated phenotypic diversity, with the ability to utilize multiple carbon sources and the presence of unsaturated fatty acids as the major fatty acid, beneficial for survival in extreme environments. The capacity to metabolize carbohydrates correlated significantly with the number of GHs identified via the CAZy database. Notably, 11 strains could form biofilms, which may influence the distribution and adaptability of *Sphingomonadaceae* in the supraglacial ecological zone. The description of these 12 novel species and their genomic analysis enhances our understanding of the phenotypic and genetic diversity of the family *Sphingomonadaceae* and provides valuable insights into the supraglacial ecosystem.

## Description of *Alteristakelama amylovorans* sp. nov.

*Alteristakelama amylovorans* (a.my.lo.vo’rans. Gr. neut. n. *amylon*, starch; L. pres. part. *vorans*, devouring; N.L. part. adj. *amylovorans*, starch-devouring).

Cells are Gram-negative, aerobic, motile with a single flagellum and rod-shaped, measuring 1.2–2.2×0.7–0.9 µm. Colonies on PYG plates are yellow-coloured, convex and round. Growth occurs at temperatures between 0 and 30 °C, at pH 4.0–11.0 and in the presence of 0–1.0% (w/v) NaCl, with an optimum growth temperature of 20–25 °C. Cells are positive for catalase and oxidase but do not reduce nitrate to nitrite. Do not hydrolyse Tween 80, gelatin or casein, but do hydrolyse starch and aesculin. Indole and H_2_S are not formed. Positive for *β*-galactosidase, alkaline phosphatase, esterase(C4), esterase lipase(C8), lipase(C14), leucine arylamidase, valine arylamidase, cystine arylamidase, trypsin, *α*-chymotrypsin, acid phosphatase, naphthol-AS-BI-phosphohydrolase, *α*-glucosidase, *β*-glucosidase and *N*-acetyl-*β*-glucosaminidase. Utilize the following substances as carbon sources: dextrin, d-cellobiose, gentiobiose, *N*-acetyl-d-glucosamine, d-fructose, d-fucose, l-fucose, l-rhamnose, d-fructose-6-PO_4_, glycyl-l-proline, l-alanine, l-aspartic acid, l-histidine, l-serine, pectin, d-galacturonic acid, l-galactonic acid lactone, d-glucuronic acid, glucuronamide, l-malic acid, bromo-succinic acid, Tween 40, *β*-hydroxy-d,l-butyric acid, acetoacetic acid and acetic acid. Do not produce acid from carbohydrates. Biofilm could be produced during growth. The major fatty acids are summed feature 8 (C_18 : 1_* ω*7c/C_18 : 1_* ω*6c) and C_17 : 1_* ω*6c. The DNA G+C content of the type strain is 64.5 mol%.

The type strain, LT1P40^T^ (=CGMCC 1.11403^T^=KACC 23651^T^), was isolated from a cryoconite sample from Laigu Glacier in Tibet, China. The NCBI accession numbers for the 16S rRNA gene and genome sequences are OR958756 and JAXOJT000000000, respectively.

## Description of *Sphingomonas sorbitolis* sp. nov.

*Sphingomonas sorbitolis* (sor.bi.to’lis. N.L. gen. n. *sorbitolis*, pertaining to sorbitol).

Cells are Gram-negative, aerobic, motile with a single flagellum and rod-shaped, measuring 1.6–2.1×0.9–1.1 µm. Colonies on PYG plates are orange-coloured, convex and round. Growth occurs at temperatures between 0 and 25 °C, at pH 4.0–9.0 and in the presence of 0–1.5% (w/v) NaCl, with an optimum growth temperature of 20–22 °C. Cells are positive for catalase and oxidase but do not reduce nitrate to nitrite. Do not hydrolyse Tween 80, starch, gelatin and casein but do hydrolyse aesculin. Indole and H_2_S are not formed. Positive for citrate utilization, Voges–Proskauer test, alkaline phosphatase, esterase(C4), esterase lipase(C8), lipase(C14), leucine arylamidase, valine arylamidase, cystine arylamidase, trypsin, acid phosphatase, naphthol-AS-BI-phosphohydrolase, *β*-galactosidase, *α*-glucosidase and *β*-glucosidase. Utilize the following substances as carbon sources: dextrin, d-maltose, d-trehalose, d-cellobiose, gentiobiose, sucrose, d-turanose, stachyose, d-raffinose, *α*-d-lactose, d-melibiose, *β*-methyl-d-glucoside, d-salicin, *N*-acetyl-d-glucosamine, *N*-acetyl-*β*-d-mannosamine, *α*-d-glucose, d-mannose, d-fructose, d-galactose, d-sorbitol, d-glucose-6-PO_4_, d-fructose-6-PO_4_, glycyl-l-proline, l-alanine, l-aspartic acid, l-glutamic acid, l-serine, pectin, d-galacturonic acid, l-galactonic acid lactone, d-gluconic acid, glucuronamide, *α*-keto-glutaric acid, l-malic acid, bromo-succinic acid, Tween 40, *β*-hydroxy-d,l-butyric acid, *α*-keto-butyric acid, propionic acid and acetic acid. Acids are produced from d-glucose, d-sucrose and l-arabinose. Biofilm could be produced during growth. The major fatty acids are summed feature 8 (C_18 : 1_* ω*7c/C_18 : 1_* ω*6c) and summed feature 3 (C_16 : 1_* *ω7c/_C16 : 1_* ω*6c). The DNA G+C content of the type strain is 64.9 mol%.

The type strain, LB2R24^T^ (=CGMCC 1.11562^T^=KACC 23650^T^), was isolated from an ice sample from Laigu Glacier in Tibet, China. The NCBI accession numbers for the 16S rRNA gene and genome sequences are OR958757 and JAXOJS000000000, respectively.

## Description of *Sphingomonas fucosidasi* sp. nov.

*Sphingomonas fucosidasi* (fu.co.si.da’si. N.L. gen. n. *fucosidasi*, pertaining to fucosidase).

Cells are Gram-negative, aerobic, motile with a single flagellum and rod-shaped, measuring 1.6–2.9×0.9–1.0 µm. Colonies on PYG plates are orange-coloured, convex and round. Growth occurs at temperatures between 0 and 25 °C, at pH 4.0–9.0 and in the presence of 0–3.0% (w/v) NaCl, with an optimum growth temperature of 20–22 °C. Cells are positive for catalase and oxidase but do not reduce nitrate to nitrite. Do not hydrolyse Tween 80, starch and casein but do hydrolyse gelatin and aesculin. Indole and H_2_S are not formed. Positive for Voges–Proskauer test, alkaline phosphatase, esterase(C4), esterase lipase(C8), lipase(C14), leucine arylamidase, valine arylamidase, cystine arylamidase, trypsin, acid phosphatase, naphthol-AS-BI-phosphohydrolase, *β*-galactosidase, *α*-glucosidase, *β*-glucosidase, *N*-acetyl-*β*-glucosaminidase and *α*-fucosidase. Utilize the following substances as carbon sources: dextrin, d-maltose, d-trehalose, d-cellobiose, gentiobiose, sucrose, d-turanose, stachyose, d-raffinose, *α*-d-lactose, d-melibiose, *β*-methyl-d-glucoside, d-salicin, *N*-acetyl-d-glucosamine, *α*-d-glucose, d-mannose, d-fructose, d-galactose, l-fucose, d-fructose-6-PO_4_, glycyl-l-proline, l-glutamic acid, l-histidine, pectin, d-galacturonic acid, l-galactonic acid lactone, d-gluconic acid, d-glucuronic acid, glucuronamide, methyl pyruvate, *α*-keto-glutaric acid, l-malic acid, bromo-succinic acid, Tween 40 and acetic acid. Acids are produced from l-arabinose. Biofilm could be produced during growth. The major fatty acids are summed feature 8 (C_18 : 1_* ω*7c/C_18 : 1_* ω*6c), summed feature 3 (C_16 : 1_* ω*7c/_C16 : 1_* ω*6c) and C_16 : 0_. The DNA G+C content of the type strain is 65.1 mol%.

The type strain, LB3N6^T^ (=CGMCC 1.11646^T^=KACC 23649^T^), was isolated from an ice sample from Laigu Glacier in Tibet, China. The NCBI accession numbers for the 16S rRNA gene and genome sequences are OR958758 and JAXOJR000000000, respectively.

## Description of *Sphingomonas arabinosi* sp. nov.

*Sphingomonas arabinosi* (a.ra.bi.no’si. N.L. gen. n. *arabinosi*, pertaining to arabinose).

Cells are Gram-negative, aerobic, motile with a single flagellum and rod-shaped, measuring 2.1–3.5×0.9–1.0 µm. Colonies on PYG plates are orange-coloured, convex and round. Growth occurs at temperatures between 0 and 25 °C, at pH 5.0–10.0 and in the presence of 0–1.5% (w/v) NaCl, with an optimum growth temperature of 20–22 °C. Cells are positive for catalase and oxidase but do not reduce nitrate to nitrite. Do not hydrolyse Tween 80, starch and casein but do hydrolyse gelatin and aesculin. Indole and H_2_S are not formed. Positive for Voges–Proskauer test, alkaline phosphatase, leucine arylamidase, valine arylamidase, cystine arylamidase, trypsin, acid phosphatase, naphthol-AS-BI-phosphohydrolase, *α*-galactosidase, *β*-galactosidase, *α*-glucosidase, *β*-glucosidase and *N*-acetyl-*β*-glucosaminidase. Utilize the following substances as carbon sources: dextrin, d-maltose, d-trehalose, d-cellobiose, gentiobiose, sucrose, d-turanose, d-raffinose, *α*-d-lactose, D-melibiose, *β*-methyl-d-glucoside, d-salicin, *N*-acetyl-d-glucosamine, *α*-d-glucose, d-mannose, d-fructose, d-galactose, d-fucose, l-fucose, l-rhamnose, d-fructose-6-PO_4_, gelatin, glycyl-l-proline, l-alanine, l-aspartic acid, l-glutamic acid, l-histidine, pectin, d-galacturonic acid, l-galactonic acid lactone, d-gluconic acid, d-glucuronic acid, glucuronamide, methyl pyruvate, *α*-keto-glutaric acid, l-malic acid, bromo-succinic acid, Tween 40, *β*-hydroxy-d,l-butyric acid, propionic acid, acetic acid and formic acid. Acid is produced from l-arabinose. Biofilm could be produced during growth. The major fatty acids are summed feature 8 (C_18 : 1_* ω*7c/C_18 : 1_* ω*6c), summed feature 3 (C_16 : 1_* ω*7c/_C16 : 1_* ω*6c), C_14 : 0_ 2-OH and C_16 : 0_. The DNA G+C content of the type strain is 64.6 mol%.

The type strain, ZB1N12 ^T^ (=CGMCC 1.23968^T^=KACC 23645^T^), was isolated from an ice sample from Zepu Glacier in Tibet, China. The NCBI accession numbers for the 16S rRNA gene and genome sequences are OR958762 and JAXOJN000000000, respectively.

## Description of *Sphingomonas sandaracina* sp. nov.

*Sphingomonas sandaracina* (san.da.ra.ci’na. Gr. masc. adj. *sandarakinos*, orange; N.L. fem. adj. *sandaracina*, orange).

Cells are Gram-negative, aerobic, motile with a single flagellum and rod-shaped, measuring 1.3–3.0×0.9–1.0 µm. Colonies on PYG plates are orange-coloured, convex and round. Growth occurs at temperatures between 0 and 25 °C, at pH 4.0–8.0 and in the presence of 0–1.5% (w/v) NaCl, with an optimum growth temperature of 20–22 °C. Cells are positive for catalase and oxidase but do not reduce nitrate to nitrite. Do not hydrolyse Tween 80, casein, gelatin and starch but do hydrolyse aesculin. Indole and H_2_S are not formed. Positive for Voges–Proskauer test, alkaline phosphatase, esterase(C4), esterase lipase(C8), lipase(C14), leucine arylamidase, valine arylamidase, cystine arylamidase, trypsin, acid phosphatase, naphthol-AS-BI-phosphohydrolase, *α*-galactosidase, *β*-galactosidase, *α*-glucosidase and *β*-glucosidase. Utilize the following substances as carbon sources: d-maltose, d-trehalose, d-cellobiose, gentiobiose, sucrose, *N*-acetyl-d-glucosamine, *α*-d-glucose, d-mannose, d-fructose, d-galactose, d-fucose, l-fucose, d-fructose-6-PO_4_, gelatin, glycyl-l-proline, l-alanine, l-aspartic acid, l-glutamic acid, pectin, d-galacturonic acid, l-galactonic acid lactone, d-gluconic acid, d-glucuronic acid, glucuronamide, methyl pyruvate, *α*-keto-glutaric acid, l-malic acid, bromo-succinic acid, Tween 40, *β*-hydroxy-d,l-butyric acid and acetic acid. Acids are produced from d-glucose, d-sucrose, d-melibiose, amygdalin and l-arabinose. Biofilm could be produced during growth. The major fatty acids are summed feature 8 (C_18 : 1_* ω*7c/C_18 : 1_* ω*6c) and summed feature 3 (C_16 : 1_* ω*7c/_C16 : 1_* ω*6c). The DNA G+C content of the type strain is 65.0 mol%.

The type strain, PB2P12^T^ (=CGMCC 1.25174^T^=KACC 23642^T^), was isolated from an ice sample from Puruogangri Glacier in Tibet, China. The NCBI accession numbers for the 16S rRNA gene and genome sequences are OR958765 and JAXOJK000000000, respectively.

## Description of *Sphingomonas rhamnosi* sp. nov.

*Sphingomonas rhamnosi* (rham.no’si. N.L. gen. n. *rhamnosi*, pertaining to rhamnose).

Cells are Gram-negative, aerobic, motile with a single flagellum and rod-shaped, measuring 1.1–1.6×0.8–0.9 µm. Colonies on PYG plates are yellow-coloured, convex and round. Growth occurs at temperatures between 0 and 25 °C, at pH 5.0–8.0 and in the presence of 0–1.0% (w/v) NaCl, with an optimum growth temperature of 20–22 °C. Cells are positive for catalase and oxidase but do not reduce nitrate to nitrite. Do not hydrolyse Tween 80, casein, gelatin and starch but do hydrolyse aesculin. Indole and H_2_S are not formed. Positive for Voges–Proskauer test, alkaline phosphatase, esterase(C4), esterase lipase(C8), lipase(C14), leucine arylamidase, valine arylamidase, cystine arylamidase, *α*-chymotrypsin, acid phosphatase, naphthol-AS-BI-phosphohydrolase, *β*-galactosidase, *α*-glucosidase and *β*-glucosidase. Utilize the following substances as carbon sources: dextrin, d-maltose, d-trehalose, D-cellobiose, gentiobiose, d-turanose, d-melibiose, d-salicin, *α*-d-glucose, d-mannose, d-fructose, d-galactose, l-rhamnose, d-fructose-6-PO_4_, glycyl-l-proline, l-glutamic acid, l-histidine, d-glucuronic acid, glucuronamide, methyl pyruvate, *α*-keto-glutaric acid, l-malic acid, bromo-succinic acid, Tween 40, acetoacetic acid and acetic acid. Acid is produced from l-rhamnose. Biofilm could be produced during growth. The major fatty acids are summed feature 8 (C_18 : 1_* ω*7c/C_18 : 1_* ω*6c) and summed feature 3 (C_16 : 1_* ω*7c/_C16 : 1_* ω*6c). The DNA G+C content of the type strain is 66.2 mol%.

The type strain, PB2P19^T^ (=CGMCC 1.25194^T^=KACC 23641^T^), was isolated from an ice sample from Puruogangri Glacier in Tibet, China. The NCBI accession numbers for the 16S rRNA gene and genome sequences are OR958766 and JAXOJJ000000000, respectively.

## Description of *Sphingomonas flavida* sp. nov.

*Sphingomonas flavida* (fla’vi.da. L. fem. adj. *flavida*, pale yellow).

Cells are Gram-negative, aerobic, motile with a single flagellum and rod-shaped, measuring 1.6–3.1×0.7–0.9 µm. Colonies on PYG plates are yellow-coloured, convex and round. Growth occurs at temperatures between 0 and 37 °C, pH 4.0–10.0 and in the presence of 0–1.5% (w/v) NaCl, with an optimum growth temperature of 25–30 °C. Cells are positive for catalase and oxidase but do not reduce nitrate to nitrite. Do not hydrolyse Tween 80, casein, gelatin and starch but do hydrolyse aesculin. Indole and H_2_S are not formed. Positive for citrate utilization, Voges–Proskauer test, alkaline phosphatase, esterase(C4), esterase lipase(C8), leucine arylamidase, valine arylamidase, cystine arylamidase, trypsin, *α*-chymotrypsin, acid phosphatase, naphthol-AS-BI-phosphohydrolase, *α*-galactosidase, *β*-galactosidase, *β*-glucuronidase, *α*-glucosidase, *β*-glucosidase and *N*-acetyl-*β*-glucosaminidase. Utilize the following substances as carbon sources: dextrin, d-maltose, d-trehalose, d-cellobiose, gentiobiose, sucrose, d-turanose, stachyose, d-raffinose, *α*-d-lactose, d-melibiose, *β*-methyl-d-glucoside, d-salicin, *N*-acetyl-d-glucosamine, *N*-acetyl-*β*-d-mannosamine, *α*-d-glucose, d-mannose, d-fructose, d-galactose, d-fucose, l-fucose, d-glucose-6-PO_4_, d-fructose-6-PO_4_, gelatin, glycyl-l-proline, l-alanine, l-glutamic acid, l-histidine, l-serine, pectin, d-galacturonic acid, d-glucuronic acid, glucuronamide, methyl pyruvate, l-lactic acid, *α*-keto-glutaric acid, l-malic acid, bromo-succinic acid, Tween 40, *α*-hydroxy butyric acid, *β*-hydroxy-d,l-butyric acid, *α*-keto-butyric acid, acetoacetic acid, propionic acid and acetic acid. Acids are produced from d-glucose, d-sucrose, d-melibiose, amygdalin and l-arabinose. Biofilm could be produced during growth. The major fatty acid is summed feature 8 (C_18 : 1_* ω*7c/C_18 : 1_* ω*6c). The DNA G+C content of the type strain is 66.2 mol%.

The type strain, PB1R3^T^ (=CGMCC 1.25232^T^=KACC 23639^T^), was isolated from an ice sample from Puruogangri Glacier in Tibet, China. The NCBI accession numbers for the 16S rRNA gene and genome sequences are OR958768 and JAXOJH000000000, respectively.

## Description of *Parasphingomonas frigoris* sp. nov.

*Parasphingomonas frigoris* (fri’go.ris. L. gen. n. *frigoris*, of the cold).

Cells are Gram-negative, aerobic, motile with a single flagellum and rod-shaped, measuring 1.3–2.9×0.8–1.0 µm. Colonies on PYG plates are yellow-coloured, convex and round. Growth occurs at temperatures between 0 and 30 °C, at pH 4.0–9.0 and in the presence of 0–1.5% (w/v) NaCl, with an optimum growth temperature of 20–25 °C. Cells are positive for catalase and oxidase but do not reduce nitrate to nitrite. Do not hydrolyse Tween 80, starch, gelatin and casein but do hydrolyse aesculin. Indole and H_2_S are not formed. Positive for tryptophan deaminase, Voges–Proskauer test, alkaline phosphatase, esterase(C4), esterase lipase(C8), lipase(C14), leucine arylamidase, valine arylamidase, cystine arylamidase, *α*-chymotrypsin, acid phosphatase, naphthol-AS-BI-phosphohydrolase, *β*-galactosidase, *β*-glucuronidase, *α*-glucosidase and *β*-glucosidase. Utilize the following substances as carbon sources: dextrin, d-maltose, d-cellobiose, gentiobiose, sucrose, d-turanose, *N*-acetyl-d-glucosamine, *α*-d-glucose, d-mannose, d-fructose, d-galactose, d-fucose, l-rhamnose, d-fructose-6-PO_4_, gelatin, glycyl-l-proline, l-alanine, l-aspartic acid, l-glutamic acid, l-histidine, pectin, d-galacturonic acid, d-glucuronic acid, glucuronamide, quinic acid, l-malic acid, bromo-succinic acid, Tween 40, *β*-hydroxy-d,l-butyric acid, *α*-keto-butyric acid and acetoacetic acid. Acids are produced from d-glucose, l-rhamnose, d-sucrose, amygdalin and l-arabinose. Biofilm could be produced during growth. The major fatty acids are summed feature 8 (C_18 : 1_* ω*7c/C_18 : 1_* ω*6c) and C_16 : 0_. The DNA G+C content of the type strain is 66.2 mol%.

The type strain, RB3P16^T^ (=CGMCC 1.11860^T^=KACC 23648^T^), was isolated from an ice sample from Renlongba Glacier in Tibet, China. The NCBI accession numbers for the 16S rRNA gene and genome sequences are OR958759 and JAXOJQ000000000, respectively.

## Description of *Parasphingomonas halimpatiens* sp. nov.

*Parasphingomonas halimpatiens* (hal.im.pa'ti.ens. L. neut. n. *hals*, salt; L. masc. adj. *impatiens*, intolerant; N.L. fem. adj. *halimpatiens*, salt-intolerant)

Cells are Gram-negative, aerobic, motile with a single flagellum and rod-shaped, measuring 1.3–2.2×0.7–0.9 µm. Colonies on PYG plates are yellow-coloured, convex and round. Growth occurs at temperatures between 0 and 35 °C, at pH 5.0–8.0 and in the presence of 0–0.05% (w/v) NaCl, with an optimum growth temperature of 25–30 °C. Cells are positive for catalase and oxidase but do not reduce nitrate to nitrite. Do not hydrolyse Tween 80, starch, gelatin and casein but do hydrolyse aesculin. Indole and H_2_S are not formed. Positive for alkaline phosphatase, esterase(C4), esterase lipase(C8), lipase(C14), leucine arylamidase, valine arylamidase, cystine arylamidase, *α*-chymotrypsin, acid phosphatase, naphthol-AS-BI-phosphohydrolase, *α*-galactosidase, *β*-galactosidase, *β*-glucuronidase, *α*-glucosidase and *β*-glucosidase. Utilize the following substances as carbon sources: l-arabinose, d-xylose, galactose, glucose, aesculin, d-cellobiose, d-maltose, d-lactose, d-sucrose, gentiobiose, d-turanose and potassium gluconate. Acids are produced from d-glucose, d-sucrose and l-arabinose. Biofilm could be produced during growth. The major fatty acids are summed feature 8 (C_18 : 1_* ω*7c/C_18 : 1_* ω*6c), C_16 : 0_, C_14 : 0_ 2-OH and C_16 : 1_* ω*5c. The DNA G+C content of the type strain is 65.8 mol%.

The type strain, RT2P30^T^ (=CGMCC 1.23559^T^=KACC 23647^T^), was isolated from a cryoconite sample from Renlongba Glacier in Tibet, China. The NCBI accession numbers for the 16S rRNA gene and genome sequences are OR958760 and JAXOJP000000000, respectively.

## Description of *Parasphingomonas zepuensis* sp. nov.

*Parasphingomonas zepuensis* (ze.pu.en’sis. N.L. fem. adj. *zepuensis*, pertaining to Zepu Glacier).

Cells are Gram-negative, aerobic, motile with a single flagellum and rod-shaped, measuring 1.3–2.6×0.7–0.8 µm. Colonies on PYG plates are yellow-coloured, convex and round. Growth occurs at temperatures between 0 and 35 °C, at pH 4.0–11.0 and in the presence of 0–1.0% (w/v) NaCl, with an optimum growth temperature of 25–30 °C. Cells are positive for catalase and oxidase but do not reduce nitrate to nitrite. Do not hydrolyse Tween 80, starch, gelatin and casein but do hydrolyse aesculin. Indole and H_2_S are not formed. Positive for *β*-galactosidase, Voges–Proskauer test, alkaline phosphatase, esterase(C4), esterase lipase(C8), leucine arylamidase, valine arylamidase, cystine arylamidase, acid phosphatase, naphthol-AS-BI-phosphohydrolase, *α*-glucosidase, *β*-glucosidase and *N*-acetyl-*β*-glucosaminidase. Utilize the following substances as carbon sources: d-trehalose, d-cellobiose, sucrose, *N*-acetyl-d-glucosamine, *N*-acetyl-d-galactosamine, *α*-d-glucose, d-mannose, d-fucose, l-rhamnose, d-fructose-6-PO_4_, glycyl-l-proline, l-glutamic acid, l-histidine, pectin, d-galacturonic acid, l-galactonic acid lactone, d-gluconic acid, d-glucuronic acid, glucuronamide, quinic acid, l-lactic acid, l-malic acid, bromo-succinic acid, Tween 40, *β*-hydroxy-d,l-butyric acid, acetic acid and formic acid. Acid is produced from l-rhamnose. The major fatty acids are summed feature 8 (C_18 : 1_* ω*7c/C_18 : 1_* ω*6c), C_16 : 0_ and C_14 : 0_ 2-OH. The DNA G+C content of the type strain is 65.6 mol%.

The type strain, ZT3P38 ^T^ (=CGMCC 1.23914^T^=KACC 23646^T^), was isolated from a cryoconite sample from Zepu Glacier in Tibet, China. The NCBI accession numbers for the 16S rRNA gene and genome sequences are OR958761 and JAXOJO000000000, respectively.

## Description of *Parasphingomonas caseinilytica* sp. nov.

*Parasphingomonas caseinilytica* [ca.se.i.ni.ly’ti.ca. N.L. neut. n. *caseinum*, casein; N.L. masc. adj. *lyticus* (from Gr. masc. adj. *lytikos*), dissolving; N.L. fem. adj. *caseinilytica*, casein-dissolving].

Cells are Gram-negative, aerobic, motile with a single flagellum and rod-shaped, measuring 1.4–2.4×0.6–0.7 µm. Colonies on PYG plates are yellow-coloured, convex and round. Growth occurs at temperatures between 0 and 25 °C, at pH 4.0–9.0 and in the presence of 0–1.0% (w/v) NaCl, with an optimum growth temperature of 20–22 °C. Cells are positive for catalase and oxidase but do not reduce nitrate to nitrite. Do not hydrolyse Tween 80 and starch but do hydrolyse aesculin, gelatin and casein. Indole and H_2_S are not formed. Positive for *β*-galactosidase, Voges–Proskauer test, alkaline phosphatase, esterase(C4), esterase lipase(C8), leucine arylamidase, valine arylamidase, cystine arylamidase, trypsin, *α*-chymotrypsin, acid phosphatase, naphthol-AS-BI-phosphohydrolase, *α*-glucosidase and *N*-acetyl-*β*-glucosaminidase. Utilize the following substances as carbon sources: dextrin, d-maltose, d-trehalose, sucrose, *N*-acetyl-d-glucosamine, *α*-d-glucose, d-mannose, d-fructose, d-galactose, d-fucose, l-fucose, glycerol, d-fructose-6-PO_4_, gelatin, glycyl-l-proline, l-alanine, l-aspartic acid, l-glutamic acid, l-histidine, pectin, glucuronamide, quinic acid, methyl pyruvate, *α*-keto-glutaric acid, l-malic acid, bromo-succinic acid, Tween 40 and *β*-hydroxy-d,l-butyric acid. Biofilm could be produced during growth. The major fatty acids are summed feature 8 (C_18 : 1_* ω*7c/C_18 : 1_* ω*6c), C_16 : 0_ and C_14 : 0_ 2-OH. The DNA G+C content of the type strain is 64.4 mol%.

The type strain, GB1N7^T^ (=CGMCC 1.24759^T^=KACC 23643^T^), was isolated from an ice sample from Gawalong Glacier in Tibet, China. The NCBI accession numbers for the 16S rRNA gene and genome sequences are OR958764 and JAXOJL000000000, respectively.

## Description of *Parasphingomonas puruogangriensis* sp. nov.

*Parasphingomonas puruogangriensis* (pu.ru.o.gan.gri.en’sis. N.L. fem. adj. *puruogangriensis*, from Puruogangri).

Cells are Gram-negative, aerobic, motile with a single flagellum and rod-shaped, measuring 1.7–2.4×0.7–0.9 µm. Colonies on PYG plates are yellow-coloured, convex and round. Growth occurs at temperatures between 0 and 25 °C, at pH 5.0–9.0 and in the presence of 0–0.5% (w/v) NaCl, with an optimum growth temperature of 20–22 °C. Cells are positive for catalase and oxidase but do not reduce nitrate to nitrite. Do not hydrolyse Tween 80, casein, gelatin and starch but do hydrolyse aesculin. Indole and H_2_S are not formed. Positive for alkaline phosphatase, esterase(C4), esterase lipase(C8), lipase(C14), leucine arylamidase, valine arylamidase, cystine arylamidase, trypsin, *α*-chymotrypsin, acid phosphatase, naphthol-AS-BI-phosphohydrolase, *α*-galactosidase, *β*-galactosidase and *β*-glucosidase. Utilize the following substances as carbon sources: gentiobiose, sucrose, d-turanose, stachyose, *α*-d-lactose, d-melibiose, d-salicin, *N*-acetyl-d-glucosamine, d-galactose, d-fucose, l-fucose, d-fructose-6-PO_4_, d-galacturonic acid, l-galactonic acid lactone, glucuronamide, *α*-keto-glutaric acid, l-malic acid and *β*-hydroxy-d,l-butyric acid. Biofilm could be produced during growth. The major fatty acids are summed feature 8 (C_18 : 1_* ω*7c/C_18 : 1_* ω*6c), summed feature 3 (C_16 : 1_* ω7*c/_C16 : 1_* ω*6c) and C_16 : 0_. The DNA G+C content of the type strain is 64.8 mol%.

The type strain, PB4P5^T^ (=CGMCC 1.25204^T^=KACC 23640^T^), was isolated from an ice sample from Puruogangri Glacier in Tibet, China. The NCBI accession numbers for the 16S rRNA gene and genome sequences are OR958767 and JAXOJI000000000, respectively.

## Supplementary material

10.1099/ijsem.0.006913 Uncited Supplementary Material 1.

## Abbreviations

ANI: average nt identity
CAZyme: carbohydrate-active enzyme
dDDH: digital DNA–DNA hybridization
GH: glycoside hydrolase
PYG: peptone, yeast extract and glucose
