# Supplementary material for: Diverse biofilm-forming Sphingomonadaceae represent twelve novel species isolated from glaciers on the Tibetan Plateau
Source: Int J Syst Evol Microbiol. 2025 Sep 8;75(9):006913. doi: 10.1099/ijsem.0.006913 (PMC12417090; doi:10.1099/ijsem.0.006913)
Supplement: Uncited Supplementary Material 1. [file ijsem-75-06913-s001.pdf]

Supplementary Material

Supplementary Table S4. Phenotypic characteristics of the type strains of 12 novel *Sphingomonas* species and their closely relatives. NA indicates that this feature was not mentioned in the original literature.

**Strain**	**Temperature for growth (°C)**	**pH for growth**	**NaCl Tolerance (%, w/v)**	**Colony color**	**Cell size (µm)**	**Flagellum**
LT1P40^T	0-30	4-11	0-1.0	yellow	1.2-2.2 × 0.7-0.9	+
A. koreensis NBRC 16723^T (Lee et al., 2001)	NA	NA	< 3.0	yellow	NA	+
LB2R24^T	0-25	4-9	0-1.5	orange	1.6-2.1 × 0.9-1.1	+
S. faeni MA-olki^T (Busse et al., 2003)	4-28	NA	NA	orange	2.0-2.6 × 0.6-0.8	NA
LB3N6^T	0-25	4-9	0-3.0	orange	1.6-2.9 × 0.9-1.0	+
RB3P16^T	0-30	4-9	0-1.5	yellow	1.3-2.9 × 0.8-1.0	+
P. glacialis C16y^T (Zhang et al., 2011)	1-30	7-8	0-1.0	yellow-orange	0.5 × 0.8	NA
P. psychrolutea MDB1-A^T (Liu et al., 2015)	0-25	5-8	< 1.0	orange–yellow	1.8–2.2 × 0.5–0.6	-
RT2P30^T	0-35	5-8	0-0.05	yellow	1.3-2.2 × 0.7-0.9	+
S. oligophenolica JCM 12082^T (Ohta et al., 2004)	4-37	NA	0.1-0.4	yellow	1.0-1.5 × 0.4-0.6	NA
P. echinoides ATCC 14820^T (Denner et al., 1999)	NA	NA	NA	yellow	0.8×1.9	+
ZT3P38^T	0-35	4-11	0-1.0	yellow	1.3-2.6 × 0.7-0.8	+
ZB1N12^T	0-25	5-10	0-1.5	orange	2.1-3.5 × 0.9-1.0	+
S. aurantiaca MA101b^T (Busse et al., 2003)	4-28	NA	NA	orange	1.0–2.7 × 0.6–0.8	NA

GB1N7^{T}	0-25	4-9	0-1.0	yellow	1.4-2.4 × 0.6-0.7	+
P. aliaeris DH-S5^{T} (Heidler von Heilborn et al., 2021)	3–33	6-7	0-0.25	red	1.5 × 0.9	-
PB2P12^{T}	0-25	4-8	0-1.5	orange	1.3-3.0 × 0.9-1.0	+
PB2P19^{T}	0-25	5-8	0-1.0	yellow	1.1-1.6 × 0.8-0.9	+
S. aerolata NW12^{T} (Busse et al., 2003)	4-28	NA	NA	orange	1.5–2.6 × 0.6–0.8	NA
S. ginsenosidivorax KHI67^{T} (Jin et al., 2013)	4-37	5.5-8.5	0-1.0	yellowish	1.5-2.2 × 0.3–0.5	NA
PB4P5^{T}	0-25	5-9	0-0.5	yellow	1.7-2.4 × 0.7-0.9	+
P. qilianensis CGMCC 1.15349^{T} (Piao et al., 2016)	15–30	6–10	0–0.3	light-yellow	0.4 × 1.2–2.0	NA
P. hylomeconis GZJT-2^{T} (Akbar et al., 2015)	4-30	6-8	0-1.0	yellow	1.4-2.2 × 0.6-0.7	-
PB1R3^{T}	0-37	4-10	0-1.5	yellow	1.6-3.1 × 0.7-0.9	+
S. sanguinis NBRC 13937^{T} (Takeuchi et al., 1993)	NA	NA	NA	deep yellow	NA	+

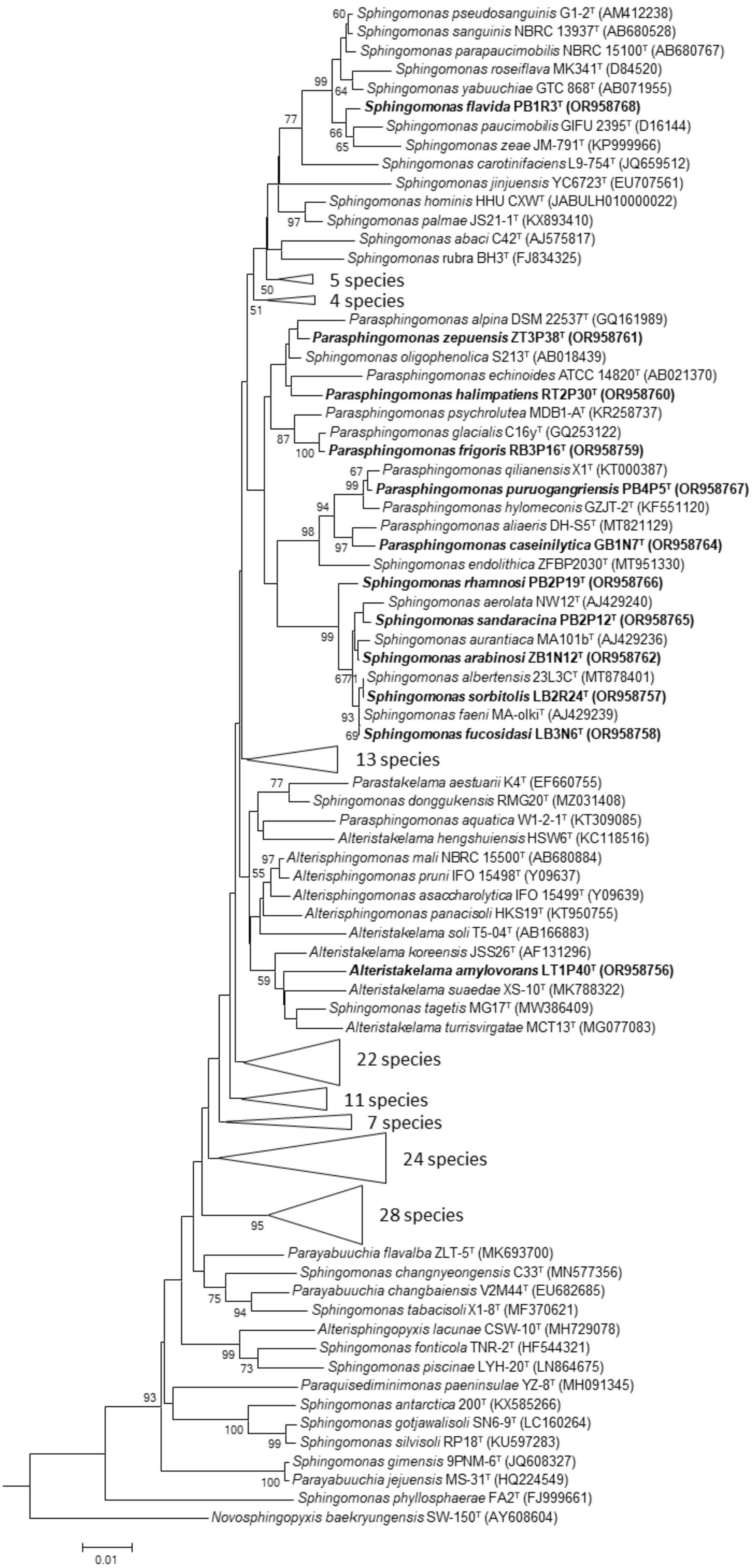

Sphingomonas pseudosanguinis G1-2T (AM412238)
Sphingomonas sanguinis NBRC 13937T (AB680528)
Sphingomonas parapaucimobilis NBRC 15100T (AB680767)
Sphingomonas roseiflava MK341T (D84520)
Sphingomonas yabuuchiae GTC 868T (AB071955)
Sphingomonas flavida PB1R3T (OR958768)
Sphingomonas paucimobilis GIFU 2395T (D16144)
Sphingomonas zeae JM-791T (KP999966)
Sphingomonas carotinifaciens L9-754T (JQ659512)
Sphingomonas jinjuensis YC6723T (EU707561)
Sphingomonas hominis HHU CXWT (JABULH010000022)
Sphingomonas palmae JS21-1T (KX893410)
Sphingomonas abaci C42T (AJ575817)
Sphingomonas rubra BH3T (FJ834325)
5 species
4 species
Parasphingomonas alpina DSM 22537T (GQ161989)
Parasphingomonas zepuensis ZT3P38T (OR958761)
Sphingomonas oligophenolica S213T (AB018439)
Parasphingomonas echinoides ATCC 14820T (AB021370)
Parasphingomonas halimpatiens RT2P30T (OR958760)
Parasphingomonas psychrolutea MDB1-AT (KR258737)
Parasphingomonas glacialis C16yT (GQ253122)
Parasphingomonas frigoris RB3P16T (OR958759)
Parasphingomonas qilianensis X1T (KT000387)
Parasphingomonas puruogangriensis PB4P5T (OR958767)
Parasphingomonas hylomeconis GZJT-2T (KF551120)
Parasphingomonas aliaeris DH-S5T (MT821129)
Parasphingomonas caseinilytica GB1N7T (OR958764)
Sphingomonas endolithica ZFBP2030T (MT951330)
Sphingomonas rhamnosi PB2P19T (OR958766)
Sphingomonas aerolata NW12T (AJ429240)
Sphingomonas sandaracina PB2P12T (OR958765)
Sphingomonas aurantiaca MA101bT (AJ429236)
Sphingomonas arabinosi ZB1N12T (OR958762)
Sphingomonas albertensis 23L3CT (MT878401)
Sphingomonas sorbitolis LB2R24T (OR958757)
Sphingomonas faeni MA-olkiT (AJ429239)
Sphingomonas fucosidasi LB3N6T (OR958758)
13 species
Parastakelama aestuarii K4T (EF660755)
Sphingomonas donggukensis RMG20T (MZ031408)
Parasphingomonas aquatica W1-2-1T (KT309085)
Alteristakelama hengshuiensis HSW6T (KC118516)
Alterisphingomonas mali NBRC 15500T (AB680884)
Alterisphingomonas pruni IFO 15498T (Y09637)
Alterisphingomonas asaccharolytica IFO 15499T (Y09639)
Alterisphingomonas panacisoli HKS19T (KT950755)
Alteristakelama soli T5-04T (AB166883)
Alteristakelama koreensis JSS26T (AF131296)
Alteristakelama amylovorans LT1P40T (OR958756)
Alteristakelama suaedae XS-10T (MK788322)
Sphingomonas tagetis MG17T (MW386409)
Alteristakelama turrisvirgatae MCT13T (MG077083)
22 species
11 species
7 species
24 species
28 species
Parayabuuchia flavalba ZLT-5T (MK693700)
Sphingomonas changnyeongensis C33T (MN577356)
Parayabuuchia changbaiensis V2M44T (EU682685)
Sphingomonas tabacisoli X1-8T (MF370621)
Alterisphingopyxis lacunae CSW-10T (MH729078)
Sphingomonas fonticola TNR-2T (HF544321)
Sphingomonas piscinae LYH-20T (LN864675)
Paraquisediminimonas paeninsulae YZ-8T (MH091345)
Sphingomonas antarctica 200T (KX585266)
Sphingomonas gotjawalisoli SN6-9T (LC160264)
Sphingomonas silvisoli RP18T (KU597283)
Sphingomonas gimensis 9PNM-6T (JQ608327)
Parayabuuchia jejuensis MS-31T (HQ224549)
Sphingomonas phyllosphaerae FA2T (FJ999661)
Novosphingopyxis baekryungensis SW-150T (AY608604)
0.01

Supplementary Fig. S1. Phylogenetic tree of the twelve strains and related type strains based on the 16S rRNA gene sequence comparisons using the NJ method. GenBank accession numbers of the 16S rRNA gene sequences are given in parentheses. All ambiguous positions were removed for each sequence pair. There were a total of 1345 positions in the final dataset. Bootstrap values (>50 %) based on 1,000 replicates are shown at the branch nodes. Bar, 0.01 substitutions per nucleotide positions.

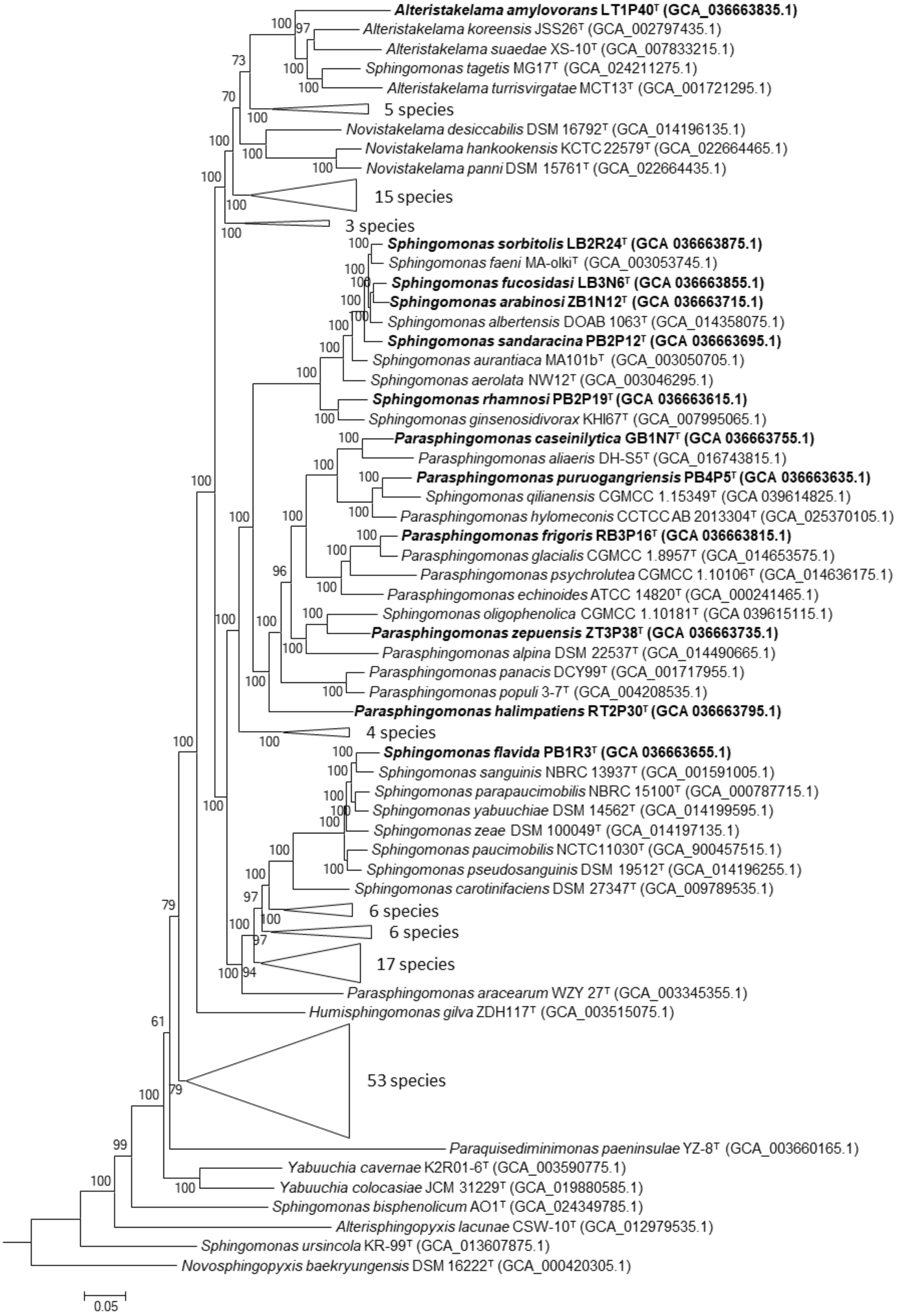

Supplementary Fig. S2. Phylogenomic tree, constructed based on 92 core genes, delineates the evolutionary relationships among the twelve novel strains and the known species of the genus *Sphingomonas*. Genomic sequence accession numbers are given in parentheses. *Novosphingopyxis baekryungensis* DSM 16222^{T} was used as an outgroup. The 92 core genes were *alaS*, *argS*, *aspS*, *cgtA*, *coaE*, *cysS*, *dnaA*, *dnaG*, *dnaX*, *engA*, *ffh*, *fmt*, *frr*, *ftsY*, *gmk*, *hisS*, *ileS*, *infB*, *infC*, *ksgA*,

lepA, *leuS*, *ligA*, *nusA*, *nusG*, *pgk*, *pheS*, *pheT*, *prfA*, *pyrG*, *rbfA*, *recA*, *rnc*, *rplA*, *rplB*, *rplC, rplD,* *rplE*, *rplF*, *rplI*, *rplJ*, *rplK*, *rplL*, *rplM*, *rplN*, *rplO*, *rplP*, *rplQ*, *rplR*, *rplS*, *rplT*, *rplU*, *rplV*, *rplW*, *rplX*, *rpmA*, *rpmC*, *rpmI*, *rpoA*, *rpoB*, *rpoC*, *rpsB*, *rpsC*, *rpsD*, *rpsE*, *rpsF*, *rpsG*, *rpsH*, *rpsI*, *rpsJ*, *rpsK*, *rpsL, rpsM*, *rpsO*, *rpsP*, *rpsQ*, *rpsR*, *rpsS*, *rpsT*, *secA*, *secG*, *secY*, *serS*, *smpB*, *tig*, *tilS*, *truB*, *tsaD*, *tsf*, *uvrB*, *ybeY*, *and ychF*, respectively.

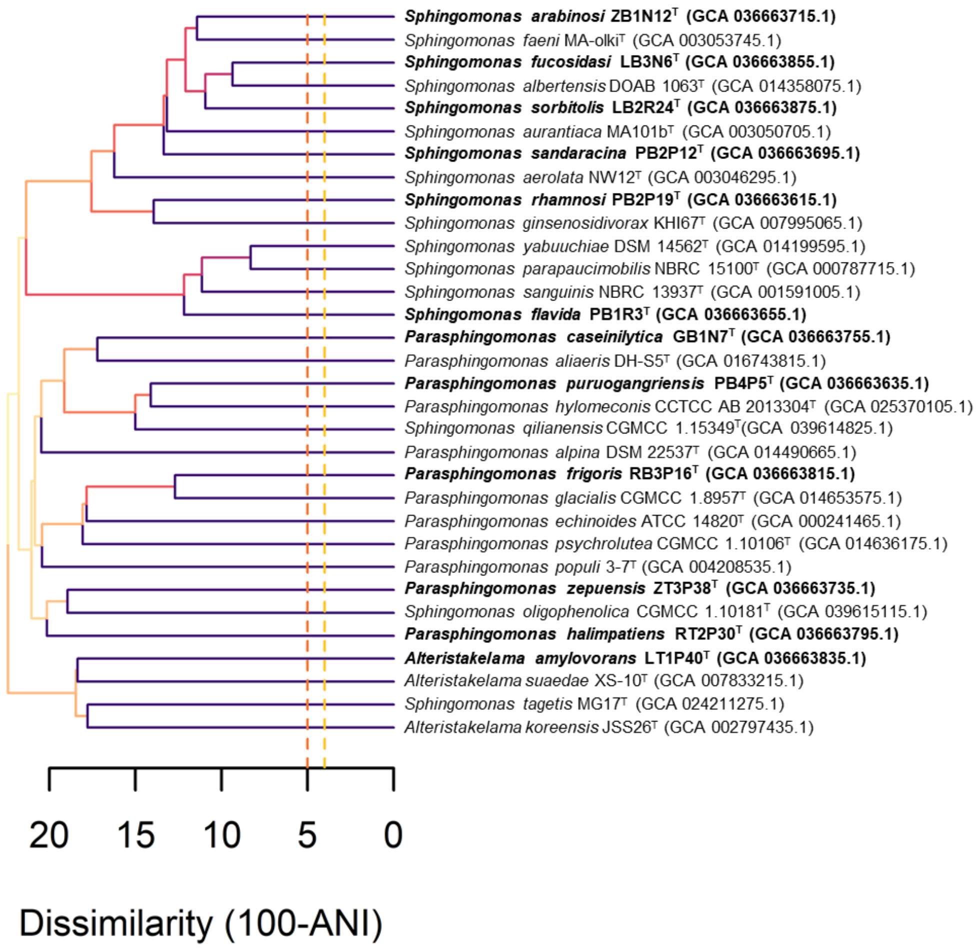

Supplementary Fig. S3. Cluster analysis based on pairwise ANI values of the twelve strains and their related relatives of genus *Sphingomonas*. Accession numbers of the genomic sequences are given in parentheses.

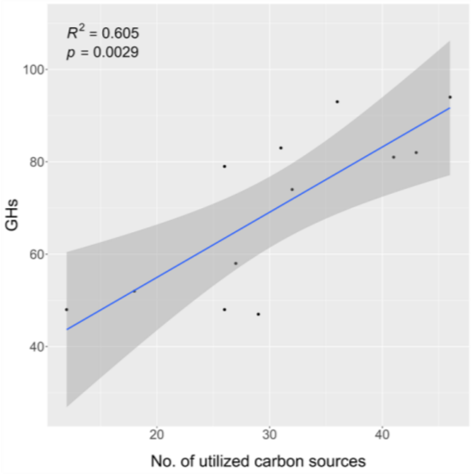

Supplementary Fig. 4. Correlation between carbon source utilization capacity and the number of GH-encoding genes assessed using the Pearson correlation coefficient.

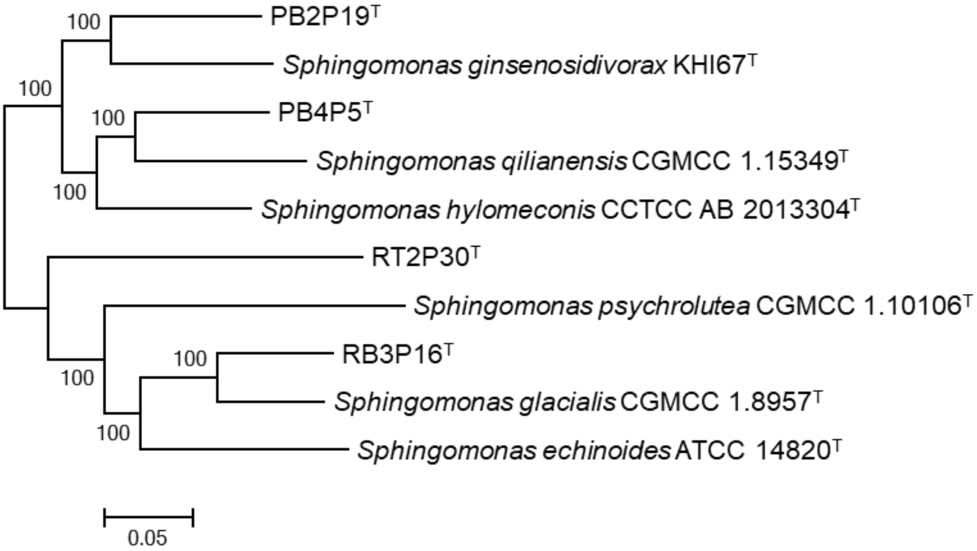

Supplementary Fig. S5. The maximum-likelihood phylogenetic tree constructed based on biosynthesis and photosynthesis gene cluster genes using IQ-TREE software with 1,000 bootstrap replicates, employing the best model of GTR+F+I+G4.

Reference:

Akbar, A., Chen, C., Zhu, L., Xin, K., Cheng, J., Yang, Q., et al. (2015). *Sphingomonas hylomeconis* sp. nov., isolated from the stem of Hylomecon japonica. Int J Syst Evol Microbiol. 65(11): 4025-4031.

doi: 10.1099/ijsem.0.000532.
Busse, H. J., Denner, E. B. M., Buczolits, S., Salkinoja-Salonen, M., Bennasar, A., Kampfer, P. (2003). *Sphingomonas aurantiaca* sp. nov., *Sphingomonas aerolata* sp. nov. and *Sphingomonas faeni* sp. nov., air- and dustborne and Antarctic, orange-pigmented, psychrotolerant bacteria, and emended description of the genus *Sphingomonas*. Int J Syst Evol Microbiol. 53(Pt 5): 1253-1260. doi: 10.1099/ijs.0.02461-0.
Denner, E. B., Kampfer, P., Busse, H. J., Moore, E. R. (1999). Reclassification of *Pseudomonas echinoides* Heumann 1962, 343AL, in the genus *Sphingomonas* as *Sphingomonas echinoides* comb. nov. Int J Syst Bacteriol. 49(Pt 3): 1103-1109. doi: 10.1099/00207713-49-3-1103.
Heidler von Heilborn, D., Reinmuller, J., Holzl, G., Meier-Kolthoff, J. P., Woehle, C., Marek, M., et al. (2021). *Sphingomonas aliaeris* sp. nov., a new species isolated from pork steak packed under modified atmosphere. Int J Syst Evol Microbiol. 71(8). doi: 10.1099/ijsem.0.004973.
Jin, X. F., Kim, J. K., Liu, Q. M., Kang, M. S., He, D., Jin, F. X., et al. (2013). *Sphingomonas ginsenosidivorax* sp. nov., with the ability to transform ginsenosides. Antonie Van Leeuwenhoek. 103(6): 1359-1367. doi: 10.1007/s10482-013-9916-2.
Lee, J. S., Shin, Y. K., Yoon, J. H., Takeuchi, M., Pyun, Y. R., Park, Y. H. (2001). *Sphingomonas aquatilis* sp. nov., *Sphingomonas koreensis* sp. nov., and *Sphingomonas taejonensis* sp. nov., yellow-pigmented bacteria isolated from natural mineral water. 51(Pt 4): 1491-1498. doi: 10.1099/00207713-51-4-1491.
Liu, Q., Liu, H. C., Zhang, J. L., Zhou, Y. G., Xin, Y. H. (2015). *Sphingomonas psychrolutea* sp. nov., a psychrotolerant bacterium isolated from glacier ice. Int J Syst Evol Microbiol. 65(9): 2955-2959. doi: 10.1099/ijs.0.000362.
Ohta, H., Hattori, R., Ushiba, Y., Mitsui, H., Ito, M., Watanabe, H., et al. (2004). *Sphingomonas oligophenolica* sp. nov., a halo- and organo-sensitive oligotrophic bacterium from paddy soil that degrades phenolic acids at low concentrations. Int J Syst Evol Microbiol. 54(Pt 6): 2185-2190. doi: 10.1099/ijs.0.02959-0.
Piao, A. L., Feng, X. M., Nogi, Y., Han, L., Li, Y., Lv, J. (2016). *Sphingomonas qilianensis* sp. nov., isolated from surface soil in the permafrost region of Qilian Mountains, China. Curr Microbiol. 72(4): 363-369. doi: 10.1007/s00284-015-0957-9.
Takeuchi, M., Kawai, F., Shimada, Y., Yokota, A. (1993). Taxonomic study of polyethylene glycol-utilizing bacteria: emended description of the genus *Sphingomonas* and new descriptions of *Sphingomonas macrogoltabidus* sp. nov., *Sphingomonas sanguis* sp. nov., and *Sphingomonas terrae* sp. nov. Syst Appl Microbiol. 16(2): 227-238. doi: 10.1016/S0723-2020(11)80473-X.
Zhang, D. C., Busse, H. J., Liu, H. C., Zhou, Y. G., Schinner, F., Margesin, R. (2011). *Sphingomonas glacialis* sp. nov., a psychrophilic bacterium isolated from alpine glacier cryoconite. Int J Syst Evol Microbiol. 61(Pt 3): 587-591. doi: 10.1099/ijs.0.023135-0.

Table S1. The basic information of the genomic sequences analyzed in this study.

Strains	GenBank Accession No.	Contigs	Total length (Mb)	GC (%)	N50	Gene	CDS	misc RNA	rRNA	tRNA	tmRNA	Repeat region	Completeness (%)	Contamination (%)
PB1R3^{T}	JAXOJH000000000	66	4.07	66.2	210355	3749	3673	18	3	54	1	1	99.99	0.97
S. sanguinis NBRC 13937^{T}	GCA_001591005.1	134	4.05	66.1	61922	3853	3780	21	3	47	2	-	99.59	1.11
S. yabuuchiae DSM 14562^{T}	GCA_014199595.1	58	4.19	66.0	212136	3949	3864	20	3	60	2	-	99.59	0.51
S. parapaucimobilis NBRC 15100	GCA_000787715.1	127	3.99	66.4	68960	3745	3665	19	5	55	1	4	99.59	0.87
PB2P19^{T}	JAXOJJ000000000	35	3.9	66.2	463875	3695	3613	25	3	53	1	-	100	0.89
S. ginsenosidivorax KHI67^{T}	GCA_007995065.1	2	4.14	67.3	3958997	3854	3766	22	9	56	1	-	99.55	1.93
S. aurantiaca MA101b^{T}	GCA_003050705.1	13	4.41	66.2	577097	4041	3959	24	4	53	1	-	99.66	1.32
PB2P12^{T}	JAXOJK000000000	17	4.15	65.0	771424	3816	3745	18	3	49	1	1	100	0.63
LB2R24^{T}	JAXOJS000000000	25	4.62	64.9	605728	4206	4128	18	3	55	2	-	100	0.66
S. faeni MA-olki^{T}	GCA_003053745.1	33	4.38	64.8	367492	4095	4012	21	3	58	1	-	99.66	0.56
ZB1N12^{T}	JAXOJN000000000	63	4.46	64.6	310158	4151	4069	21	3	57	1	-	100	1.06
LB3N6^{T}	JAXOJR000000000	32	4.57	65.1	365609	4171	4092	21	3	54	1	-	100	1.12
S. albertensis DOAB 1063^{T}	GCA_014358075.1	80	4.06	65.7	86121	3734	3662	19	3	49	1	-	95.17	0.2
S. aerolata NW12^{T}	GCA_003046295.1	5	3.83	66.5	1133482	3453	3379	16	3	54	1	-	99.66	0.54
RT2P30^{T}	JAXOJP000000000	93	5.53	65.8	132746	5270	5199	13	3	54	1	-	99.99	1.94
P. populi 3-7^{T}	GCA_004208535.1	47	5.20	65.1	106721	4897	4795	43	3	55	1	-	99.66	4.58
P. psychrolutea CGMCC 1.10106^{T}	GCA_014636175.1	58	3.75	64.2	126669	3627	3561	12	3	50	1	-	98.91	0.46
RB3P16^{T}	JAXOJQ000000000	106	4.75	66.2	892377	4505	4419	29	3	53	1	-	100	1.88
P. glacialis CGMCC 1.8957^{T}	GCA_014653575.1	58	4.47	65.7	863377	4268	4189	25	3	50	1	-	99.57	1.31
P. echinoides ATCC 14820^{T}	GCA_000241465.1	6	4.26	64.7	3995551	4036	3968	16	3	48	1	-	98.98	1.21
GB1N7^{T}	JAXOJL000000000	25	4.28	64.4	335245	3991	3912	22	6	50	1	-	99.99	1.27
P. aliaeris DH-S5^{T}	GCA_016743815.1	3	4.26	64.4	3916662	4232	4160	12	6	53	1	-	97.34	0.94
P. hylomeconis CCTCC AB 2013	GCA_025370105.1	34	3.90	66.6	298021	3721	3656	12	3	49	1	-	99.54	0.44
PB4P5^{T}	JAXOJI000000000	47	4.37	64.8	499562	4209	4144	15	4	45	1	-	99.99	0.71
P. qilianensis CGMCC 1.15349^{T}	JBDIMF000000000	10	3.42	64.7	480537	3E+05	3216	10	3	51	1	2	99.97	0.09
P. alpina DSM 22537^{T}	GCA_014490665.1	1	5.20	64.0	5197460	4902	4830	14	3	54	1	-	99.13	2.01
ZT3P38^{T}	JAXOJO000000000	103	5.41	65.6	817466	5030	4957	18	3	51	1	-	100	2.32
S. oligophenolica CGMCC 1.101	JBDIME000000000	77	6.26	64.8	141185	85032	5685	14	3	53	1	-	100	1.08
LT1P40^{T}	JAXOJT000000000	2	3.47	64.5	2336855	3393	3338	11	3	51	1	-	99.96	0.08
S. tagetis MG17^{T}	GCA_024211275.1	58	4.68	66.05	229323	4483	4555	15	3	53	1	-	98.79	2.15
A. suaedae XS-10^{T}	GCA_007833215.1	1	4.15	65.53	4154291	3904	3968	10	3	50	1	-	99.37	0.8
A. koreensis JSS26^{T}	GCA_002797435.1	1	4.40	66.14	4398689	4171	4241	13	6	50	1	-	99.25	1.9

Table S2. The ANI values between the twelve isolates and the known species.

Strains	Reference strain	ANI (%)
LT1P40	Sphingomonas tagetis MG17 GCA 024211275.1	82.47
LT1P40	Alteristakelama koreensis JSS26 GCA 002797435.1	81.75
LT1P40	Alteristakelama suaedae XS-10 GCA 007833215.1	81.73
LT1P40	Alteristakelama turrisvirgatae MCT13 GCA 001721295.1	80.74
LT1P40	Alteristakelama hengshuiensis WHSC-8 GCA 000935025.1	79.43
LT1P40	Alteristakelama naasensis DSM 100060 GCA 011762145.1	79.25
LT1P40	Sphingomonas donggukensis RMG20 GCA 023674425.1	79.19
LT1P40	Alteristakelama kyeonggiensis DSM 101806 GCA 014196745.1	79.13
LT1P40	Sphingomonas qomolangmaensis S5-59 GCA 024496245.1	79.11
LT1P40	Alteristakelama soli NBRC 100801 GCA 001591025.1	79.08
LT1P40	**GB1N7**	79.06
LT1P40	Sphingomonas caeni LB-2 GCA 026013415.1	79.04
LT1P40	Alteristakelama canadensis FWC47 GCA 026013525.1	79.00
LT1P40	Alteristakelama trueperi DSM 7225 GCA 011927635.1	78.90
LT1P40	Alterisphingomonas panacisoli HKS19 GCA 007859635.1	78.89
LT1P40	Alteristakelama leidyi DSM 4733 GCA 011761945.1	78.87
LT1P40	Sphingomonas elodea ATCC 31461 GCA 000226955.2	78.85
LT1P40	Alteristakelama gei ZFGT-11 GCA 004792685.1	78.84
LT1P40	Alteristakelama psychrotolerans Cra20 GCA 002796605.1	78.83
LT1P40	Humisphingomonas gilva ZDH117 GCA 003515075.1	78.83
LT1P40	Parastakelama japonica DSM 22753 GCA 011762085.1	78.82
LT1P40	Alteristakelama pokkalii L3B27 GCA 003096275.1	78.82
LT1P40	**ZT3P38**	78.82
LT1P40	Pseudostakelama cannabina DM2-R-LB4 GCA 021391395.1	78.79
LT1P40	Parasphingomonas hylomeconis CCTCC AB 2013304 GCA 025370105.1	78.78
LT1P40	Novistakelama hankookensis KCTC 22579 GCA 022664465.1	78.76
LT1P40	Novistakelama panni DSM 15761 GCA 022664435.1	78.76
LT1P40	Alteristakelama pituitosa NBRC 102491 GCA 001598435.1	78.75
LT1P40	Sphingomonas phyllosphaerae FA2 GCA 000427645.1	78.72
LT1P40	**PB2P19**	78.71
LT1P40	Sphingomonas adhaesiva DSM 7418 GCA 002374855.1	78.70
LT1P40	Sphingomonas taxi ATCC 55669 GCA 000764535.1	78.70
LT1P40	Parastakelama yantingensis DSM 27244 GCA 014199325.1	78.68
LT1P40	Sphingomonas endophytica DSM 101535 GCA 014199415.1	78.68
LT1P40	Sphingomonas liriopis RP10 GCA 024211255.1	78.68
LT1P40	Parastakelama baiyangensis L-1-4 w-11 GCA 005144715.1	78.66
LT1P40	Parasphingomonas panacis DCY99 GCA 001717955.1	78.65
LT1P40	Sphingomonas ginsenosidivorax KHI67 GCA 007995065.1	78.64
LT1P40	Parastakelama spermidinifaciens 9NM-10 GCA 002351485.1	78.64
LT1P40	Alterisphingomonas pruni NBRC 15498 GCA 001598455.1	78.63
LT1P40	Parasphingomonas aracearum WZY 27 GCA 003345355.1	78.62

LT1P40	Parasphingomonas populi 3 月 7 日 GCA 004208535.1	78.61
LT1P40	Sphingomonas jinjuensis YC6723 GCA 014197105.1	78.60
LT1P40	Alteristakelama xinjiangensis DSM 26736 GCA 014199255.1	78.58
LT1P40	**PB1R3**	78.58
LT1P40	Alterisphingomonas asaccharolytica NBRC 15499 GCA 001598355.1	78.57
LT1P40	Sphingomonas carotinifaciens DSM 27347 GCA 009789535.1	78.57
LT1P40	Sphingomonas metalli CGMCC 1.15330 GCA 014641735.1	78.56
LT1P40	Sphingomonas pseudosanguinis DSM 19512 GCA 014196255.1	78.55
LT1P40	**PB4P5**	78.54
LT1P40	Sphingomonas aquatilis DSM 15581 GCA 014196115.1	78.54
LT1P40	Alteristakelama azotifigens NBRC 15497 GCA 002091475.1	78.53
LT1P40	Sphingomonas melonis DAPP-PG 224 GCA 000379045.1	78.52
LT1P40	Sphingomonas insulae KCTC 12872 GCA 010450875.1	78.52
LT1P40	Sphingomonas ginsenosidimutans KACC 14949 GCA 002374835.1	78.51
LT1P40	Sphingomonas aerolata NW12 GCA 003046295.1	78.51
LT1P40	Sphingomonas parapaucimobilis NBRC 15100 GCA 000787715.1	78.50
LT1P40	Parasphingomonas echinoides ATCC 14820 GCA 000241465.1	78.50
LT1P40	**RT2P30**	78.48
LT1P40	Parasphingomonas glacialis CGMCC 1.8957 GCA 014653575.1	78.48
LT1P40	Parasphingomonas aliaeris DH-S5 GCA 016743815.1	78.48
LT1P40	Novistakelama desiccabilis DSM 16792 GCA 014196135.1	78.48
LT1P40	**RB3P16**	78.45
LT1P40	Sphingomonas oligophenolica CGMCC 1.10181 GCA 039615115.1	78.43
LT1P40	Parasphingomonas alpina DSM 22537 GCA 014490665.1	78.39
LT1P40	Sphingomonas aurantiaca MA101b GCA 003050705.1	78.39
LT1P40	Sphingomonas lycopersici MMSM20 GCA 026130605.1	78.39
LT1P40	Sphingomonas paucimobilis NCTC11030 GCA 900457515.1	78.37
LT1P40	Parasphingomonas psychrolutea CGMCC 1.10106 GCA 014636175.1	78.37
LT1P40	Sphingomonas abaci DSM 15867 GCA 014199625.1	78.36
LT1P40	Sphingomonas yabuuchiae DSM 14562 GCA 014199595.1	78.34
LT1P40	**LB3N6**	78.33
LT1P40	**PB2P12**	78.33
LT1P40	Parasphingomonas qilianensis CGMCC 1.15349 GCA 039614825.1	78.31
LT1P40	Sphingomonas sanguinis NBRC 13937 GCA 001591005.1	78.28
LT1P40	Alterisphingomonas mali NBRC 15500 GCA 001598415.1	78.26
LT1P40	Sphingomonas zeae DSM 100049 GCA 014197135.1	78.25
LT1P40	Sphingomonas rubra CGMCC 1.9113 GCA 900115745.1	78.22
LT1P40	Sphingomonas beigongshangi REN5 GCA 016820445.1	78.21
LT1P40	Sphingomonas olei NM83 B4-11 GCA 004801655.1	78.21
LT1P40	Sphingomonas folli RHCKR7 GCA 019429525.1	78.18
LT1P40	Alterisphingomonas radiodurans S9-5 GCA 020866845.1	78.17
LT1P40	**ZB1N12**	78.17
LT1P40	Sphingomonas dokdonensis DSM 21029 GCA 002197685.1	78.16
LT1P40	Sphingomonas faeni MA-olki GCA 003053745.1	78.13

LT1P40	Yabuuchia colocasiae JCM 31229 GCA 019880585.1	78.12
LT1P40	Sphingomonas hominis HHU CXW GCA 013328205.1	78.10
LT1P40	Sphingomonas citricola RHCKR47 GCA 019429535.1	78.07
LT1P40	Sphingomonas albertensis DOAB 1063 GCA 014358075.1	78.07
LT1P40	Sphingomonas corticis 36D10-4-7 GCA 012035195.1	78.05
LT1P40	**LB2R24**	78.04
LT1P40	Sphingomonas naphthae KACC 18716 GCA 028607085.1	78.04
LT1P40	Yabuuchia cavernae K2R01-6 GCA 003590775.1	78.03
LT1P40	Sphingomonas citri RRHST34 GCA 019429485.1	78.02
LT1P40	Rhizorhabdus crocodyli CCP-7 GCA 004005865.1	78.00
LT1P40	Sphingomonas palmae JS21-1 GCA 900109565.1	77.99
LT1P40	Sphingomonas yunnanensis YIM 3 GCA 019898765.1	77.99
LT1P40	Sphingomonas gellani S6-262 GCA 900110035.1	77.97
LT1P40	Edaphosphingomonas fennica K101 GCA 003034225.1	77.95
LT1P40	Pseudosphingomonas rhizophila KACC 19189 GCA 014396585.1	77.93
LT1P40	Sphingomonas jeddahensis G39 GCA 001981525.1	77.93
LT1P40	Alteriyabuuchia sanxanigenens NX02 GCA 000512205.2	77.92
LT1P40	Pseudostakelama guangdongensis CGMCC 1.12672 GCA 900199185.1	77.92
LT1P40	Parayabuuchia changbaiensis NBRC 104936 GCA 000974765.1	77.92
LT1P40	Rhizorhabdus montanisoli ZX GCA 008274695.1	77.91
LT1P40	Sphingomonas mucosissima DSM 17494 GCA 002197665.1	77.90
LT1P40	Sphingomonas horti MAH-20 GCA 009753715.1	77.88
LT1P40	Edaphosphingomonas haloaromaticamans P3 GCA 001853345.1	77.86
LT1P40	Solisphingomonas oligoaromativorans DSM 102246 GCA 011762195.1	77.82
LT1P40	Pararhizorhabdus prati CGMCC 1.15645 GCA 014643515.1	77.82
LT1P40	Neorhizorhabdus vulcanisoli CECT 8804 GCA 011761305.1	77.81
LT1P40	Sphingomonas changnyeongensis C33 GCA 009913435.1	77.79
LT1P40	Sphingomonas nostoxanthinifaciens AK-PDB1-5 GCA 019930585.1	77.79
LT1P40	Solisphingomonas chungangi MAH-6 GCA 009763135.1	77.78
LT1P40	Parayabuuchia jejuensis DSM 27651 GCA 011927695.1	77.75
LT1P40	Parayabuuchia flavalba ZLT-5 GCA 004796535.1	77.74
LT1P40	Neorhizorhabdus crusticola MIMD3 GCA 003391115.1	77.73
LT1P40	Pseudosphingomonas kaistensis DSM 16846 GCA 011927725.1	77.72
LT1P40	Edaphosphingomonas laterariae LNB2 GCA 900188165.1	77.71
LT1P40	Sphingomonas bisphenolicum AO1 GCA 024349785.1	77.70
LT1P40	Pararhizorhabdus montana W16RD GCA 001956315.1	77.70
LT1P40	Alterirhizorhabdus solaris R4DWN GCA 007785815.1	77.64
LT1P40	Pararhizorhabdus jatrophae S5-249 GCA 900113315.1	77.62
LT1P40	Neorhizorhabdus oleivorans FW-11 GCA 003050615.1	77.62
LT1P40	Sphingomonas lenta 1PNM-20 GCA 002288825.1	77.62
LT1P40	Solisphingomonas quercus XMGL2 GCA 018863195.1	77.61
LT1P40	Pseudosphingomonas sinipercae HDW15C GCA 011302055.1	77.56
LT1P40	Sphingomonas aerophila DSM 100044 GCA 014199305.1	77.55
LT1P40	Sphingomonas ursincola KR-99 GCA 013607875.1	77.52

LT1P40	Pseudosphingomonas jaspsi DSM 18422 GCA 000585415.1	77.49
LT1P40	Alterirhizorhabdus profundi LMO-1 GCA 009739515.1	77.45
LT1P40	Flavisphingomonas formosensis CC-Nfb-2 GCA 009755815.1	77.43
LT1P40	Pseudosphingomonas sabuli sand1-3 GCA 014352855.1	77.39
LT1P40	Pseudosphingomonas astaxanthinifaciens DSM 22298 GCA 000711715.1	77.38
LT1P40	Pseudosphingomonas ginsengisoli KCTC 12630 GCA 003332855.1	77.34
LT1P40	Sphingomonas caseinilyticus NSE70-1 GCA 023516455.1	77.33
LT1P40	Sphingomonas glaciei S8-45 GCA 023380025.1	77.32
LT1P40	Pseudosphingomonas mesophila SYSUP0001 GCA 003499275.1	77.30
LT1P40	Sphingomonas brevis RB56-2 GCA 023516505.1	77.26
LT1P40	Sphingomonas alba SE158 GCA 023516555.1	77.26
LT1P40	Pseudosphingomonas lutea KCTC 23642 GCA 014396785.1	77.23
LT1P40	Pseudosphingomonas ginkgonis HMF7854 GCA 003970925.1	77.20
LT1P40	Sphingomonas xanthus AE3 GCA 007998985.1	77.17
LT1P40	Solisphingomonas morindae NBD5 GCA 023822065.1	77.13
LT1P40	Allosphingosinicella deserti GL-C-18 GCA 003012735.1	77.09
LT1P40	Sphingomonas parva 17J27-24 GCA 004564275.1	77.07
LT1P40	Pseudosphingomonas segetis YJ09 GCA 009720245.1	77.05
LT1P40	Sphingomonas anseongensis RG327 GCA 023516495.1	76.97
LB2R24	**LB3N6**	89.80
LB2R24	Sphingomonas albertensis DOAB 1063 GCA 014358075.1	89.49
LB2R24	Sphingomonas faeni MA-olki GCA 003053745.1	89.11
LB2R24	**ZB1N12**	88.23
LB2R24	Sphingomonas aurantiaca MA101b GCA 003050705.1	87.98
LB2R24	**PB2P12**	87.74
LB2R24	Sphingomonas aerolata NW12 GCA 003046295.1	84.44
LB2R24	Sphingomonas ginsenosidivorax KHI67 GCA 007995065.1	83.59
LB2R24	**PB2P19**	83.18
LB2R24	Sphingomonas liriopis RP10 GCA 024211255.1	80.89
LB2R24	Sphingomonas taxi ATCC 55669 GCA 000764535.1	80.82
LB2R24	**RB3P16**	80.33
LB2R24	Parasphingomonas glacialis CGMCC 1.8957 GCA 014653575.1	80.22
LB2R24	Sphingomonas insulae KCTC 12872 GCA 010450875.1	80.16
LB2R24	Sphingomonas jinjuensis YC6723 GCA 014197105.1	80.06
LB2R24	Parasphingomonas hylomeconis CCTCC AB 2013304 GCA 025370105.1	80.02
LB2R24	Sphingomonas aquatilis DSM 15581 GCA 014196115.1	79.96
LB2R24	Sphingomonas melonis DAPP-PG 224 GCA 000379045.1	79.84
LB2R24	Sphingomonas rubra CGMCC 1.9113 GCA 900115745.1	79.81
LB2R24	Sphingomonas endophytica DSM 101535 GCA 014199415.1	79.77
LB2R24	**GB1N7**	79.65
LB2R24	Sphingomonas yabuuchiae DSM 14562 GCA 014199595.1	79.60
LB2R24	Sphingomonas carotinifaciens DSM 27347 GCA 009789535.1	79.59
LB2R24	Parasphingomonas populi 3 月 7 日 GCA 004208535.1	79.58
LB2R24	Sphingomonas beigongshangi REN5 GCA 016820445.1	79.54

LB2R24	Sphingomonas metalli CGMCC 1.15330 GCA 014641735.1	79.53
LB2R24	Sphingomonas phyllosphaerae FA2 GCA 000427645.1	79.50
LB2R24	Sphingomonas pseudosanguinis DSM 19512 GCA 014196255.1	79.50
LB2R24	Sphingomonas abaci DSM 15867 GCA 014199625.1	79.48
LB2R24	Sphingomonas parapaucimobilis NBRC 15100 GCA 000787715.1	79.42
LB2R24	Parasphingomonas panacis DCY99 GCA 001717955.1	79.41
LB2R24	Sphingomonas adhaesiva DSM 7418 GCA 002374855.1	79.40
LB2R24	Sphingomonas folli RHCKR7 GCA 019429525.1	79.38
LB2R24	Sphingomonas yunnanensis YIM 3 GCA 019898765.1	79.35
LB2R24	Sphingomonas citricola RHCKR47 GCA 019429535.1	79.34
LB2R24	**PB4P5**	79.34
LB2R24	**PB1R3**	79.33
LB2R24	Sphingomonas hominis HHU CXW GCA 013328205.1	79.32
LB2R24	Sphingomonas donggukensis RMG20 GCA 023674425.1	79.30
LB2R24	Sphingomonas sanguinis NBRC 13937 GCA 001591005.1	79.29
LB2R24	**RT2P30**	79.29
LB2R24	Sphingomonas citri RRHST34 GCA 019429485.1	79.26
LB2R24	Alterisphingomonas radiodurans S9-5 GCA 020866845.1	79.23
LB2R24	Pseudostakelama cannabina DM2-R-LB4 GCA 021391395.1	79.22
LB2R24	Novistakelama desiccabilis DSM 16792 GCA 014196135.1	79.22
LB2R24	Alterisphingomonas panacisoli HKS19 GCA 007859635.1	79.20
LB2R24	Sphingomonas zeae DSM 100049 GCA 014197135.1	79.20
LB2R24	Sphingomonas paucimobilis NCTC11030 GCA 900457515.1	79.19
LB2R24	Sphingomonas ginsenosidimutans KACC 14949 GCA 002374835.1	79.18
LB2R24	Parasphingomonas aliaeris DH-S5 GCA 016743815.1	79.18
LB2R24	Parasphingomonas qilianensis CGMCC 1.15349 GCA 039614825.1	79.18
LB2R24	**ZT3P38**	79.17
LB2R24	Sphingomonas oligophenolica CGMCC 1.10181 GCA 039615115.1	79.16
LB2R24	Sphingomonas lycopersici MMSM20 GCA 026130605.1	79.15
LB2R24	Sphingomonas palmae JS21-1 GCA 900109565.1	79.15
LB2R24	Sphingomonas jeddahensis G39 GCA 001981525.1	79.11
LB2R24	Sphingomonas qomolangmaensis S5-59 GCA 024496245.1	79.10
LB2R24	Alteristakelama hengshuiensis WHSC-8 GCA 000935025.1	79.07
LB2R24	Alteristakelama naasensis DSM 100060 GCA 011762145.1	79.07
LB2R24	Sphingomonas corticis 36D10-4-7 GCA 012035195.1	79.05
LB2R24	Parasphingomonas alpina DSM 22537 GCA 014490665.1	78.99
LB2R24	Parasphingomonas psychrolutea CGMCC 1.10106 GCA 014636175.1	78.98
LB2R24	Alteristakelama kyeonggiensis DSM 101806 GCA 014196745.1	78.97
LB2R24	Sphingomonas tagetis MG17 GCA 024211275.1	78.96
LB2R24	Alteristakelama gei ZFGT-11 GCA 004792685.1	78.93
LB2R24	Alteristakelama pokkalii L3B27 GCA 003096275.1	78.91
LB2R24	Parasphingomonas echinoides ATCC 14820 GCA 000241465.1	78.90
LB2R24	Alteristakelama trueperi DSM 7225 GCA 011927635.1	78.90
LB2R24	Parastakelama japonica DSM 22753 GCA 011762085.1	78.86

LB2R24	Sphingomonas olei NM83 B4-11 GCA 004801655.1	78.83
LB2R24	Alteristakelama azotifigens NBRC 15497 GCA 002091475.1	78.81
LB2R24	Humisphingomonas gilva ZDH117 GCA 003515075.1	78.79
LB2R24	Novistakelama hankookensis KCTC 22579 GCA 022664465.1	78.78
LB2R24	Alteristakelama koreensis JSS26 GCA 002797435.1	78.77
LB2R24	Alteristakelama pituitosa NBRC 102491 GCA 001598435.1	78.77
LB2R24	Alteristakelama leidyi DSM 4733 GCA 011761945.1	78.75
LB2R24	Novistakelama panni DSM 15761 GCA 022664435.1	78.75
LB2R24	Parasphingomonas aracearum WZY 27 GCA 003345355.1	78.74
LB2R24	Sphingomonas dokdonensis DSM 21029 GCA 002197685.1	78.73
LB2R24	Parastakelama yantingensis DSM 27244 GCA 014199325.1	78.73
LB2R24	Alterisphingomonas mali NBRC 15500 GCA 001598415.1	78.73
LB2R24	Sphingomonas elodea ATCC 31461 GCA 000226955.2	78.71
LB2R24	Alterisphingomonas pruni NBRC 15498 GCA 001598455.1	78.70
LB2R24	Alterisphingomonas asaccharolytica NBRC 15499 GCA 001598355.1	78.70
LB2R24	Parastakelama spermidinifaciens 9NM-10 GCA 002351485.1	78.69
LB2R24	Pararhizorhabdus prati CGMCC 1.15645 GCA 014643515.1	78.67
LB2R24	Sphingomonas lenta 1PNM-20 GCA 002288825.1	78.61
LB2R24	Alteristakelama psychrotolerans Cra20 GCA 002796605.1	78.57
LB2R24	Sphingomonas gellani S6-262 GCA 900110035.1	78.55
LB2R24	Alteristakelama xinjiangensis DSM 26736 GCA 014199255.1	78.51
LB2R24	Sphingomonas caeni LB-2 GCA 026013415.1	78.49
LB2R24	Sphingomonas mucosissima DSM 17494 GCA 002197665.1	78.49
LB2R24	Parastakelama baiyangensis L-1-4 w-11 GCA 005144715.1	78.42
LB2R24	Alteristakelama soli NBRC 100801 GCA 001591025.1	78.38
LB2R24	Pseudostakelama guangdongensis CGMCC 1.12672 GCA 900199185.1	78.32
LB2R24	Alteristakelama suaedae XS-10 GCA 007833215.1	78.30
LB2R24	Alteristakelama turrisvirgatae MCT13 GCA 001721295.1	78.28
LB2R24	Pararhizorhabdus montana W16RD GCA 001956315.1	78.24
LB2R24	Alteristakelama canadensis FWC47 GCA 026013525.1	78.23
LB2R24	Alteriyabuuchia sanxanigenens NX02 GCA 000512205.2	78.19
LB2R24	Alterirhizorhabdus solaris R4DWN GCA 007785815.1	78.19
LB2R24	Solisphingomonas chungangi MAH-6 GCA 009763135.1	78.18
LB2R24	Rhizorhabdus crocodyli CCP-7 GCA 004005865.1	78.17
LB2R24	Sphingomonas horti MAH-20 GCA 009753715.1	78.15
LB2R24	Sphingomonas naphthae KACC 18716 GCA 028607085.1	78.13
LB2R24	Rhizorhabdus montanisoli ZX GCA 008274695.1	78.13
LB2R24	**LT1P40**	78.12
LB2R24	Yabuuchia cavernae K2R01-6 GCA 003590775.1	78.11
LB2R24	Solisphingomonas oligoaromativorans DSM 102246 GCA 011762195.1	78.05
LB2R24	Parayabuuchia changbaiensis NBRC 104936 GCA 000974765.1	78.05
LB2R24	Sphingomonas aerophila DSM 100044 GCA 014199305.1	78.04
LB2R24	Sphingomonas nostoxanthinifaciens AK-PDB1-5 GCA 019930585.1	78.03
LB2R24	Solisphingomonas quercus XMGL2 GCA 018863195.1	78.02

LB2R24	Edaphosphingomonas laterariae LNB2 GCA 900188165.1	77.96
LB2R24	Parayabuuchia flavalba ZLT-5 GCA 004796535.1	77.95
LB2R24	Parayabuuchia jejuensis DSM 27651 GCA 011927695.1	77.94
LB2R24	Pseudosphingomonas astaxanthinifaciens DSM 22298 GCA 000711715.1	77.88
LB2R24	Pararhizorhabdus jatrophae S5-249 GCA 900113315.1	77.86
LB2R24	Edaphosphingomonas haloaromaticamans P3 GCA 001853345.1	77.84
LB2R24	Sphingomonas changnyeongensis C33 GCA 009913435.1	77.84
LB2R24	Yabuuchia colocasiae JCM 31229 GCA 019880585.1	77.83
LB2R24	Sphingomonas parva 17J27-24 GCA 004564275.1	77.83
LB2R24	Neorhizorhabdus vulcanisoli CECT 8804 GCA 011761305.1	77.82
LB2R24	Edaphosphingomonas fennica K101 GCA 003034225.1	77.77
LB2R24	Pseudosphingomonas rhizophila KACC 19189 GCA 014396585.1	77.77
LB2R24	Pseudosphingomonas ginsengisoli KCTC 12630 GCA 003332855.1	77.76
LB2R24	Pseudosphingomonas kaistensis DSM 16846 GCA 011927725.1	77.76
LB2R24	Alterirhizorhabdus profundi LMO-1 GCA 009739515.1	77.69
LB2R24	Pseudosphingomonas ginkgonis HMF7854 GCA 003970925.1	77.66
LB2R24	Flavisphingomonas formosensis CC-Nfb-2 GCA 009755815.1	77.60
LB2R24	Pseudosphingomonas jaspsi DSM 18422 GCA 000585415.1	77.56
LB2R24	Sphingomonas ursincola KR-99 GCA 013607875.1	77.55
LB2R24	Sphingomonas glaciei S8-45 GCA 023380025.1	77.52
LB2R24	Pseudosphingomonas sabuli sand1-3 GCA 014352855.1	77.45
LB2R24	Sphingomonas bisphenolicum AO1 GCA 024349785.1	77.44
LB2R24	Sphingomonas brevis RB56-2 GCA 023516505.1	77.41
LB2R24	Neorhizorhabdus crusticola MIMD3 GCA 003391115.1	77.38
LB2R24	Neorhizorhabdus oleivorans FW-11 GCA 003050615.1	77.37
LB2R24	Pseudosphingomonas lutea KCTC 23642 GCA 014396785.1	77.36
LB2R24	Sphingomonas anseongensis RG327 GCA 023516495.1	77.35
LB2R24	Pseudosphingomonas mesophila SYSUP0001 GCA 003499275.1	77.33
LB2R24	Pseudosphingomonas piscis HDW15B GCA 011300455.1	77.32
LB2R24	Pseudosphingomonas arenae SYSU D00720 GCA 016924655.1	77.30
LB2R24	Sphingomonas telluris SM33 GCA 022568775.1	77.29
LB2R24	Solisphingomonas morindae NBD5 GCA 023822065.1	77.28
LB2R24	Pseudosphingomonas sinipercae HDW15C GCA 011302055.1	77.27
LB2R24	Sphingomonas alba SE158 GCA 023516555.1	77.25
LB2R24	Allosphingosinicella deserti GL-C-18 GCA 003012735.1	77.19
LB2R24	Pseudosphingomonas segetis YJ09 GCA 009720245.1	77.18
LB2R24	Sphingomonas xanthus AE3 GCA 007998985.1	77.12
LB3N6	Sphingomonas albertensis DOAB 1063 GCA 014358075.1	91.26
LB3N6	**LB2R24**	89.81
LB3N6	**ZB1N12**	89.14
LB3N6	Sphingomonas faeni MA-olki GCA 003053745.1	88.16
LB3N6	Sphingomonas aurantiaca MA101b GCA 003050705.1	87.91
LB3N6	**PB2P12**	87.65
LB3N6	Sphingomonas aerolata NW12 GCA 003046295.1	84.44

LB3N6	Sphingomonas ginsenosidivorax KHI67 GCA 007995065.1	83.58
LB3N6	**PB2P19**	83.10
LB3N6	Sphingomonas liriopis RP10 GCA 024211255.1	80.91
LB3N6	Sphingomonas taxi ATCC 55669 GCA 000764535.1	80.89
LB3N6	**RB3P16**	80.30
LB3N6	Sphingomonas melonis DAPP-PG 224 GCA 000379045.1	80.26
LB3N6	Parasphingomonas glacialis CGMCC 1.8957 GCA 014653575.1	80.23
LB3N6	Parasphingomonas hylomeconis CCTCC AB 2013304 GCA 025370105.1	80.21
LB3N6	Sphingomonas jinjuensis YC6723 GCA 014197105.1	80.13
LB3N6	Sphingomonas aquatilis DSM 15581 GCA 014196115.1	80.13
LB3N6	Sphingomonas insulae KCTC 12872 GCA 010450875.1	80.12
LB3N6	Sphingomonas rubra CGMCC 1.9113 GCA 900115745.1	80.06
LB3N6	Sphingomonas endophytica DSM 101535 GCA 014199415.1	79.96
LB3N6	Sphingomonas metalli CGMCC 1.15330 GCA 014641735.1	79.79
LB3N6	Sphingomonas phyllosphaerae FA2 GCA 000427645.1	79.78
LB3N6	**GB1N7**	79.74
LB3N6	Sphingomonas pseudosanguinis DSM 19512 GCA 014196255.1	79.73
LB3N6	Sphingomonas carotinifaciens DSM 27347 GCA 009789535.1	79.68
LB3N6	Parasphingomonas populi 3 月 7 日 GCA 004208535.1	79.65
LB3N6	Sphingomonas beigongshangi REN5 GCA 016820445.1	79.63
LB3N6	Sphingomonas donggukensis RMG20 GCA 023674425.1	79.57
LB3N6	**RT2P30**	79.53
LB3N6	Sphingomonas hominis HHU CXW GCA 013328205.1	79.51
LB3N6	Parasphingomonas panacis DCY99 GCA 001717955.1	79.49
LB3N6	Sphingomonas citricola RHCKR47 GCA 019429535.1	79.47
LB3N6	**PB4P5**	79.47
LB3N6	Sphingomonas qomolangmaensis S5-59 GCA 024496245.1	79.47
LB3N6	Sphingomonas abaci DSM 15867 GCA 014199625.1	79.44
LB3N6	Parasphingomonas qilianensis CGMCC 1.15349 GCA 039614825.1	79.44
LB3N6	Sphingomonas citri RRHST34 GCA 019429485.1	79.44
LB3N6	Sphingomonas adhaesiva DSM 7418 GCA 002374855.1	79.43
LB3N6	Parasphingomonas aliaeris DH-S5 GCA 016743815.1	79.42
LB3N6	Sphingomonas folli RHCKR7 GCA 019429525.1	79.40
LB3N6	Sphingomonas ginsenosidimutans KACC 14949 GCA 002374835.1	79.39
LB3N6	Sphingomonas sanguinis NBRC 13937 GCA 001591005.1	79.39
LB3N6	Alterisphingomonas panacisoli HKS19 GCA 007859635.1	79.37
LB3N6	Sphingomonas parapaucimobilis NBRC 15100 GCA 000787715.1	79.31
LB3N6	Alterisphingomonas radiodurans S9-5 GCA 020866845.1	79.29
LB3N6	Sphingomonas yunnanensis YIM 3 GCA 019898765.1	79.29
LB3N6	Pseudostakelama cannabina DM2-R-LB4 GCA 021391395.1	79.28
LB3N6	Sphingomonas yabuuchiae DSM 14562 GCA 014199595.1	79.27
LB3N6	**PB1R3**	79.26
LB3N6	Sphingomonas paucimobilis NCTC11030 GCA 900457515.1	79.23
LB3N6	Sphingomonas palmae JS21-1 GCA 900109565.1	79.22

LB3N6	Sphingomonas lycopersici MMSM20 GCA 026130605.1	79.21
LB3N6	**ZT3P38**	79.21
LB3N6	Alteristakelama naasensis DSM 100060 GCA 011762145.1	79.19
LB3N6	Sphingomonas jeddahensis G39 GCA 001981525.1	79.19
LB3N6	Parasphingomonas echinoides ATCC 14820 GCA 000241465.1	79.18
LB3N6	Sphingomonas tagetis MG17 GCA 024211275.1	79.08
LB3N6	Parasphingomonas alpina DSM 22537 GCA 014490665.1	79.07
LB3N6	Sphingomonas oligophenolica CGMCC 1.10181 GCA 039615115.1	79.06
LB3N6	Parastakelama yantingensis DSM 27244 GCA 014199325.1	79.05
LB3N6	Sphingomonas zeae DSM 100049 GCA 014197135.1	79.04
LB3N6	Parastakelama japonica DSM 22753 GCA 011762085.1	79.04
LB3N6	Alterisphingomonas asaccharolytica NBRC 15499 GCA 001598355.1	79.03
LB3N6	Alteristakelama pituitosa NBRC 102491 GCA 001598435.1	79.03
LB3N6	Alteristakelama kyeonggiensis DSM 101806 GCA 014196745.1	79.03
LB3N6	Alteristakelama gei ZFGT-11 GCA 004792685.1	79.02
LB3N6	Parasphingomonas psychrolutea CGMCC 1.10106 GCA 014636175.1	79.01
LB3N6	Sphingomonas corticis 36D10-4-7 GCA 012035195.1	79.00
LB3N6	Novistakelama desiccabilis DSM 16792 GCA 014196135.1	78.98
LB3N6	Parasphingomonas aracearum WZY 27 GCA 003345355.1	78.98
LB3N6	Sphingomonas elodea ATCC 31461 GCA 000226955.2	78.96
LB3N6	Alteristakelama hengshuiensis WHSC-8 GCA 000935025.1	78.94
LB3N6	Alteristakelama azotifigens NBRC 15497 GCA 002091475.1	78.94
LB3N6	Humisphingomonas gilva ZDH117 GCA 003515075.1	78.92
LB3N6	Sphingomonas dokdonensis DSM 21029 GCA 002197685.1	78.91
LB3N6	Alteristakelama trueperi DSM 7225 GCA 011927635.1	78.90
LB3N6	Pararhizorhabdus prati CGMCC 1.15645 GCA 014643515.1	78.89
LB3N6	Sphingomonas olei NM83 B4-11 GCA 004801655.1	78.88
LB3N6	Sphingomonas gellani S6-262 GCA 900110035.1	78.88
LB3N6	Novistakelama panni DSM 15761 GCA 022664435.1	78.88
LB3N6	Alterisphingomonas pruni NBRC 15498 GCA 001598455.1	78.85
LB3N6	Novistakelama hankookensis KCTC 22579 GCA 022664465.1	78.85
LB3N6	Alterisphingomonas mali NBRC 15500 GCA 001598415.1	78.85
LB3N6	Sphingomonas caeni LB-2 GCA 026013415.1	78.84
LB3N6	Alteristakelama pokkalii L3B27 GCA 003096275.1	78.82
LB3N6	Alteristakelama leidyi DSM 4733 GCA 011761945.1	78.82
LB3N6	Parastakelama spermidinifaciens 9NM-10 GCA 002351485.1	78.81
LB3N6	Alteristakelama psychrotolerans Cra20 GCA 002796605.1	78.74
LB3N6	Alteristakelama suaedae XS-10 GCA 007833215.1	78.73
LB3N6	Parastakelama baiyangensis L-1-4 w-11 GCA 005144715.1	78.71
LB3N6	Alteristakelama soli NBRC 100801 GCA 001591025.1	78.69
LB3N6	Alteristakelama xinjiangensis DSM 26736 GCA 014199255.1	78.66
LB3N6	Alteristakelama koreensis JSS26 GCA 002797435.1	78.66
LB3N6	Sphingomonas lenta 1PNM-20 GCA 002288825.1	78.56
LB3N6	Sphingomonas mucosissima DSM 17494 GCA 002197665.1	78.53

LB3N6	**LT1P40**	78.47
LB3N6	Pseudostakelama guangdongensis CGMCC 1.12672 GCA 900199185.1	78.45
LB3N6	Alterirhizorhabdus solaris R4DWN GCA 007785815.1	78.40
LB3N6	Rhizorhabdus crocodyli CCP-7 GCA 004005865.1	78.38
LB3N6	Sphingomonas naphthae KACC 18716 GCA 028607085.1	78.38
LB3N6	Pararhizorhabdus montana W16RD GCA 001956315.1	78.38
LB3N6	Solisphingomonas oligoaromativorans DSM 102246 GCA 011762195.1	78.36
LB3N6	Sphingomonas nostoxanthinifaciens AK-PDB1-5 GCA 019930585.1	78.34
LB3N6	Alteriyabuuchia sanxanigenens NX02 GCA 000512205.2	78.33
LB3N6	Alteristakelama turrisvirgatae MCT13 GCA 001721295.1	78.33
LB3N6	Sphingomonas aerophila DSM 100044 GCA 014199305.1	78.30
LB3N6	Sphingomonas horti MAH-20 GCA 009753715.1	78.30
LB3N6	Yabuuchia cavernae K2R01-6 GCA 003590775.1	78.30
LB3N6	Solisphingomonas chungangi MAH-6 GCA 009763135.1	78.30
LB3N6	Rhizorhabdus montanisoli ZX GCA 008274695.1	78.28
LB3N6	Sphingomonas parva 17J27-24 GCA 004564275.1	78.27
LB3N6	Parayabuuchia changbaiensis NBRC 104936 GCA 000974765.1	78.25
LB3N6	Pararhizorhabdus jatrophae S5-249 GCA 900113315.1	78.20
LB3N6	Alteristakelama canadensis FWC47 GCA 026013525.1	78.20
LB3N6	Sphingomonas changnyeongensis C33 GCA 009913435.1	78.13
LB3N6	Solisphingomonas quercus XMGL2 GCA 018863195.1	78.07
LB3N6	Pseudosphingomonas astaxanthinifaciens DSM 22298 GCA 000711715.1	78.01
LB3N6	Neorhizorhabdus crusticola MIMD3 GCA 003391115.1	77.99
LB3N6	Yabuuchia colocasiae JCM 31229 GCA 019880585.1	77.98
LB3N6	Parayabuuchia flavalba ZLT-5 GCA 004796535.1	77.96
LB3N6	Edaphosphingomonas fennica K101 GCA 003034225.1	77.94
LB3N6	Neorhizorhabdus vulcanisoli CECT 8804 GCA 011761305.1	77.88
LB3N6	Pseudosphingomonas kaistensis DSM 16846 GCA 011927725.1	77.86
LB3N6	Pseudosphingomonas lutea KCTC 23642 GCA 014396785.1	77.85
LB3N6	Pseudosphingomonas rhizophila KACC 19189 GCA 014396585.1	77.84
LB3N6	Sphingomonas ursincola KR-99 GCA 013607875.1	77.84
LB3N6	Edaphosphingomonas haloaromaticamans P3 GCA 001853345.1	77.83
LB3N6	Parayabuuchia jejuensis DSM 27651 GCA 011927695.1	77.83
LB3N6	Edaphosphingomonas laterariae LNB2 GCA 900188165.1	77.83
LB3N6	Neorhizorhabdus oleivorans FW-11 GCA 003050615.1	77.81
LB3N6	Pseudosphingomonas ginsengisoli KCTC 12630 GCA 003332855.1	77.80
LB3N6	Alterirhizorhabdus profundi LMO-1 GCA 009739515.1	77.78
LB3N6	Pseudosphingomonas mesophila SYSUP0001 GCA 003499275.1	77.77
LB3N6	Pseudosphingomonas segetis YJ09 GCA 009720245.1	77.74
LB3N6	Pseudosphingomonas jaspsi DSM 18422 GCA 000585415.1	77.73
LB3N6	Pseudosphingomonas sinipercae HDW15C GCA 011302055.1	77.71
LB3N6	Pseudosphingomonas ginkgonis HMF7854 GCA 003970925.1	77.69
LB3N6	Flavisphingomonas formosensis CC-Nfb-2 GCA 009755815.1	77.67
LB3N6	Pseudosphingomonas sabuli sand1-3 GCA 014352855.1	77.63

LB3N6	Sphingomonas alba SE158 GCA 023516555.1	77.61
LB3N6	Pseudosphingomonas arenae SYSU D00720 GCA 016924655.1	77.61
LB3N6	Solisphingomonas morindae NBD5 GCA 023822065.1	77.61
LB3N6	Sphingomonas glaciei S8-45 GCA 023380025.1	77.60
LB3N6	Sphingomonas bisphenolicum AO1 GCA 024349785.1	77.59
LB3N6	Pseudosphingomonas piscis HDW15B GCA 011300455.1	77.58
LB3N6	Sphingomonas anseongensis RG327 GCA 023516495.1	77.54
LB3N6	Sphingomonas brevis RB56-2 GCA 023516505.1	77.52
LB3N6	Sphingomonas telluris SM33 GCA 022568775.1	77.40
LB3N6	Allosphingosinicella deserti GL-C-18 GCA 003012735.1	77.28
LB3N6	Sphingomonas xanthus AE3 GCA 007998985.1	77.00
RB3P16	Parasphingomonas glacialis CGMCC 1.8957 GCA 014653575.1	88.32
RB3P16	Parasphingomonas echinoides ATCC 14820 GCA 000241465.1	83.56
RB3P16	Parasphingomonas psychrolutea CGMCC 1.10106 GCA 014636175.1	82.99
RB3P16	Parasphingomonas hylomeconis CCTCC AB 2013304 GCA 025370105.1	81.74
RB3P16	Sphingomonas ginsenosidivorax KHI67 GCA 007995065.1	81.41
RB3P16	Parasphingomonas populi 3 月 7 日 GCA 004208535.1	81.38
RB3P16	**RT2P30**	81.13
RB3P16	Parasphingomonas panacis DCY99 GCA 001717955.1	81.12
RB3P16	Sphingomonas aurantiaca MA101b GCA 003050705.1	81.06
RB3P16	Sphingomonas oligophenolica CGMCC 1.10181 GCA 039615115.1	81.01
RB3P16	**PB4P5**	80.91
RB3P16	Sphingomonas aerolata NW12 GCA 003046295.1	80.80
RB3P16	**ZT3P38**	80.73
RB3P16	**PB2P19**	80.71
RB3P16	**GB1N7**	80.69
RB3P16	Parasphingomonas qilianensis CGMCC 1.15349 GCA 039614825.1	80.62
RB3P16	Sphingomonas taxi ATCC 55669 GCA 000764535.1	80.58
RB3P16	Sphingomonas liriopis RP10 GCA 024211255.1	80.52
RB3P16	Sphingomonas faeni MA-olki GCA 003053745.1	80.41
RB3P16	Parasphingomonas alpina DSM 22537 GCA 014490665.1	80.36
RB3P16	Sphingomonas albertensis DOAB 1063 GCA 014358075.1	80.32
RB3P16	**ZB1N12**	80.29
RB3P16	**LB3N6**	80.25
RB3P16	**LB2R24**	80.22
RB3P16	Sphingomonas qomolangmaensis S5-59 GCA 024496245.1	80.18
RB3P16	Alteristakelama naasensis DSM 100060 GCA 011762145.1	80.09
RB3P16	**PB2P12**	80.03
RB3P16	Pseudostakelama cannabina DM2-R-LB4 GCA 021391395.1	80.02
RB3P16	Parasphingomonas aliaeris DH-S5 GCA 016743815.1	80.00
RB3P16	Sphingomonas lycopersici MMSM20 GCA 026130605.1	79.99
RB3P16	Sphingomonas melonis DAPP-PG 224 GCA 000379045.1	79.92
RB3P16	Alteristakelama hengshuiensis WHSC-8 GCA 000935025.1	79.92
RB3P16	Sphingomonas jinjuensis YC6723 GCA 014197105.1	79.89

RB3P16	Sphingomonas endophytica DSM 101535 GCA 014199415.1	79.87
RB3P16	Alterisphingomonas panacisoli HKS19 GCA 007859635.1	79.87
RB3P16	Sphingomonas aquatilis DSM 15581 GCA 014196115.1	79.81
RB3P16	Alterisphingomonas mali NBRC 15500 GCA 001598415.1	79.77
RB3P16	Alterisphingomonas asaccharolytica NBRC 15499 GCA 001598355.1	79.75
RB3P16	Alteristakelama trueperi DSM 7225 GCA 011927635.1	79.74
RB3P16	Sphingomonas donggukensis RMG20 GCA 023674425.1	79.74
RB3P16	Sphingomonas tagetis MG17 GCA 024211275.1	79.72
RB3P16	Alteristakelama gei ZFGT-11 GCA 004792685.1	79.71
RB3P16	Sphingomonas insulae KCTC 12872 GCA 010450875.1	79.70
RB3P16	Sphingomonas elodea ATCC 31461 GCA 000226955.2	79.70
RB3P16	Alterisphingomonas pruni NBRC 15498 GCA 001598455.1	79.69
RB3P16	Alteristakelama pituitosa NBRC 102491 GCA 001598435.1	79.65
RB3P16	Sphingomonas folli RHCKR7 GCA 019429525.1	79.63
RB3P16	Sphingomonas phyllosphaerae FA2 GCA 000427645.1	79.61
RB3P16	Sphingomonas citricola RHCKR47 GCA 019429535.1	79.59
RB3P16	Sphingomonas caeni LB-2 GCA 026013415.1	79.57
RB3P16	Parastakelama japonica DSM 22753 GCA 011762085.1	79.56
RB3P16	Alteristakelama azotifigens NBRC 15497 GCA 002091475.1	79.55
RB3P16	Sphingomonas ginsenosidimutans KACC 14949 GCA 002374835.1	79.53
RB3P16	Sphingomonas palmae JS21-1 GCA 900109565.1	79.51
RB3P16	Sphingomonas citri RRHST34 GCA 019429485.1	79.51
RB3P16	Sphingomonas rubra CGMCC 1.9113 GCA 900115745.1	79.50
RB3P16	Sphingomonas hominis HHU CXW GCA 013328205.1	79.49
RB3P16	Parasphingomonas aracearum WZY 27 GCA 003345355.1	79.49
RB3P16	Parastakelama baiyangensis L-1-4 w-11 GCA 005144715.1	79.48
RB3P16	Sphingomonas yunnanensis YIM 3 GCA 019898765.1	79.47
RB3P16	Alteristakelama psychrotolerans Cra20 GCA 002796605.1	79.44
RB3P16	Sphingomonas beigongshangi REN5 GCA 016820445.1	79.43
RB3P16	Alterisphingomonas radiodurans S9-5 GCA 020866845.1	79.43
RB3P16	Sphingomonas abaci DSM 15867 GCA 014199625.1	79.43
RB3P16	Alteristakelama leidyi DSM 4733 GCA 011761945.1	79.39
RB3P16	Sphingomonas adhaesiva DSM 7418 GCA 002374855.1	79.39
RB3P16	Alteristakelama kyeonggiensis DSM 101806 GCA 014196745.1	79.37
RB3P16	Sphingomonas metalli CGMCC 1.15330 GCA 014641735.1	79.34
RB3P16	Sphingomonas jeddahensis G39 GCA 001981525.1	79.34
RB3P16	Humisphingomonas gilva ZDH117 GCA 003515075.1	79.33
RB3P16	Alteristakelama pokkalii L3B27 GCA 003096275.1	79.31
RB3P16	Sphingomonas pseudosanguinis DSM 19512 GCA 014196255.1	79.27
RB3P16	Parastakelama spermidinifaciens 9NM-10 GCA 002351485.1	79.26
RB3P16	Sphingomonas parapaucimobilis NBRC 15100 GCA 000787715.1	79.25
RB3P16	Alteristakelama koreensis JSS26 GCA 002797435.1	79.23
RB3P16	**PB1R3**	79.20
RB3P16	Sphingomonas carotinifaciens DSM 27347 GCA 009789535.1	79.17

RB3P16	Sphingomonas dokdonensis DSM 21029 GCA 002197685.1	79.16
RB3P16	Novistakelama desiccabilis DSM 16792 GCA 014196135.1	79.16
RB3P16	Sphingomonas yabuuchiae DSM 14562 GCA 014199595.1	79.12
RB3P16	Alteristakelama soli NBRC 100801 GCA 001591025.1	79.08
RB3P16	Sphingomonas paucimobilis NCTC11030 GCA 900457515.1	79.08
RB3P16	Parastakelama yantingensis DSM 27244 GCA 014199325.1	79.01
RB3P16	Sphingomonas olei NM83 B4-11 GCA 004801655.1	79.00
RB3P16	Novistakelama panni DSM 15761 GCA 022664435.1	78.98
RB3P16	Sphingomonas sanguinis NBRC 13937 GCA 001591005.1	78.98
RB3P16	Alteristakelama suaedae XS-10 GCA 007833215.1	78.98
RB3P16	Sphingomonas corticis 36D10-4-7 GCA 012035195.1	78.92
RB3P16	Sphingomonas zeae DSM 100049 GCA 014197135.1	78.90
RB3P16	Novistakelama hankookensis KCTC 22579 GCA 022664465.1	78.86
RB3P16	Sphingomonas mucosissima DSM 17494 GCA 002197665.1	78.83
RB3P16	Alteriyabuuchia sanxanigenens NX02 GCA 000512205.2	78.82
RB3P16	Sphingomonas naphthae KACC 18716 GCA 028607085.1	78.82
RB3P16	Sphingomonas nostoxanthinifaciens AK-PDB1-5 GCA 019930585.1	78.81
RB3P16	Alteristakelama canadensis FWC47 GCA 026013525.1	78.81
RB3P16	Alteristakelama xinjiangensis DSM 26736 GCA 014199255.1	78.80
RB3P16	Solisphingomonas chungangi MAH-6 GCA 009763135.1	78.78
RB3P16	Solisphingomonas oligoaromativorans DSM 102246 GCA 011762195.1	78.77
RB3P16	Pseudostakelama guangdongensis CGMCC 1.12672 GCA 900199185.1	78.77
RB3P16	Yabuuchia cavernae K2R01-6 GCA 003590775.1	78.74
RB3P16	Alteristakelama turrisvirgatae MCT13 GCA 001721295.1	78.71
RB3P16	Sphingomonas lenta 1PNM-20 GCA 002288825.1	78.59
RB3P16	Neorhizorhabdus vulcanisoli CECT 8804 GCA 011761305.1	78.54
RB3P16	Rhizorhabdus montanisoli ZX GCA 008274695.1	78.51
RB3P16	Parayabuuchia changbaiensis NBRC 104936 GCA 000974765.1	78.51
RB3P16	Alterirhizorhabdus solaris R4DWN GCA 007785815.1	78.49
RB3P16	**LT1P40**	78.44
RB3P16	Sphingomonas changnyeongensis C33 GCA 009913435.1	78.42
RB3P16	Rhizorhabdus crocodyli CCP-7 GCA 004005865.1	78.40
RB3P16	Alterirhizorhabdus profundi LMO-1 GCA 009739515.1	78.37
RB3P16	Pararhizorhabdus jatrophae S5-249 GCA 900113315.1	78.36
RB3P16	Yabuuchia colocasiae JCM 31229 GCA 019880585.1	78.33
RB3P16	Pararhizorhabdus prati CGMCC 1.15645 GCA 014643515.1	78.32
RB3P16	Solisphingomonas quercus XMGL2 GCA 018863195.1	78.31
RB3P16	Sphingomonas gellani S6-262 GCA 900110035.1	78.30
RB3P16	Sphingomonas ursincola KR-99 GCA 013607875.1	78.30
RB3P16	Pseudosphingomonas astaxanthinifaciens DSM 22298 GCA 000711715.1	78.29
RB3P16	Sphingomonas horti MAH-20 GCA 009753715.1	78.27
RB3P16	Neorhizorhabdus oleivorans FW-11 GCA 003050615.1	78.25
RB3P16	Parayabuuchia flavalba ZLT-5 GCA 004796535.1	78.22
RB3P16	Edaphosphingomonas laterariae LNB2 GCA 900188165.1	78.20

RB3P16	Edaphosphingomonas fennica K101 GCA 003034225.1	78.18
RB3P16	Edaphosphingomonas haloaromaticamans P3 GCA 001853345.1	78.18
RB3P16	Pseudosphingomonas ginsengisoli KCTC 12630 GCA 003332855.1	78.17
RB3P16	Neorhizorhabdus crusticola MIMD3 GCA 003391115.1	78.16
RB3P16	Sphingomonas parva 17J27-24 GCA 004564275.1	78.13
RB3P16	Pseudosphingomonas kaistensis DSM 16846 GCA 011927725.1	78.06
RB3P16	Solisphingomonas morindae NBD5 GCA 023822065.1	78.03
RB3P16	Sphingomonas glaciei S8-45 GCA 023380025.1	78.02
RB3P16	Pseudosphingomonas mesophila SYSUP0001 GCA 003499275.1	78.00
RB3P16	Sphingomonas aerophila DSM 100044 GCA 014199305.1	77.99
RB3P16	Pseudosphingomonas sinipercae HDW15C GCA 011302055.1	77.98
RB3P16	Flavisphingomonas formosensis CC-Nfb-2 GCA 009755815.1	77.95
RB3P16	Pseudosphingomonas ginkgonis HMF7854 GCA 003970925.1	77.94
RB3P16	Pararhizorhabdus montana W16RD GCA 001956315.1	77.94
RB3P16	Pseudosphingomonas jaspsi DSM 18422 GCA 000585415.1	77.90
RB3P16	Allosphingosinicella deserti GL-C-18 GCA 003012735.1	77.84
RB3P16	Sphingomonas bisphenolicum AO1 GCA 024349785.1	77.82
RB3P16	Pseudosphingomonas segetis YJ09 GCA 009720245.1	77.79
RB3P16	Parayabuuchia jejuensis DSM 27651 GCA 011927695.1	77.77
RB3P16	Pseudosphingomonas rhizophila KACC 19189 GCA 014396585.1	77.77
RB3P16	Sphingomonas caseinilyticus NSE70-1 GCA 023516455.1	77.72
RB3P16	Pseudosphingomonas lutea KCTC 23642 GCA 014396785.1	77.67
RB3P16	Sphingomonas telluris SM33 GCA 022568775.1	77.67
RB3P16	Pseudosphingomonas sabuli sand1-3 GCA 014352855.1	77.67
RB3P16	Sphingomonas cremea G124 GCA 021502585.1	77.65
RB3P16	Pseudosphingomonas arenae SYSU D00720 GCA 016924655.1	77.63
RB3P16	Pseudosphingomonas piscis HDW15B GCA 011300455.1	77.61
RB3P16	Sphingomonas sediminicola KACC 15039 GCA 014489515.1	77.59
RB3P16	Sphingomonas alba SE158 GCA 023516555.1	77.57
RB3P16	Sphingomonas brevis RB56-2 GCA 023516505.1	77.43
RB3P16	Sphingomonas hankyongi SE220 GCA 023516575.1	77.33
RB3P16	Sphingomonas anseongensis RG327 GCA 023516495.1	77.30
RB3P16	Sphingomonas xanthus AE3 GCA 007998985.1	77.22
RT2P30	**RB3P16**	81.12
RT2P30	Sphingomonas oligophenolica CGMCC 1.10181 GCA 039615115.1	81.11
RT2P30	Parasphingomonas hylomeconis CCTCC AB 2013304 GCA 025370105.1	81.04
RT2P30	Parasphingomonas glacialis CGMCC 1.8957 GCA 014653575.1	80.97
RT2P30	**ZT3P38**	80.83
RT2P30	Parasphingomonas populi 3 月 7 日 GCA 004208535.1	80.60
RT2P30	Sphingomonas ginsenosidivorax KHI67 GCA 007995065.1	80.59
RT2P30	Parasphingomonas alpina DSM 22537 GCA 014490665.1	80.54
RT2P30	**PB4P5**	80.53
RT2P30	Parasphingomonas panacis DCY99 GCA 001717955.1	80.42
RT2P30	Parasphingomonas echinoides ATCC 14820 GCA 000241465.1	80.42

RT2P30	**GB1N7**	80.40
RT2P30	Sphingomonas taxi ATCC 55669 GCA 000764535.1	80.22
RT2P30	Parasphingomonas psychrolutea CGMCC 1.10106 GCA 014636175.1	80.21
RT2P30	Alterisphingomonas panacisoli HKS19 GCA 007859635.1	80.12
RT2P30	Alterisphingomonas mali NBRC 15500 GCA 001598415.1	80.04
RT2P30	Alterisphingomonas pruni NBRC 15498 GCA 001598455.1	80.02
RT2P30	Alterisphingomonas asaccharolytica NBRC 15499 GCA 001598355.1	80.01
RT2P30	Parasphingomonas qilianensis CGMCC 1.15349 GCA 039614825.1	80.01
RT2P30	**PB2P19**	79.99
RT2P30	Sphingomonas liriopis RP10 GCA 024211255.1	79.98
RT2P30	Sphingomonas aerolata NW12 GCA 003046295.1	79.97
RT2P30	Alteristakelama naasensis DSM 100060 GCA 011762145.1	79.96
RT2P30	Sphingomonas tagetis MG17 GCA 024211275.1	79.96
RT2P30	Sphingomonas lycopersici MMSM20 GCA 026130605.1	79.83
RT2P30	Sphingomonas qomolangmaensis S5-59 GCA 024496245.1	79.80
RT2P30	Pseudostakelama cannabina DM2-R-LB4 GCA 021391395.1	79.77
RT2P30	Humisphingomonas gilva ZDH117 GCA 003515075.1	79.75
RT2P30	Sphingomonas metalli CGMCC 1.15330 GCA 014641735.1	79.74
RT2P30	Sphingomonas aurantiaca MA101b GCA 003050705.1	79.73
RT2P30	Sphingomonas endophytica DSM 101535 GCA 014199415.1	79.71
RT2P30	Sphingomonas abaci DSM 15867 GCA 014199625.1	79.66
RT2P30	Sphingomonas folli RHCKR7 GCA 019429525.1	79.66
RT2P30	Alteristakelama leidyi DSM 4733 GCA 011761945.1	79.66
RT2P30	Alteristakelama gei ZFGT-11 GCA 004792685.1	79.65
RT2P30	Sphingomonas jinjuensis YC6723 GCA 014197105.1	79.65
RT2P30	Alteristakelama kyeonggiensis DSM 101806 GCA 014196745.1	79.63
RT2P30	Parastakelama japonica DSM 22753 GCA 011762085.1	79.63
RT2P30	Alteristakelama trueperi DSM 7225 GCA 011927635.1	79.61
RT2P30	Sphingomonas caeni LB-2 GCA 026013415.1	79.59
RT2P30	Sphingomonas melonis DAPP-PG 224 GCA 000379045.1	79.56
RT2P30	Alteristakelama psychrotolerans Cra20 GCA 002796605.1	79.56
RT2P30	Alteristakelama hengshuiensis WHSC-8 GCA 000935025.1	79.56
RT2P30	Sphingomonas insulae KCTC 12872 GCA 010450875.1	79.56
RT2P30	Parastakelama baiyangensis L-1-4 w-11 GCA 005144715.1	79.51
RT2P30	Sphingomonas aquatilis DSM 15581 GCA 014196115.1	79.49
RT2P30	Alteristakelama azotifigens NBRC 15497 GCA 002091475.1	79.48
RT2P30	Alteristakelama pokkalii L3B27 GCA 003096275.1	79.47
RT2P30	Sphingomonas adhaesiva DSM 7418 GCA 002374855.1	79.46
RT2P30	Sphingomonas carotinifaciens DSM 27347 GCA 009789535.1	79.45
RT2P30	Parasphingomonas aliaeris DH-S5 GCA 016743815.1	79.44
RT2P30	Sphingomonas beigongshangi REN5 GCA 016820445.1	79.43
RT2P30	Alteristakelama pituitosa NBRC 102491 GCA 001598435.1	79.43
RT2P30	**LB3N6**	79.42
RT2P30	Sphingomonas elodea ATCC 31461 GCA 000226955.2	79.42

RT2P30	Parasphingomonas aracearum WZY 27 GCA 003345355.1	79.41
RT2P30	Sphingomonas parapaucimobilis NBRC 15100 GCA 000787715.1	79.41
RT2P30	Sphingomonas citri RRHST34 GCA 019429485.1	79.40
RT2P30	Alteristakelama koreensis JSS26 GCA 002797435.1	79.40
RT2P30	Sphingomonas yabuuchiae DSM 14562 GCA 014199595.1	79.39
RT2P30	Sphingomonas donggukensis RMG20 GCA 023674425.1	79.39
RT2P30	Sphingomonas phyllosphaerae FA2 GCA 000427645.1	79.38
RT2P30	Sphingomonas faeni MA-olki GCA 003053745.1	79.38
RT2P30	Sphingomonas albertensis DOAB 1063 GCA 014358075.1	79.37
RT2P30	**PB2P12**	79.36
RT2P30	Sphingomonas pseudosanguinis DSM 19512 GCA 014196255.1	79.36
RT2P30	Sphingomonas paucimobilis NCTC11030 GCA 900457515.1	79.33
RT2P30	**LB2R24**	79.30
RT2P30	**PB1R3**	79.30
RT2P30	Sphingomonas rubra CGMCC 1.9113 GCA 900115745.1	79.29
RT2P30	Sphingomonas yunnanensis YIM 3 GCA 019898765.1	79.29
RT2P30	Sphingomonas hominis HHU CXW GCA 013328205.1	79.23
RT2P30	Sphingomonas citricola RHCKR47 GCA 019429535.1	79.22
RT2P30	Alteristakelama suaedae XS-10 GCA 007833215.1	79.22
RT2P30	Alteristakelama soli NBRC 100801 GCA 001591025.1	79.22
RT2P30	Novistakelama desiccabilis DSM 16792 GCA 014196135.1	79.21
RT2P30	Sphingomonas ginsenosidimutans KACC 14949 GCA 002374835.1	79.20
RT2P30	Alteristakelama canadensis FWC47 GCA 026013525.1	79.18
RT2P30	Sphingomonas jeddahensis G39 GCA 001981525.1	79.18
RT2P30	**ZB1N12**	79.17
RT2P30	Sphingomonas palmae JS21-1 GCA 900109565.1	79.11
RT2P30	Parastakelama spermidinifaciens 9NM-10 GCA 002351485.1	79.11
RT2P30	Sphingomonas sanguinis NBRC 13937 GCA 001591005.1	79.06
RT2P30	Sphingomonas corticis 36D10-4-7 GCA 012035195.1	78.96
RT2P30	Sphingomonas zeae DSM 100049 GCA 014197135.1	78.95
RT2P30	Sphingomonas dokdonensis DSM 21029 GCA 002197685.1	78.94
RT2P30	Parastakelama yantingensis DSM 27244 GCA 014199325.1	78.91
RT2P30	Alterisphingomonas radiodurans S9-5 GCA 020866845.1	78.91
RT2P30	Sphingomonas changnyeongensis C33 GCA 009913435.1	78.90
RT2P30	Yabuuchia cavernae K2R01-6 GCA 003590775.1	78.81
RT2P30	Sphingomonas horti MAH-20 GCA 009753715.1	78.81
RT2P30	Sphingomonas olei NM83 B4-11 GCA 004801655.1	78.79
RT2P30	Alteristakelama xinjiangensis DSM 26736 GCA 014199255.1	78.78
RT2P30	Sphingomonas naphthae KACC 18716 GCA 028607085.1	78.75
RT2P30	Solisphingomonas oligoaromativorans DSM 102246 GCA 011762195.1	78.74
RT2P30	Alteristakelama turrisvirgatae MCT13 GCA 001721295.1	78.73
RT2P30	Sphingomonas nostoxanthinifaciens AK-PDB1-5 GCA 019930585.1	78.68
RT2P30	Novistakelama panni DSM 15761 GCA 022664435.1	78.65
RT2P30	Parayabuuchia flavalba ZLT-5 GCA 004796535.1	78.65

RT2P30	Solisphingomonas quercus XMGL2 GCA 018863195.1	78.62
RT2P30	Alteriyabuuchia sanxanigenens NX02 GCA 000512205.2	78.62
RT2P30	Novistakelama hankookensis KCTC 22579 GCA 022664465.1	78.61
RT2P30	Sphingomonas mucosissima DSM 17494 GCA 002197665.1	78.61
RT2P30	Sphingomonas gellani S6-262 GCA 900110035.1	78.59
RT2P30	Yabuuchia colocasiae JCM 31229 GCA 019880585.1	78.58
RT2P30	Sphingomonas lenta 1PNM-20 GCA 002288825.1	78.58
RT2P30	Solisphingomonas chungangi MAH-6 GCA 009763135.1	78.55
RT2P30	Pseudostakelama guangdongensis CGMCC 1.12672 GCA 900199185.1	78.52
RT2P30	Parayabuuchia changbaiensis NBRC 104936 GCA 000974765.1	78.49
RT2P30	Edaphosphingomonas haloaromaticamans P3 GCA 001853345.1	78.48
RT2P30	**LT1P40**	78.47
RT2P30	Neorhizorhabdus oleivorans FW-11 GCA 003050615.1	78.46
RT2P30	Edaphosphingomonas fennica K101 GCA 003034225.1	78.43
RT2P30	Rhizorhabdus crocodyli CCP-7 GCA 004005865.1	78.39
RT2P30	Neorhizorhabdus vulcanisoli CECT 8804 GCA 011761305.1	78.38
RT2P30	Edaphosphingomonas laterariae LNB2 GCA 900188165.1	78.36
RT2P30	Rhizorhabdus montanisoli ZX GCA 008274695.1	78.32
RT2P30	Neorhizorhabdus crusticola MIMD3 GCA 003391115.1	78.29
RT2P30	Pseudosphingomonas astaxanthinifaciens DSM 22298 GCA 000711715.1	78.29
RT2P30	Sphingomonas parva 17J27-24 GCA 004564275.1	78.25
RT2P30	Pararhizorhabdus jatrophae S5-249 GCA 900113315.1	78.23
RT2P30	Sphingomonas ursincola KR-99 GCA 013607875.1	78.23
RT2P30	Flavisphingomonas formosensis CC-Nfb-2 GCA 009755815.1	78.21
RT2P30	Pseudosphingomonas mesophila SYSUP0001 GCA 003499275.1	78.19
RT2P30	Pseudosphingomonas rhizophila KACC 19189 GCA 014396585.1	78.18
RT2P30	Pseudosphingomonas kaistensis DSM 16846 GCA 011927725.1	78.15
RT2P30	Sphingomonas glaciei S8-45 GCA 023380025.1	78.14
RT2P30	Alterirhizorhabdus solaris R4DWN GCA 007785815.1	78.14
RT2P30	Pseudosphingomonas jaspsi DSM 18422 GCA 000585415.1	78.13
RT2P30	Solisphingomonas morindae NBD5 GCA 023822065.1	78.12
RT2P30	Pararhizorhabdus prati CGMCC 1.15645 GCA 014643515.1	78.12
RT2P30	Alterirhizorhabdus profundi LMO-1 GCA 009739515.1	78.10
RT2P30	Sphingomonas aerophila DSM 100044 GCA 014199305.1	78.06
RT2P30	Parayabuuchia jejuensis DSM 27651 GCA 011927695.1	78.02
RT2P30	Pseudosphingomonas ginsengisoli KCTC 12630 GCA 003332855.1	77.99
RT2P30	Pararhizorhabdus montana W16RD GCA 001956315.1	77.91
RT2P30	Sphingomonas bisphenolicum AO1 GCA 024349785.1	77.85
RT2P30	Sphingomonas alba SE158 GCA 023516555.1	77.85
RT2P30	Sphingomonas brevis RB56-2 GCA 023516505.1	77.84
RT2P30	Pseudosphingomonas lutea KCTC 23642 GCA 014396785.1	77.83
RT2P30	Sphingomonas caseinilyticus NSE70-1 GCA 023516455.1	77.83
RT2P30	Sphingomonas cremea G124 GCA 021502585.1	77.82
RT2P30	Pseudosphingomonas ginkgonis HMF7854 GCA 003970925.1	77.80

RT2P30	Pseudosphingomonas piscis HDW15B GCA 011300455.1	77.79
RT2P30	Alterisphingopyxis lacunae CSW-10 GCA 012979535.1	77.77
RT2P30	Pseudosphingomonas sabuli sand1-3 GCA 014352855.1	77.75
RT2P30	Sphingomonas sediminicola KACC 15039 GCA 014489515.1	77.71
RT2P30	Allosphingosinicella deserti GL-C-18 GCA 003012735.1	77.69
RT2P30	Pseudosphingomonas arenae SYSU D00720 GCA 016924655.1	77.64
RT2P30	Sphingomonas hankyongi SE220 GCA 023516575.1	77.57
RT2P30	Pseudosphingomonas segetis YJ09 GCA 009720245.1	77.55
RT2P30	Pseudosphingomonas sinipercae HDW15C GCA 011302055.1	77.54
RT2P30	Sphingomonas telluris SM33 GCA 022568775.1	77.48
RT2P30	Sphingomonas xanthus AE3 GCA 007998985.1	77.45
RT2P30	Sphingomonas anseongensis RG327 GCA 023516495.1	77.45
RT2P30	Pseudosphingomonas edaphi DAC4 GCA 003583725.1	77.43
ZT3P38	Sphingomonas oligophenolica CGMCC 1.10181 GCA 039615115.1	82.80
ZT3P38	Parasphingomonas alpina DSM 22537 GCA 014490665.1	81.07
ZT3P38	Parasphingomonas hylomeconis CCTCC AB 2013304 GCA 025370105.1	81.04
ZT3P38	**RT2P30**	81.01
ZT3P38	**RB3P16**	80.85
ZT3P38	Parasphingomonas glacialis CGMCC 1.8957 GCA 014653575.1	80.82
ZT3P38	**GB1N7**	80.59
ZT3P38	Parasphingomonas echinoides ATCC 14820 GCA 000241465.1	80.44
ZT3P38	**PB4P5**	80.40
ZT3P38	Parasphingomonas qilianensis CGMCC 1.15349 GCA 039614825.1	80.38
ZT3P38	Parasphingomonas populi 3 月 7 日 GCA 004208535.1	80.32
ZT3P38	Parasphingomonas panacis DCY99 GCA 001717955.1	80.31
ZT3P38	**PB2P19**	80.00
ZT3P38	Sphingomonas ginsenosidivorax KHI67 GCA 007995065.1	79.99
ZT3P38	Alteristakelama naasensis DSM 100060 GCA 011762145.1	79.91
ZT3P38	Parasphingomonas aliaeris DH-S5 GCA 016743815.1	79.90
ZT3P38	Sphingomonas taxi ATCC 55669 GCA 000764535.1	79.90
ZT3P38	Alterisphingomonas mali NBRC 15500 GCA 001598415.1	79.82
ZT3P38	Parasphingomonas psychrolutea CGMCC 1.10106 GCA 014636175.1	79.82
ZT3P38	Sphingomonas aerolata NW12 GCA 003046295.1	79.78
ZT3P38	Alterisphingomonas pruni NBRC 15498 GCA 001598455.1	79.77
ZT3P38	Alterisphingomonas asaccharolytica NBRC 15499 GCA 001598355.1	79.72
ZT3P38	Sphingomonas caeni LB-2 GCA 026013415.1	79.70
ZT3P38	Sphingomonas tagetis MG17 GCA 024211275.1	79.70
ZT3P38	Sphingomonas liriopis RP10 GCA 024211255.1	79.70
ZT3P38	Alteristakelama kyeonggiensis DSM 101806 GCA 014196745.1	79.65
ZT3P38	Sphingomonas lycopersici MMSM20 GCA 026130605.1	79.64
ZT3P38	Alteristakelama hengshuiensis WHSC-8 GCA 000935025.1	79.63
ZT3P38	Alteristakelama gei ZFGT-11 GCA 004792685.1	79.62
ZT3P38	Alteristakelama pokkalii L3B27 GCA 003096275.1	79.62
ZT3P38	Sphingomonas aurantiaca MA101b GCA 003050705.1	79.61

ZT3P38	Alteristakelama pituitosa NBRC 102491 GCA 001598435.1	79.58
ZT3P38	Humisphingomonas gilva ZDH117 GCA 003515075.1	79.58
ZT3P38	Pseudostakelama cannabina DM2-R-LB4 GCA 021391395.1	79.54
ZT3P38	Alterisphingomonas panacisoli HKS19 GCA 007859635.1	79.53
ZT3P38	Parasphingomonas aracearum WZY 27 GCA 003345355.1	79.52
ZT3P38	Alteristakelama leidyi DSM 4733 GCA 011761945.1	79.52
ZT3P38	Alteristakelama trueperi DSM 7225 GCA 011927635.1	79.51
ZT3P38	Sphingomonas elodea ATCC 31461 GCA 000226955.2	79.48
ZT3P38	Sphingomonas albertensis DOAB 1063 GCA 014358075.1	79.47
ZT3P38	**LB3N6**	79.46
ZT3P38	**PB2P12**	79.44
ZT3P38	Sphingomonas insulae KCTC 12872 GCA 010450875.1	79.40
ZT3P38	Alteristakelama azotifigens NBRC 15497 GCA 002091475.1	79.35
ZT3P38	Sphingomonas abaci DSM 15867 GCA 014199625.1	79.34
ZT3P38	Alteristakelama koreensis JSS26 GCA 002797435.1	79.34
ZT3P38	Sphingomonas metalli CGMCC 1.15330 GCA 014641735.1	79.33
ZT3P38	Alteristakelama psychrotolerans Cra20 GCA 002796605.1	79.33
ZT3P38	**ZB1N12**	79.32
ZT3P38	Sphingomonas endophytica DSM 101535 GCA 014199415.1	79.31
ZT3P38	Parastakelama japonica DSM 22753 GCA 011762085.1	79.30
ZT3P38	Sphingomonas beigongshangi REN5 GCA 016820445.1	79.30
ZT3P38	Sphingomonas melonis DAPP-PG 224 GCA 000379045.1	79.29
ZT3P38	Sphingomonas jinjuensis YC6723 GCA 014197105.1	79.29
ZT3P38	Sphingomonas qomolangmaensis S5-59 GCA 024496245.1	79.29
ZT3P38	Sphingomonas carotinifaciens DSM 27347 GCA 009789535.1	79.29
ZT3P38	Sphingomonas aquatilis DSM 15581 GCA 014196115.1	79.27
ZT3P38	Sphingomonas donggukensis RMG20 GCA 023674425.1	79.25
ZT3P38	Sphingomonas rubra CGMCC 1.9113 GCA 900115745.1	79.25
ZT3P38	Alteristakelama canadensis FWC47 GCA 026013525.1	79.22
ZT3P38	**LB2R24**	79.22
ZT3P38	Alteristakelama soli NBRC 100801 GCA 001591025.1	79.22
ZT3P38	Sphingomonas yabuuchiae DSM 14562 GCA 014199595.1	79.19
ZT3P38	Sphingomonas yunnanensis YIM 3 GCA 019898765.1	79.18
ZT3P38	Sphingomonas parapaucimobilis NBRC 15100 GCA 000787715.1	79.17
ZT3P38	Parastakelama baiyangensis L-1-4 w-11 GCA 005144715.1	79.17
ZT3P38	Sphingomonas faeni MA-olki GCA 003053745.1	79.16
ZT3P38	Sphingomonas folli RHCKR7 GCA 019429525.1	79.16
ZT3P38	Sphingomonas adhaesiva DSM 7418 GCA 002374855.1	79.15
ZT3P38	Novistakelama desiccabilis DSM 16792 GCA 014196135.1	79.11
ZT3P38	Sphingomonas sanguinis NBRC 13937 GCA 001591005.1	79.10
ZT3P38	Sphingomonas jeddahensis G39 GCA 001981525.1	79.08
ZT3P38	**PB1R3**	79.08
ZT3P38	Sphingomonas pseudosanguinis DSM 19512 GCA 014196255.1	79.07
ZT3P38	Sphingomonas ginsenosidimutans KACC 14949 GCA 002374835.1	79.05

ZT3P38	Sphingomonas citri RRHST34 GCA 019429485.1	79.04
ZT3P38	Sphingomonas paucimobilis NCTC11030 GCA 900457515.1	79.03
ZT3P38	Alterisphingomonas radiodurans S9-5 GCA 020866845.1	79.02
ZT3P38	Alteristakelama suaedae XS-10 GCA 007833215.1	78.99
ZT3P38	Novistakelama panni DSM 15761 GCA 022664435.1	78.97
ZT3P38	Sphingomonas olei NM83 B4-11 GCA 004801655.1	78.96
ZT3P38	Sphingomonas dokdonensis DSM 21029 GCA 002197685.1	78.93
ZT3P38	Sphingomonas citricola RHCKR47 GCA 019429535.1	78.92
ZT3P38	Sphingomonas zeae DSM 100049 GCA 014197135.1	78.89
ZT3P38	Sphingomonas mucosissima DSM 17494 GCA 002197665.1	78.88
ZT3P38	Parastakelama spermidinifaciens 9NM-10 GCA 002351485.1	78.86
ZT3P38	Sphingomonas corticis 36D10-4-7 GCA 012035195.1	78.82
ZT3P38	Sphingomonas palmae JS21-1 GCA 900109565.1	78.82
ZT3P38	Alteriyabuuchia sanxanigenens NX02 GCA 000512205.2	78.81
ZT3P38	Alteristakelama xinjiangensis DSM 26736 GCA 014199255.1	78.81
ZT3P38	Sphingomonas phyllosphaerae FA2 GCA 000427645.1	78.79
ZT3P38	Parastakelama yantingensis DSM 27244 GCA 014199325.1	78.78
ZT3P38	Yabuuchia cavernae K2R01-6 GCA 003590775.1	78.76
ZT3P38	Sphingomonas changnyeongensis C33 GCA 009913435.1	78.75
ZT3P38	Alteristakelama turrisvirgatae MCT13 GCA 001721295.1	78.75
ZT3P38	Sphingomonas hominis HHU CXW GCA 013328205.1	78.73
ZT3P38	Novistakelama hankookensis KCTC 22579 GCA 022664465.1	78.73
ZT3P38	Sphingomonas horti MAH-20 GCA 009753715.1	78.73
ZT3P38	Yabuuchia colocasiae JCM 31229 GCA 019880585.1	78.72
ZT3P38	Neorhizorhabdus oleivorans FW-11 GCA 003050615.1	78.71
ZT3P38	Solisphingomonas chungangi MAH-6 GCA 009763135.1	78.70
ZT3P38	Parayabuuchia changbaiensis NBRC 104936 GCA 000974765.1	78.66
ZT3P38	Solisphingomonas oligoaromativorans DSM 102246 GCA 011762195.1	78.66
ZT3P38	Sphingomonas naphthae KACC 18716 GCA 028607085.1	78.65
ZT3P38	Solisphingomonas quercus XMGL2 GCA 018863195.1	78.65
ZT3P38	**LT1P40**	78.63
ZT3P38	Sphingomonas nostoxanthinifaciens AK-PDB1-5 GCA 019930585.1	78.60
ZT3P38	Parayabuuchia flavalba ZLT-5 GCA 004796535.1	78.57
ZT3P38	Edaphosphingomonas fennica K101 GCA 003034225.1	78.51
ZT3P38	Alterirhizorhabdus solaris R4DWN GCA 007785815.1	78.50
ZT3P38	Pararhizorhabdus jatrophae S5-249 GCA 900113315.1	78.49
ZT3P38	Edaphosphingomonas haloaromaticamans P3 GCA 001853345.1	78.48
ZT3P38	Edaphosphingomonas laterariae LNB2 GCA 900188165.1	78.47
ZT3P38	Rhizorhabdus crocodyli CCP-7 GCA 004005865.1	78.46
ZT3P38	Sphingomonas lenta 1PNM-20 GCA 002288825.1	78.44
ZT3P38	Alterirhizorhabdus profundi LMO-1 GCA 009739515.1	78.40
ZT3P38	Sphingomonas ursincola KR-99 GCA 013607875.1	78.37
ZT3P38	Sphingomonas gellani S6-262 GCA 900110035.1	78.36
ZT3P38	Flavisphingomonas formosensis CC-Nfb-2 GCA 009755815.1	78.32

ZT3P38	Sphingomonas parva 17J27-24 GCA 004564275.1	78.29
ZT3P38	Rhizorhabdus montanisoli ZX GCA 008274695.1	78.26
ZT3P38	Pseudostakelama guangdongensis CGMCC 1.12672 GCA 900199185.1	78.26
ZT3P38	Neorhizorhabdus vulcanisoli CECT 8804 GCA 011761305.1	78.25
ZT3P38	Sphingomonas aerophila DSM 100044 GCA 014199305.1	78.25
ZT3P38	Pararhizorhabdus montana W16RD GCA 001956315.1	78.18
ZT3P38	Pseudosphingomonas rhizophila KACC 19189 GCA 014396585.1	78.15
ZT3P38	Solisphingomonas morindae NBD5 GCA 023822065.1	78.13
ZT3P38	Neorhizorhabdus crusticola MIMD3 GCA 003391115.1	78.13
ZT3P38	Parayabuuchia jejuensis DSM 27651 GCA 011927695.1	78.11
ZT3P38	Pseudosphingomonas arenae SYSU D00720 GCA 016924655.1	78.05
ZT3P38	Pseudosphingomonas ginsengisoli KCTC 12630 GCA 003332855.1	78.04
ZT3P38	Pseudosphingomonas jaspsi DSM 18422 GCA 000585415.1	77.97
ZT3P38	Allosphingosinicella deserti GL-C-18 GCA 003012735.1	77.97
ZT3P38	Pararhizorhabdus prati CGMCC 1.15645 GCA 014643515.1	77.96
ZT3P38	Pseudosphingomonas astaxanthinifaciens DSM 22298 GCA 000711715.1	77.90
ZT3P38	Sphingomonas glaciei S8-45 GCA 023380025.1	77.90
ZT3P38	Pseudosphingomonas kaistensis DSM 16846 GCA 011927725.1	77.87
ZT3P38	Sphingomonas brevis RB56-2 GCA 023516505.1	77.82
ZT3P38	Sphingomonas cremea G124 GCA 021502585.1	77.81
ZT3P38	Sphingomonas bisphenolicum AO1 GCA 024349785.1	77.80
ZT3P38	Pseudosphingomonas sinipercae HDW15C GCA 011302055.1	77.78
ZT3P38	Sphingomonas alba SE158 GCA 023516555.1	77.77
ZT3P38	Sphingomonas caseinilyticus NSE70-1 GCA 023516455.1	77.76
ZT3P38	Pseudosphingomonas lutea KCTC 23642 GCA 014396785.1	77.76
ZT3P38	Pseudosphingomonas piscis HDW15B GCA 011300455.1	77.74
ZT3P38	Pseudosphingomonas segetis YJ09 GCA 009720245.1	77.72
ZT3P38	Sphingomonas anseongensis RG327 GCA 023516495.1	77.67
ZT3P38	Sphingomonas hankyongi SE220 GCA 023516575.1	77.62
ZT3P38	Pseudosphingomonas mesophila SYSUP0001 GCA 003499275.1	77.61
ZT3P38	Pseudosphingomonas sabuli sand1-3 GCA 014352855.1	77.58
ZT3P38	Pseudosphingomonas ginkgonis HMF7854 GCA 003970925.1	77.54
ZT3P38	Pseudosphingomonas daechungensis KACC 18115 GCA 014489495.1	77.49
ZT3P38	Sphingomonas telluris SM33 GCA 022568775.1	77.40
ZT3P38	Sphingomonas xanthus AE3 GCA 007998985.1	77.33
ZT3P38	Pseudosphingomonas edaphi DAC4 GCA 003583725.1	77.28
ZB1N12	Sphingomonas albertensis DOAB 1063 GCA 014358075.1	89.74
ZB1N12	Sphingomonas faeni MA-olki GCA 003053745.1	89.42
ZB1N12	**LB3N6**	89.17
ZB1N12	**LB2R24**	88.40
ZB1N12	**PB2P12**	87.65
ZB1N12	Sphingomonas aurantiaca MA101b GCA 003050705.1	87.31
ZB1N12	Sphingomonas aerolata NW12 GCA 003046295.1	84.39
ZB1N12	Sphingomonas ginsenosidivorax KHI67 GCA 007995065.1	83.51

ZB1N12	**PB2P19**	83.16
ZB1N12	Sphingomonas liriopis RP10 GCA 024211255.1	80.92
ZB1N12	Sphingomonas taxi ATCC 55669 GCA 000764535.1	80.86
ZB1N12	Parasphingomonas aliaeris DH-S5 GCA 016743815.1	80.32
ZB1N12	Sphingomonas insulae KCTC 12872 GCA 010450875.1	80.30
ZB1N12	Sphingomonas rubra CGMCC 1.9113 GCA 900115745.1	80.21
ZB1N12	Parasphingomonas glacialis CGMCC 1.8957 GCA 014653575.1	80.20
ZB1N12	Sphingomonas melonis DAPP-PG 224 GCA 000379045.1	80.18
ZB1N12	**RB3P16**	80.13
ZB1N12	Sphingomonas aquatilis DSM 15581 GCA 014196115.1	79.97
ZB1N12	Parasphingomonas hylomeconis CCTCC AB 2013304 GCA 025370105.1	79.96
ZB1N12	Sphingomonas jinjuensis YC6723 GCA 014197105.1	79.95
ZB1N12	Sphingomonas carotinifaciens DSM 27347 GCA 009789535.1	79.82
ZB1N12	Sphingomonas endophytica DSM 101535 GCA 014199415.1	79.77
ZB1N12	**GB1N7**	79.74
ZB1N12	Sphingomonas metalli CGMCC 1.15330 GCA 014641735.1	79.63
ZB1N12	Sphingomonas donggukensis RMG20 GCA 023674425.1	79.62
ZB1N12	Sphingomonas beigongshangi REN5 GCA 016820445.1	79.62
ZB1N12	Sphingomonas pseudosanguinis DSM 19512 GCA 014196255.1	79.57
ZB1N12	Parasphingomonas populi 3 月 7 日 GCA 004208535.1	79.56
ZB1N12	Sphingomonas adhaesiva DSM 7418 GCA 002374855.1	79.51
ZB1N12	Sphingomonas abaci DSM 15867 GCA 014199625.1	79.48
ZB1N12	Sphingomonas citri RRHST34 GCA 019429485.1	79.46
ZB1N12	**RT2P30**	79.45
ZB1N12	Sphingomonas folli RHCKR7 GCA 019429525.1	79.45
ZB1N12	Sphingomonas phyllosphaerae FA2 GCA 000427645.1	79.44
ZB1N12	Sphingomonas palmae JS21-1 GCA 900109565.1	79.43
ZB1N12	Sphingomonas citricola RHCKR47 GCA 019429535.1	79.41
ZB1N12	Sphingomonas parapaucimobilis NBRC 15100 GCA 000787715.1	79.41
ZB1N12	Parasphingomonas panacis DCY99 GCA 001717955.1	79.41
ZB1N12	Sphingomonas hominis HHU CXW GCA 013328205.1	79.40
ZB1N12	**ZT3P38**	79.39
ZB1N12	Sphingomonas yabuuchiae DSM 14562 GCA 014199595.1	79.39
ZB1N12	**PB1R3**	79.35
ZB1N12	Sphingomonas sanguinis NBRC 13937 GCA 001591005.1	79.34
ZB1N12	Sphingomonas paucimobilis NCTC11030 GCA 900457515.1	79.34
ZB1N12	**PB4P5**	79.32
ZB1N12	Parasphingomonas qilianensis CGMCC 1.15349 GCA 039614825.1	79.27
ZB1N12	Sphingomonas ginsenosidimutans KACC 14949 GCA 002374835.1	79.26
ZB1N12	Alteristakelama naasensis DSM 100060 GCA 011762145.1	79.25
ZB1N12	Sphingomonas yunnanensis YIM 3 GCA 019898765.1	79.25
ZB1N12	Sphingomonas qomolangmaensis S5-59 GCA 024496245.1	79.25
ZB1N12	Pseudostakelama cannabina DM2-R-LB4 GCA 021391395.1	79.25
ZB1N12	Alterisphingomonas panacisoli HKS19 GCA 007859635.1	79.22

ZB1N12	Parasphingomonas aracearum WZY 27 GCA 003345355.1	79.21
ZB1N12	Sphingomonas lycopersici MMSM20 GCA 026130605.1	79.21
ZB1N12	Alterisphingomonas radiodurans S9-5 GCA 020866845.1	79.20
ZB1N12	Sphingomonas zeae DSM 100049 GCA 014197135.1	79.13
ZB1N12	Alteristakelama pituitosa NBRC 102491 GCA 001598435.1	79.11
ZB1N12	Parasphingomonas echinoides ATCC 14820 GCA 000241465.1	79.10
ZB1N12	Alteristakelama kyeonggiensis DSM 101806 GCA 014196745.1	79.08
ZB1N12	Sphingomonas jeddahensis G39 GCA 001981525.1	79.05
ZB1N12	Alterirhizorhabdus solaris R4DWN GCA 007785815.1	79.05
ZB1N12	Sphingomonas oligophenolica CGMCC 1.10181 GCA 039615115.1	79.04
ZB1N12	Alteristakelama azotifigens NBRC 15497 GCA 002091475.1	79.03
ZB1N12	Alteristakelama hengshuiensis WHSC-8 GCA 000935025.1	79.02
ZB1N12	Parasphingomonas alpina DSM 22537 GCA 014490665.1	79.02
ZB1N12	Alteristakelama trueperi DSM 7225 GCA 011927635.1	79.01
ZB1N12	Humisphingomonas gilva ZDH117 GCA 003515075.1	78.99
ZB1N12	Novistakelama desiccabilis DSM 16792 GCA 014196135.1	78.99
ZB1N12	Sphingomonas elodea ATCC 31461 GCA 000226955.2	78.98
ZB1N12	Alteristakelama gei ZFGT-11 GCA 004792685.1	78.95
ZB1N12	Sphingomonas corticis 36D10-4-7 GCA 012035195.1	78.94
ZB1N12	Parastakelama yantingensis DSM 27244 GCA 014199325.1	78.92
ZB1N12	Sphingomonas olei NM83 B4-11 GCA 004801655.1	78.92
ZB1N12	Novistakelama panni DSM 15761 GCA 022664435.1	78.90
ZB1N12	Sphingomonas tagetis MG17 GCA 024211275.1	78.88
ZB1N12	Alteristakelama pokkalii L3B27 GCA 003096275.1	78.88
ZB1N12	Parasphingomonas psychrolutea CGMCC 1.10106 GCA 014636175.1	78.88
ZB1N12	Alteristakelama koreensis JSS26 GCA 002797435.1	78.87
ZB1N12	Sphingomonas gellani S6-262 GCA 900110035.1	78.87
ZB1N12	Novistakelama hankookensis KCTC 22579 GCA 022664465.1	78.86
ZB1N12	Sphingomonas dokdonensis DSM 21029 GCA 002197685.1	78.86
ZB1N12	Alterisphingomonas mali NBRC 15500 GCA 001598415.1	78.86
ZB1N12	Alteristakelama leidyi DSM 4733 GCA 011761945.1	78.80
ZB1N12	Parastakelama japonica DSM 22753 GCA 011762085.1	78.76
ZB1N12	Alterisphingomonas asaccharolytica NBRC 15499 GCA 001598355.1	78.75
ZB1N12	Alteristakelama psychrotolerans Cra20 GCA 002796605.1	78.74
ZB1N12	Alterisphingomonas pruni NBRC 15498 GCA 001598455.1	78.73
ZB1N12	Sphingomonas mucosissima DSM 17494 GCA 002197665.1	78.68
ZB1N12	Parastakelama spermidinifaciens 9NM-10 GCA 002351485.1	78.63
ZB1N12	Alteristakelama turrisvirgatae MCT13 GCA 001721295.1	78.62
ZB1N12	Pararhizorhabdus prati CGMCC 1.15645 GCA 014643515.1	78.59
ZB1N12	Alteristakelama soli NBRC 100801 GCA 001591025.1	78.56
ZB1N12	Parastakelama baiyangensis L-1-4 w-11 GCA 005144715.1	78.55
ZB1N12	Sphingomonas lenta 1PNM-20 GCA 002288825.1	78.55
ZB1N12	Alteristakelama suaedae XS-10 GCA 007833215.1	78.54
ZB1N12	Sphingomonas caeni LB-2 GCA 026013415.1	78.53

ZB1N12	Pararhizorhabdus montana W16RD GCA 001956315.1	78.47
ZB1N12	Sphingomonas nostoxanthinifaciens AK-PDB1-5 GCA 019930585.1	78.46
ZB1N12	Rhizorhabdus crocodyli CCP-7 GCA 004005865.1	78.46
ZB1N12	Alteriyabuuchia sanxanigenens NX02 GCA 000512205.2	78.45
ZB1N12	Alteristakelama xinjiangensis DSM 26736 GCA 014199255.1	78.41
ZB1N12	Sphingomonas naphthae KACC 18716 GCA 028607085.1	78.41
ZB1N12	Rhizorhabdus montanisoli ZX GCA 008274695.1	78.37
ZB1N12	Parayabuuchia changbaiensis NBRC 104936 GCA 000974765.1	78.34
ZB1N12	Sphingomonas horti MAH-20 GCA 009753715.1	78.34
ZB1N12	Pseudostakelama guangdongensis CGMCC 1.12672 GCA 900199185.1	78.31
ZB1N12	Sphingomonas aerophila DSM 100044 GCA 014199305.1	78.29
ZB1N12	Solisphingomonas oligoaromativorans DSM 102246 GCA 011762195.1	78.26
ZB1N12	Solisphingomonas chungangi MAH-6 GCA 009763135.1	78.23
ZB1N12	Pararhizorhabdus jatrophae S5-249 GCA 900113315.1	78.23
ZB1N12	Alteristakelama canadensis FWC47 GCA 026013525.1	78.22
ZB1N12	Yabuuchia cavernae K2R01-6 GCA 003590775.1	78.21
ZB1N12	**LT1P40**	78.17
ZB1N12	Parayabuuchia jejuensis DSM 27651 GCA 011927695.1	78.09
ZB1N12	Neorhizorhabdus crusticola MIMD3 GCA 003391115.1	78.07
ZB1N12	Sphingomonas parva 17J27-24 GCA 004564275.1	78.06
ZB1N12	Parayabuuchia flavalba ZLT-5 GCA 004796535.1	78.04
ZB1N12	Yabuuchia colocasiae JCM 31229 GCA 019880585.1	78.01
ZB1N12	Sphingomonas changnyeongensis C33 GCA 009913435.1	77.99
ZB1N12	Pseudosphingomonas astaxanthinifaciens DSM 22298 GCA 000711715.1	77.98
ZB1N12	Edaphosphingomonas fennica K101 GCA 003034225.1	77.94
ZB1N12	Alterirhizorhabdus profundi LMO-1 GCA 009739515.1	77.93
ZB1N12	Neorhizorhabdus vulcanisoli CECT 8804 GCA 011761305.1	77.90
ZB1N12	Edaphosphingomonas haloaromaticamans P3 GCA 001853345.1	77.90
ZB1N12	Edaphosphingomonas laterariae LNB2 GCA 900188165.1	77.88
ZB1N12	Pseudosphingomonas kaistensis DSM 16846 GCA 011927725.1	77.86
ZB1N12	Solisphingomonas quercus XMGL2 GCA 018863195.1	77.86
ZB1N12	Neorhizorhabdus oleivorans FW-11 GCA 003050615.1	77.86
ZB1N12	Pseudosphingomonas ginsengisoli KCTC 12630 GCA 003332855.1	77.82
ZB1N12	Pseudosphingomonas rhizophila KACC 19189 GCA 014396585.1	77.81
ZB1N12	Sphingomonas glaciei S8-45 GCA 023380025.1	77.80
ZB1N12	Flavisphingomonas formosensis CC-Nfb-2 GCA 009755815.1	77.79
ZB1N12	Sphingomonas ursincola KR-99 GCA 013607875.1	77.74
ZB1N12	Pseudosphingomonas lutea KCTC 23642 GCA 014396785.1	77.72
ZB1N12	Sphingomonas alba SE158 GCA 023516555.1	77.71
ZB1N12	Pseudosphingomonas ginkgonis HMF7854 GCA 003970925.1	77.69
ZB1N12	Sphingomonas bisphenolicum AO1 GCA 024349785.1	77.68
ZB1N12	Pseudosphingomonas jaspsi DSM 18422 GCA 000585415.1	77.66
ZB1N12	Sphingomonas brevis RB56-2 GCA 023516505.1	77.61
ZB1N12	Allosphingosinicella deserti GL-C-18 GCA 003012735.1	77.59

ZB1N12	Pseudosphingomonas sabuli sand1-3 GCA 014352855.1	77.57
ZB1N12	Solisphingomonas morindae NBD5 GCA 023822065.1	77.54
ZB1N12	Sphingomonas telluris SM33 GCA 022568775.1	77.46
ZB1N12	Pseudosphingomonas sinipercae HDW15C GCA 011302055.1	77.44
ZB1N12	Pseudosphingomonas mesophila SYSUP0001 GCA 003499275.1	77.44
ZB1N12	Pseudosphingomonas piscis HDW15B GCA 011300455.1	77.41
ZB1N12	Sphingomonas anseongensis RG327 GCA 023516495.1	77.38
ZB1N12	Pseudosphingomonas segetis YJ09 GCA 009720245.1	77.36
ZB1N12	Sphingomonas sediminicola KACC 15039 GCA 014489515.1	77.32
ZB1N12	Pseudosphingomonas arenae SYSU D00720 GCA 016924655.1	77.22
ZB1N12	Sphingomonas xanthus AE3 GCA 007998985.1	77.10
GB1N7	Parasphingomonas aliaeris DH-S5 GCA 016743815.1	83.73
GB1N7	Parasphingomonas hylomeconis CCTCC AB 2013304 GCA 025370105.1	83.30
GB1N7	**PB4P5**	82.74
GB1N7	Parasphingomonas qilianensis CGMCC 1.15349 GCA 039614825.1	82.09
GB1N7	Parasphingomonas alpina DSM 22537 GCA 014490665.1	80.94
GB1N7	**RB3P16**	80.74
GB1N7	Parasphingomonas glacialis CGMCC 1.8957 GCA 014653575.1	80.71
GB1N7	**ZT3P38**	80.65
GB1N7	Parasphingomonas echinoides ATCC 14820 GCA 000241465.1	80.48
GB1N7	Parasphingomonas panacis DCY99 GCA 001717955.1	80.43
GB1N7	Parasphingomonas populi 3 月 7 日 GCA 004208535.1	80.38
GB1N7	**RT2P30**	80.34
GB1N7	Parasphingomonas psychrolutea CGMCC 1.10106 GCA 014636175.1	80.30
GB1N7	Sphingomonas ginsenosidivorax KHI67 GCA 007995065.1	80.28
GB1N7	**PB2P19**	80.24
GB1N7	Sphingomonas liriopis RP10 GCA 024211255.1	80.07
GB1N7	Sphingomonas aurantiaca MA101b GCA 003050705.1	80.04
GB1N7	Sphingomonas taxi ATCC 55669 GCA 000764535.1	79.97
GB1N7	Sphingomonas oligophenolica CGMCC 1.10181 GCA 039615115.1	79.97
GB1N7	**LB3N6**	79.92
GB1N7	**ZB1N12**	79.85
GB1N7	Sphingomonas aerolata NW12 GCA 003046295.1	79.84
GB1N7	Sphingomonas melonis DAPP-PG 224 GCA 000379045.1	79.84
GB1N7	Sphingomonas insulae KCTC 12872 GCA 010450875.1	79.78
GB1N7	Sphingomonas aquatilis DSM 15581 GCA 014196115.1	79.75
GB1N7	Sphingomonas adhaesiva DSM 7418 GCA 002374855.1	79.72
GB1N7	**PB2P12**	79.68
GB1N7	**LB2R24**	79.66
GB1N7	Sphingomonas lycopersici MMSM20 GCA 026130605.1	79.64
GB1N7	Sphingomonas albertensis DOAB 1063 GCA 014358075.1	79.60
GB1N7	Alterisphingomonas panacisoli HKS19 GCA 007859635.1	79.59
GB1N7	Sphingomonas faeni MA-olki GCA 003053745.1	79.51
GB1N7	Parasphingomonas aracearum WZY 27 GCA 003345355.1	79.50

GB1N7	Sphingomonas jinjuensis YC6723 GCA 014197105.1	79.49
GB1N7	Sphingomonas endophytica DSM 101535 GCA 014199415.1	79.47
GB1N7	Alterisphingomonas pruni NBRC 15498 GCA 001598455.1	79.42
GB1N7	Alterisphingomonas asaccharolytica NBRC 15499 GCA 001598355.1	79.42
GB1N7	Sphingomonas qomolangmaensis S5-59 GCA 024496245.1	79.40
GB1N7	Sphingomonas donggukensis RMG20 GCA 023674425.1	79.39
GB1N7	Humisphingomonas gilva ZDH117 GCA 003515075.1	79.38
GB1N7	Sphingomonas abaci DSM 15867 GCA 014199625.1	79.37
GB1N7	Sphingomonas tagetis MG17 GCA 024211275.1	79.37
GB1N7	Alterisphingomonas mali NBRC 15500 GCA 001598415.1	79.37
GB1N7	Sphingomonas ginsenosidimutans KACC 14949 GCA 002374835.1	79.32
GB1N7	Sphingomonas rubra CGMCC 1.9113 GCA 900115745.1	79.30
GB1N7	Alteristakelama hengshuiensis WHSC-8 GCA 000935025.1	79.30
GB1N7	Parastakelama japonica DSM 22753 GCA 011762085.1	79.29
GB1N7	Sphingomonas carotinifaciens DSM 27347 GCA 009789535.1	79.27
GB1N7	Sphingomonas dokdonensis DSM 21029 GCA 002197685.1	79.27
GB1N7	Alteristakelama koreensis JSS26 GCA 002797435.1	79.25
GB1N7	Sphingomonas pseudosanguinis DSM 19512 GCA 014196255.1	79.24
GB1N7	Sphingomonas metalli CGMCC 1.15330 GCA 014641735.1	79.23
GB1N7	Alterisphingomonas radiodurans S9-5 GCA 020866845.1	79.22
GB1N7	Alteristakelama kyeonggiensis DSM 101806 GCA 014196745.1	79.21
GB1N7	Sphingomonas caeni LB-2 GCA 026013415.1	79.20
GB1N7	Alteristakelama canadensis FWC47 GCA 026013525.1	79.18
GB1N7	Sphingomonas phyllosphaerae FA2 GCA 000427645.1	79.17
GB1N7	Pseudostakelama cannabina DM2-R-LB4 GCA 021391395.1	79.17
GB1N7	Alteristakelama naasensis DSM 100060 GCA 011762145.1	79.17
GB1N7	Alteristakelama suaedae XS-10 GCA 007833215.1	79.16
GB1N7	Alteristakelama soli NBRC 100801 GCA 001591025.1	79.16
GB1N7	Sphingomonas yabuuchiae DSM 14562 GCA 014199595.1	79.13
GB1N7	Sphingomonas beigongshangi REN5 GCA 016820445.1	79.13
GB1N7	**LT1P40**	79.12
GB1N7	Sphingomonas sanguinis NBRC 13937 GCA 001591005.1	79.12
GB1N7	Sphingomonas hominis HHU CXW GCA 013328205.1	79.11
GB1N7	Alteristakelama trueperi DSM 7225 GCA 011927635.1	79.11
GB1N7	Novistakelama panni DSM 15761 GCA 022664435.1	79.10
GB1N7	Alteristakelama gei ZFGT-11 GCA 004792685.1	79.08
GB1N7	Sphingomonas paucimobilis NCTC11030 GCA 900457515.1	79.07
GB1N7	Sphingomonas parapaucimobilis NBRC 15100 GCA 000787715.1	79.05
GB1N7	Novistakelama hankookensis KCTC 22579 GCA 022664465.1	79.04
GB1N7	Alteristakelama pituitosa NBRC 102491 GCA 001598435.1	79.02
GB1N7	Alteristakelama pokkalii L3B27 GCA 003096275.1	78.99
GB1N7	Alteristakelama leidyi DSM 4733 GCA 011761945.1	78.99
GB1N7	Sphingomonas jeddahensis G39 GCA 001981525.1	78.98
GB1N7	**PB1R3**	78.94

GB1N7	Sphingomonas elodea ATCC 31461 GCA 000226955.2	78.92
GB1N7	Sphingomonas citricola RHCKR47 GCA 019429535.1	78.92
GB1N7	Sphingomonas yunnanensis YIM 3 GCA 019898765.1	78.91
GB1N7	Novistakelama desiccabilis DSM 16792 GCA 014196135.1	78.91
GB1N7	Sphingomonas citri RRHST34 GCA 019429485.1	78.86
GB1N7	Alteristakelama psychrotolerans Cra20 GCA 002796605.1	78.86
GB1N7	Parastakelama spermidinifaciens 9NM-10 GCA 002351485.1	78.85
GB1N7	Sphingomonas corticis 36D10-4-7 GCA 012035195.1	78.85
GB1N7	Parastakelama yantingensis DSM 27244 GCA 014199325.1	78.84
GB1N7	Alteristakelama azotifigens NBRC 15497 GCA 002091475.1	78.84
GB1N7	Parastakelama baiyangensis L-1-4 w-11 GCA 005144715.1	78.81
GB1N7	Sphingomonas olei NM83 B4-11 GCA 004801655.1	78.80
GB1N7	Sphingomonas folli RHCKR7 GCA 019429525.1	78.79
GB1N7	Alteristakelama turrisvirgatae MCT13 GCA 001721295.1	78.77
GB1N7	Sphingomonas nostoxanthinifaciens AK-PDB1-5 GCA 019930585.1	78.76
GB1N7	Sphingomonas zeae DSM 100049 GCA 014197135.1	78.75
GB1N7	Sphingomonas palmae JS21-1 GCA 900109565.1	78.68
GB1N7	Alteristakelama xinjiangensis DSM 26736 GCA 014199255.1	78.68
GB1N7	Sphingomonas gellani S6-262 GCA 900110035.1	78.58
GB1N7	Sphingomonas mucosissima DSM 17494 GCA 002197665.1	78.58
GB1N7	Alterirhizorhabdus solaris R4DWN GCA 007785815.1	78.58
GB1N7	Alteriyabuuchia sanxanigenens NX02 GCA 000512205.2	78.57
GB1N7	Sphingomonas naphthae KACC 18716 GCA 028607085.1	78.55
GB1N7	Alterirhizorhabdus profundi LMO-1 GCA 009739515.1	78.50
GB1N7	Yabuuchia colocasiae JCM 31229 GCA 019880585.1	78.43
GB1N7	Rhizorhabdus crocodyli CCP-7 GCA 004005865.1	78.40
GB1N7	Parayabuuchia flavalba ZLT-5 GCA 004796535.1	78.40
GB1N7	Pararhizorhabdus montana W16RD GCA 001956315.1	78.40
GB1N7	Parayabuuchia changbaiensis NBRC 104936 GCA 000974765.1	78.40
GB1N7	Solisphingomonas chungangi MAH-6 GCA 009763135.1	78.39
GB1N7	Sphingomonas changnyeongensis C33 GCA 009913435.1	78.39
GB1N7	Edaphosphingomonas haloaromaticamans P3 GCA 001853345.1	78.39
GB1N7	Pseudostakelama guangdongensis CGMCC 1.12672 GCA 900199185.1	78.37
GB1N7	Edaphosphingomonas fennica K101 GCA 003034225.1	78.37
GB1N7	Sphingomonas horti MAH-20 GCA 009753715.1	78.37
GB1N7	Solisphingomonas oligoaromativorans DSM 102246 GCA 011762195.1	78.36
GB1N7	Rhizorhabdus montanisoli ZX GCA 008274695.1	78.34
GB1N7	Edaphosphingomonas laterariae LNB2 GCA 900188165.1	78.31
GB1N7	Pararhizorhabdus prati CGMCC 1.15645 GCA 014643515.1	78.27
GB1N7	Pararhizorhabdus jatrophae S5-249 GCA 900113315.1	78.23
GB1N7	Yabuuchia cavernae K2R01-6 GCA 003590775.1	78.21
GB1N7	Neorhizorhabdus vulcanisoli CECT 8804 GCA 011761305.1	78.14
GB1N7	Neorhizorhabdus crusticola MIMD3 GCA 003391115.1	78.13
GB1N7	Solisphingomonas quercus XMGL2 GCA 018863195.1	78.11

GB1N7	Sphingomonas lenta 1PNM-20 GCA 002288825.1	78.10
GB1N7	Sphingomonas aerophila DSM 100044 GCA 014199305.1	78.03
GB1N7	Sphingomonas bisphenolicum AO1 GCA 024349785.1	77.97
GB1N7	Neorhizorhabdus oleivorans FW-11 GCA 003050615.1	77.96
GB1N7	Pseudosphingomonas jaspsi DSM 18422 GCA 000585415.1	77.89
GB1N7	Sphingomonas ursincola KR-99 GCA 013607875.1	77.89
GB1N7	Parayabuuchia jejuensis DSM 27651 GCA 011927695.1	77.88
GB1N7	Flavisphingomonas formosensis CC-Nfb-2 GCA 009755815.1	77.87
GB1N7	Sphingomonas brevis RB56-2 GCA 023516505.1	77.87
GB1N7	Sphingomonas glaciei S8-45 GCA 023380025.1	77.86
GB1N7	Pseudosphingomonas kaistensis DSM 16846 GCA 011927725.1	77.78
GB1N7	Solisphingomonas morindae NBD5 GCA 023822065.1	77.77
GB1N7	Pseudosphingomonas astaxanthinifaciens DSM 22298 GCA 000711715.1	77.74
GB1N7	Sphingomonas parva 17J27-24 GCA 004564275.1	77.71
GB1N7	Pseudosphingomonas sinipercae HDW15C GCA 011302055.1	77.70
GB1N7	Pseudosphingomonas ginsengisoli KCTC 12630 GCA 003332855.1	77.69
GB1N7	Pseudosphingomonas sabuli sand1-3 GCA 014352855.1	77.61
GB1N7	Pseudosphingomonas rhizophila KACC 19189 GCA 014396585.1	77.59
GB1N7	Pseudosphingomonas mesophila SYSUP0001 GCA 003499275.1	77.57
GB1N7	Pseudosphingomonas segetis YJ09 GCA 009720245.1	77.51
GB1N7	Pseudosphingomonas lutea KCTC 23642 GCA 014396785.1	77.42
GB1N7	Sphingomonas alba SE158 GCA 023516555.1	77.40
GB1N7	Sphingomonas xanthus AE3 GCA 007998985.1	77.40
GB1N7	Allosphingosinicella deserti GL-C-18 GCA 003012735.1	77.39
GB1N7	Sphingomonas anseongensis RG327 GCA 023516495.1	77.30
GB1N7	Pseudosphingomonas ginkgonis HMF7854 GCA 003970925.1	77.19
GB1N7	Pseudosphingomonas arenae SYSU D00720 GCA 016924655.1	77.05
PB2P12	Sphingomonas albertensis DOAB 1063 GCA 014358075.1	88.05
PB2P12	**LB2R24**	87.89
PB2P12	Sphingomonas faeni MA-olki GCA 003053745.1	87.77
PB2P12	**LB3N6**	87.66
PB2P12	**ZB1N12**	87.39
PB2P12	Sphingomonas aurantiaca MA101b GCA 003050705.1	87.10
PB2P12	Sphingomonas aerolata NW12 GCA 003046295.1	84.42
PB2P12	Sphingomonas ginsenosidivorax KHI67 GCA 007995065.1	83.30
PB2P12	**PB2P19**	82.87
PB2P12	Sphingomonas liriopis RP10 GCA 024211255.1	80.81
PB2P12	Sphingomonas taxi ATCC 55669 GCA 000764535.1	80.75
PB2P12	Sphingomonas insulae KCTC 12872 GCA 010450875.1	80.38
PB2P12	**RB3P16**	80.18
PB2P12	Sphingomonas melonis DAPP-PG 224 GCA 000379045.1	80.12
PB2P12	Parasphingomonas glacialis CGMCC 1.8957 GCA 014653575.1	80.11
PB2P12	Sphingomonas aquatilis DSM 15581 GCA 014196115.1	80.07
PB2P12	Sphingomonas jinjuensis YC6723 GCA 014197105.1	80.03

PB2P12	Parasphingomonas hylomeconis CCTCC AB 2013304 GCA 025370105.1	79.98
PB2P12	Sphingomonas rubra CGMCC 1.9113 GCA 900115745.1	79.87
PB2P12	**GB1N7**	79.71
PB2P12	Sphingomonas endophytica DSM 101535 GCA 014199415.1	79.66
PB2P12	Sphingomonas pseudosanguinis DSM 19512 GCA 014196255.1	79.62
PB2P12	Sphingomonas abaci DSM 15867 GCA 014199625.1	79.60
PB2P12	Sphingomonas adhaesiva DSM 7418 GCA 002374855.1	79.57
PB2P12	Sphingomonas parapaucimobilis NBRC 15100 GCA 000787715.1	79.56
PB2P12	Parasphingomonas populi 3 月 7 日 GCA 004208535.1	79.55
PB2P12	Sphingomonas carotinifaciens DSM 27347 GCA 009789535.1	79.54
PB2P12	Parasphingomonas panacis DCY99 GCA 001717955.1	79.54
PB2P12	Sphingomonas phyllosphaerae FA2 GCA 000427645.1	79.53
PB2P12	Sphingomonas metalli CGMCC 1.15330 GCA 014641735.1	79.51
PB2P12	Sphingomonas beigongshangi REN5 GCA 016820445.1	79.48
PB2P12	Sphingomonas citricola RHCKR47 GCA 019429535.1	79.44
PB2P12	Sphingomonas yabuuchiae DSM 14562 GCA 014199595.1	79.42
PB2P12	**PB1R3**	79.38
PB2P12	**ZT3P38**	79.36
PB2P12	Sphingomonas ginsenosidimutans KACC 14949 GCA 002374835.1	79.35
PB2P12	Sphingomonas sanguinis NBRC 13937 GCA 001591005.1	79.35
PB2P12	Sphingomonas folli RHCKR7 GCA 019429525.1	79.33
PB2P12	Sphingomonas hominis HHU CXW GCA 013328205.1	79.32
PB2P12	**RT2P30**	79.30
PB2P12	**PB4P5**	79.27
PB2P12	Parasphingomonas echinoides ATCC 14820 GCA 000241465.1	79.26
PB2P12	Sphingomonas citri RRHST34 GCA 019429485.1	79.24
PB2P12	Parasphingomonas aliaeris DH-S5 GCA 016743815.1	79.24
PB2P12	Sphingomonas donggukensis RMG20 GCA 023674425.1	79.23
PB2P12	Sphingomonas yunnanensis YIM 3 GCA 019898765.1	79.21
PB2P12	Sphingomonas zeae DSM 100049 GCA 014197135.1	79.20
PB2P12	Alterisphingomonas radiodurans S9-5 GCA 020866845.1	79.20
PB2P12	Sphingomonas paucimobilis NCTC11030 GCA 900457515.1	79.19
PB2P12	Sphingomonas lycopersici MMSM20 GCA 026130605.1	79.19
PB2P12	Parasphingomonas qilianensis CGMCC 1.15349 GCA 039614825.1	79.18
PB2P12	Sphingomonas palmae JS21-1 GCA 900109565.1	79.15
PB2P12	Pseudostakelama cannabina DM2-R-LB4 GCA 021391395.1	79.12
PB2P12	Sphingomonas qomolangmaensis S5-59 GCA 024496245.1	79.12
PB2P12	Alteristakelama naasensis DSM 100060 GCA 011762145.1	79.11
PB2P12	Alterisphingomonas panacisoli HKS19 GCA 007859635.1	79.08
PB2P12	Novistakelama desiccabilis DSM 16792 GCA 014196135.1	79.07
PB2P12	Sphingomonas corticis 36D10-4-7 GCA 012035195.1	79.06
PB2P12	Sphingomonas tagetis MG17 GCA 024211275.1	79.03
PB2P12	Sphingomonas jeddahensis G39 GCA 001981525.1	79.00
PB2P12	Parasphingomonas psychrolutea CGMCC 1.10106 GCA 014636175.1	78.99

PB2P12	Parasphingomonas aracearum WZY 27 GCA 003345355.1	78.96
PB2P12	Parasphingomonas alpina DSM 22537 GCA 014490665.1	78.96
PB2P12	Parastakelama japonica DSM 22753 GCA 011762085.1	78.94
PB2P12	Sphingomonas oligophenolica CGMCC 1.10181 GCA 039615115.1	78.93
PB2P12	Alteristakelama trueperi DSM 7225 GCA 011927635.1	78.92
PB2P12	Alterisphingomonas mali NBRC 15500 GCA 001598415.1	78.88
PB2P12	Sphingomonas elodea ATCC 31461 GCA 000226955.2	78.87
PB2P12	Parastakelama yantingensis DSM 27244 GCA 014199325.1	78.87
PB2P12	Novistakelama panni DSM 15761 GCA 022664435.1	78.86
PB2P12	Sphingomonas olei NM83 B4-11 GCA 004801655.1	78.85
PB2P12	Alteristakelama kyeonggiensis DSM 101806 GCA 014196745.1	78.83
PB2P12	Alteristakelama azotifigens NBRC 15497 GCA 002091475.1	78.82
PB2P12	Alterisphingomonas pruni NBRC 15498 GCA 001598455.1	78.82
PB2P12	Alteristakelama hengshuiensis WHSC-8 GCA 000935025.1	78.82
PB2P12	Novistakelama hankookensis KCTC 22579 GCA 022664465.1	78.80
PB2P12	Alteristakelama pokkalii L3B27 GCA 003096275.1	78.79
PB2P12	Sphingomonas dokdonensis DSM 21029 GCA 002197685.1	78.79
PB2P12	Alteristakelama pituitosa NBRC 102491 GCA 001598435.1	78.77
PB2P12	Alteristakelama koreensis JSS26 GCA 002797435.1	78.75
PB2P12	Sphingomonas gellani S6-262 GCA 900110035.1	78.73
PB2P12	Humisphingomonas gilva ZDH117 GCA 003515075.1	78.73
PB2P12	Alterisphingomonas asaccharolytica NBRC 15499 GCA 001598355.1	78.70
PB2P12	Pararhizorhabdus prati CGMCC 1.15645 GCA 014643515.1	78.67
PB2P12	Parastakelama spermidinifaciens 9NM-10 GCA 002351485.1	78.67
PB2P12	Alteristakelama leidyi DSM 4733 GCA 011761945.1	78.65
PB2P12	Sphingomonas mucosissima DSM 17494 GCA 002197665.1	78.65
PB2P12	Alteristakelama psychrotolerans Cra20 GCA 002796605.1	78.65
PB2P12	Parastakelama baiyangensis L-1-4 w-11 GCA 005144715.1	78.64
PB2P12	Alteristakelama gei ZFGT-11 GCA 004792685.1	78.62
PB2P12	Sphingomonas lenta 1PNM-20 GCA 002288825.1	78.53
PB2P12	Sphingomonas caeni LB-2 GCA 026013415.1	78.52
PB2P12	Pseudostakelama guangdongensis CGMCC 1.12672 GCA 900199185.1	78.49
PB2P12	Pararhizorhabdus montana W16RD GCA 001956315.1	78.44
PB2P12	Alteristakelama soli NBRC 100801 GCA 001591025.1	78.44
PB2P12	Alteristakelama turrisvirgatae MCT13 GCA 001721295.1	78.42
PB2P12	Alterirhizorhabdus solaris R4DWN GCA 007785815.1	78.41
PB2P12	Yabuuchia cavernae K2R01-6 GCA 003590775.1	78.40
PB2P12	Alteristakelama suaedae XS-10 GCA 007833215.1	78.39
PB2P12	Alteriyabuuchia sanxanigenens NX02 GCA 000512205.2	78.36
PB2P12	**LT1P40**	78.34
PB2P12	Alteristakelama canadensis FWC47 GCA 026013525.1	78.32
PB2P12	Sphingomonas aerophila DSM 100044 GCA 014199305.1	78.32
PB2P12	Sphingomonas naphthae KACC 18716 GCA 028607085.1	78.32
PB2P12	Sphingomonas nostoxanthinifaciens AK-PDB1-5 GCA 019930585.1	78.27

PB2P12	Rhizorhabdus crocodyli CCP-7 GCA 004005865.1	78.27
PB2P12	Alteristakelama xinjiangensis DSM 26736 GCA 014199255.1	78.23
PB2P12	Solisphingomonas oligoaromativorans DSM 102246 GCA 011762195.1	78.22
PB2P12	Rhizorhabdus montanisoli ZX GCA 008274695.1	78.21
PB2P12	Solisphingomonas quercus XMGL2 GCA 018863195.1	78.20
PB2P12	Parayabuuchia flavalba ZLT-5 GCA 004796535.1	78.19
PB2P12	Parayabuuchia jejuensis DSM 27651 GCA 011927695.1	78.10
PB2P12	Solisphingomonas chungangi MAH-6 GCA 009763135.1	78.09
PB2P12	Yabuuchia colocasiae JCM 31229 GCA 019880585.1	78.08
PB2P12	Sphingomonas horti MAH-20 GCA 009753715.1	78.05
PB2P12	Sphingomonas changnyeongensis C33 GCA 009913435.1	78.01
PB2P12	Parayabuuchia changbaiensis NBRC 104936 GCA 000974765.1	78.01
PB2P12	Alterirhizorhabdus profundi LMO-1 GCA 009739515.1	77.97
PB2P12	Pseudosphingomonas astaxanthinifaciens DSM 22298 GCA 000711715.1	77.96
PB2P12	Pararhizorhabdus jatrophae S5-249 GCA 900113315.1	77.91
PB2P12	Pseudosphingomonas lutea KCTC 23642 GCA 014396785.1	77.87
PB2P12	Neorhizorhabdus crusticola MIMD3 GCA 003391115.1	77.84
PB2P12	Pseudosphingomonas rhizophila KACC 19189 GCA 014396585.1	77.83
PB2P12	Edaphosphingomonas laterariae LNB2 GCA 900188165.1	77.81
PB2P12	Flavisphingomonas formosensis CC-Nfb-2 GCA 009755815.1	77.79
PB2P12	Sphingomonas parva 17J27-24 GCA 004564275.1	77.78
PB2P12	Pseudosphingomonas kaistensis DSM 16846 GCA 011927725.1	77.78
PB2P12	Pseudosphingomonas jaspsi DSM 18422 GCA 000585415.1	77.74
PB2P12	Edaphosphingomonas haloaromaticamans P3 GCA 001853345.1	77.72
PB2P12	Sphingomonas ursincola KR-99 GCA 013607875.1	77.71
PB2P12	Edaphosphingomonas fennica K101 GCA 003034225.1	77.69
PB2P12	Pseudosphingomonas ginsengisoli KCTC 12630 GCA 003332855.1	77.68
PB2P12	Neorhizorhabdus vulcanisoli CECT 8804 GCA 011761305.1	77.66
PB2P12	Pseudosphingomonas ginkgonis HMF7854 GCA 003970925.1	77.66
PB2P12	Sphingomonas glaciei S8-45 GCA 023380025.1	77.65
PB2P12	Sphingomonas bisphenolicum AO1 GCA 024349785.1	77.62
PB2P12	Sphingomonas alba SE158 GCA 023516555.1	77.58
PB2P12	Neorhizorhabdus oleivorans FW-11 GCA 003050615.1	77.57
PB2P12	Pseudosphingomonas sinipercae HDW15C GCA 011302055.1	77.54
PB2P12	Sphingomonas brevis RB56-2 GCA 023516505.1	77.51
PB2P12	Solisphingomonas morindae NBD5 GCA 023822065.1	77.49
PB2P12	Pseudosphingomonas mesophila SYSUP0001 GCA 003499275.1	77.43
PB2P12	Pseudosphingomonas sabuli sand1-3 GCA 014352855.1	77.42
PB2P12	Allosphingosinicella deserti GL-C-18 GCA 003012735.1	77.40
PB2P12	Pseudosphingomonas segetis YJ09 GCA 009720245.1	77.40
PB2P12	Sphingomonas anseongensis RG327 GCA 023516495.1	77.14
PB2P19	Sphingomonas ginsenosidivorax KHI67 GCA 007995065.1	87.17
PB2P19	Sphingomonas aurantiaca MA101b GCA 003050705.1	83.68
PB2P19	Sphingomonas aerolata NW12 GCA 003046295.1	83.64

PB2P19	**LB3N6**	83.42
PB2P19	Sphingomonas albertensis DOAB 1063 GCA 014358075.1	83.31
PB2P19	**ZB1N12**	83.24
PB2P19	Sphingomonas faeni MA-olki GCA 003053745.1	83.20
PB2P19	**LB2R24**	83.20
PB2P19	**PB2P12**	83.00
PB2P19	Sphingomonas liriopis RP10 GCA 024211255.1	81.73
PB2P19	Sphingomonas taxi ATCC 55669 GCA 000764535.1	81.54
PB2P19	**RB3P16**	80.82
PB2P19	Sphingomonas insulae KCTC 12872 GCA 010450875.1	80.82
PB2P19	Sphingomonas aquatilis DSM 15581 GCA 014196115.1	80.77
PB2P19	Parasphingomonas glacialis CGMCC 1.8957 GCA 014653575.1	80.73
PB2P19	Sphingomonas melonis DAPP-PG 224 GCA 000379045.1	80.69
PB2P19	Sphingomonas jinjuensis YC6723 GCA 014197105.1	80.63
PB2P19	Parasphingomonas hylomeconis CCTCC AB 2013304 GCA 025370105.1	80.62
PB2P19	Sphingomonas rubra CGMCC 1.9113 GCA 900115745.1	80.46
PB2P19	Sphingomonas abaci DSM 15867 GCA 014199625.1	80.35
PB2P19	**GB1N7**	80.30
PB2P19	**RT2P30**	80.24
PB2P19	Sphingomonas beigongshangi REN5 GCA 016820445.1	80.22
PB2P19	Sphingomonas carotinifaciens DSM 27347 GCA 009789535.1	80.21
PB2P19	Sphingomonas endophytica DSM 101535 GCA 014199415.1	80.18
PB2P19	**PB4P5**	80.18
PB2P19	Sphingomonas adhaesiva DSM 7418 GCA 002374855.1	80.16
PB2P19	Sphingomonas donggukensis RMG20 GCA 023674425.1	80.07
PB2P19	Sphingomonas metalli CGMCC 1.15330 GCA 014641735.1	80.05
PB2P19	Sphingomonas pseudosanguinis DSM 19512 GCA 014196255.1	80.02
PB2P19	**ZT3P38**	80.00
PB2P19	Parasphingomonas qilianensis CGMCC 1.15349 GCA 039614825.1	80.00
PB2P19	Sphingomonas folli RHCKR7 GCA 019429525.1	79.96
PB2P19	Parasphingomonas panacis DCY99 GCA 001717955.1	79.94
PB2P19	Sphingomonas qomolangmaensis S5-59 GCA 024496245.1	79.93
PB2P19	**PB1R3**	79.92
PB2P19	Sphingomonas phyllosphaerae FA2 GCA 000427645.1	79.92
PB2P19	Sphingomonas ginsenosidimutans KACC 14949 GCA 002374835.1	79.92
PB2P19	Sphingomonas citri RRHST34 GCA 019429485.1	79.91
PB2P19	Pseudostakelama cannabina DM2-R-LB4 GCA 021391395.1	79.89
PB2P19	Sphingomonas lycopersici MMSM20 GCA 026130605.1	79.89
PB2P19	Sphingomonas parapaucimobilis NBRC 15100 GCA 000787715.1	79.88
PB2P19	Alteristakelama naasensis DSM 100060 GCA 011762145.1	79.87
PB2P19	Sphingomonas yabuuchiae DSM 14562 GCA 014199595.1	79.81
PB2P19	Sphingomonas yunnanensis YIM 3 GCA 019898765.1	79.80
PB2P19	Parasphingomonas populi 3 月 7 日 GCA 004208535.1	79.76
PB2P19	Sphingomonas hominis HHU CXW GCA 013328205.1	79.75

PB2P19	Sphingomonas citricola RHCKR47 GCA 019429535.1	79.74
PB2P19	Sphingomonas oligophenolica CGMCC 1.10181 GCA 039615115.1	79.72
PB2P19	Parasphingomonas echinoides ATCC 14820 GCA 000241465.1	79.70
PB2P19	Sphingomonas palmae JS21-1 GCA 900109565.1	79.67
PB2P19	Sphingomonas paucimobilis NCTC11030 GCA 900457515.1	79.67
PB2P19	Sphingomonas zeae DSM 100049 GCA 014197135.1	79.67
PB2P19	Parasphingomonas aliaeris DH-S5 GCA 016743815.1	79.63
PB2P19	Sphingomonas sanguinis NBRC 13937 GCA 001591005.1	79.60
PB2P19	Alteristakelama pituitosa NBRC 102491 GCA 001598435.1	79.58
PB2P19	Parasphingomonas alpina DSM 22537 GCA 014490665.1	79.57
PB2P19	Alteristakelama trueperi DSM 7225 GCA 011927635.1	79.57
PB2P19	Alteristakelama hengshuiensis WHSC-8 GCA 000935025.1	79.56
PB2P19	Sphingomonas jeddahensis G39 GCA 001981525.1	79.56
PB2P19	Alterisphingomonas panacisoli HKS19 GCA 007859635.1	79.54
PB2P19	Novistakelama desiccabilis DSM 16792 GCA 014196135.1	79.54
PB2P19	Alteristakelama gei ZFGT-11 GCA 004792685.1	79.53
PB2P19	Parastakelama japonica DSM 22753 GCA 011762085.1	79.53
PB2P19	Alterisphingomonas radiodurans S9-5 GCA 020866845.1	79.53
PB2P19	Humisphingomonas gilva ZDH117 GCA 003515075.1	79.53
PB2P19	Sphingomonas elodea ATCC 31461 GCA 000226955.2	79.49
PB2P19	Alterisphingomonas asaccharolytica NBRC 15499 GCA 001598355.1	79.48
PB2P19	Sphingomonas tagetis MG17 GCA 024211275.1	79.48
PB2P19	Sphingomonas corticis 36D10-4-7 GCA 012035195.1	79.47
PB2P19	Parasphingomonas psychrolutea CGMCC 1.10106 GCA 014636175.1	79.43
PB2P19	Alterisphingomonas mali NBRC 15500 GCA 001598415.1	79.42
PB2P19	Sphingomonas dokdonensis DSM 21029 GCA 002197685.1	79.40
PB2P19	Alterisphingomonas pruni NBRC 15498 GCA 001598455.1	79.38
PB2P19	Novistakelama hankookensis KCTC 22579 GCA 022664465.1	79.38
PB2P19	Alteristakelama azotifigens NBRC 15497 GCA 002091475.1	79.34
PB2P19	Sphingomonas olei NM83 B4-11 GCA 004801655.1	79.34
PB2P19	Alteristakelama kyeonggiensis DSM 101806 GCA 014196745.1	79.33
PB2P19	Parastakelama yantingensis DSM 27244 GCA 014199325.1	79.33
PB2P19	Novistakelama panni DSM 15761 GCA 022664435.1	79.32
PB2P19	Alteristakelama koreensis JSS26 GCA 002797435.1	79.30
PB2P19	Parasphingomonas aracearum WZY 27 GCA 003345355.1	79.28
PB2P19	Alteristakelama pokkalii L3B27 GCA 003096275.1	79.28
PB2P19	Alteristakelama leidyi DSM 4733 GCA 011761945.1	79.26
PB2P19	Parastakelama baiyangensis L-1-4 w-11 GCA 005144715.1	79.25
PB2P19	Sphingomonas gellani S6-262 GCA 900110035.1	79.24
PB2P19	Alteristakelama psychrotolerans Cra20 GCA 002796605.1	79.23
PB2P19	Parastakelama spermidinifaciens 9NM-10 GCA 002351485.1	79.22
PB2P19	Sphingomonas caeni LB-2 GCA 026013415.1	79.19
PB2P19	Alteristakelama xinjiangensis DSM 26736 GCA 014199255.1	79.16
PB2P19	Sphingomonas lenta 1PNM-20 GCA 002288825.1	78.98

PB2P19	Alteristakelama soli NBRC 100801 GCA 001591025.1	78.97
PB2P19	Pseudostakelama guangdongensis CGMCC 1.12672 GCA 900199185.1	78.92
PB2P19	Alteristakelama suaedae XS-10 GCA 007833215.1	78.90
PB2P19	Alteristakelama canadensis FWC47 GCA 026013525.1	78.89
PB2P19	Alteriyabuuchia sanxanigenens NX02 GCA 000512205.2	78.87
PB2P19	Sphingomonas horti MAH-20 GCA 009753715.1	78.86
PB2P19	Alteristakelama turrisvirgatae MCT13 GCA 001721295.1	78.82
PB2P19	Sphingomonas mucosissima DSM 17494 GCA 002197665.1	78.80
PB2P19	Pararhizorhabdus prati CGMCC 1.15645 GCA 014643515.1	78.79
PB2P19	**LT1P40**	78.78
PB2P19	Solisphingomonas chungangi MAH-6 GCA 009763135.1	78.74
PB2P19	Sphingomonas naphthae KACC 18716 GCA 028607085.1	78.74
PB2P19	Parayabuuchia changbaiensis NBRC 104936 GCA 000974765.1	78.73
PB2P19	Yabuuchia cavernae K2R01-6 GCA 003590775.1	78.70
PB2P19	Parayabuuchia flavalba ZLT-5 GCA 004796535.1	78.65
PB2P19	Sphingomonas aerophila DSM 100044 GCA 014199305.1	78.65
PB2P19	Pararhizorhabdus montana W16RD GCA 001956315.1	78.62
PB2P19	Sphingomonas nostoxanthinifaciens AK-PDB1-5 GCA 019930585.1	78.60
PB2P19	Alterirhizorhabdus solaris R4DWN GCA 007785815.1	78.55
PB2P19	Yabuuchia colocasiae JCM 31229 GCA 019880585.1	78.54
PB2P19	Sphingomonas changnyeongensis C33 GCA 009913435.1	78.53
PB2P19	Rhizorhabdus crocodyli CCP-7 GCA 004005865.1	78.52
PB2P19	Pararhizorhabdus jatrophae S5-249 GCA 900113315.1	78.44
PB2P19	Solisphingomonas oligoaromativorans DSM 102246 GCA 011762195.1	78.40
PB2P19	Rhizorhabdus montanisoli ZX GCA 008274695.1	78.38
PB2P19	Solisphingomonas quercus XMGL2 GCA 018863195.1	78.32
PB2P19	Sphingomonas parva 17J27-24 GCA 004564275.1	78.30
PB2P19	Pseudosphingomonas astaxanthinifaciens DSM 22298 GCA 000711715.1	78.30
PB2P19	Sphingomonas ursincola KR-99 GCA 013607875.1	78.29
PB2P19	Neorhizorhabdus oleivorans FW-11 GCA 003050615.1	78.28
PB2P19	Pseudosphingomonas ginsengisoli KCTC 12630 GCA 003332855.1	78.28
PB2P19	Edaphosphingomonas haloaromaticamans P3 GCA 001853345.1	78.26
PB2P19	Edaphosphingomonas laterariae LNB2 GCA 900188165.1	78.24
PB2P19	Edaphosphingomonas fennica K101 GCA 003034225.1	78.19
PB2P19	Parayabuuchia jejuensis DSM 27651 GCA 011927695.1	78.13
PB2P19	Neorhizorhabdus vulcanisoli CECT 8804 GCA 011761305.1	78.13
PB2P19	Sphingomonas glaciei S8-45 GCA 023380025.1	78.12
PB2P19	Alterirhizorhabdus profundi LMO-1 GCA 009739515.1	78.10
PB2P19	Pseudosphingomonas rhizophila KACC 19189 GCA 014396585.1	78.10
PB2P19	Pseudosphingomonas mesophila SYSUP0001 GCA 003499275.1	78.09
PB2P19	Allosphingosinicella deserti GL-C-18 GCA 003012735.1	78.06
PB2P19	Pseudosphingomonas jaspsi DSM 18422 GCA 000585415.1	78.00
PB2P19	Flavisphingomonas formosensis CC-Nfb-2 GCA 009755815.1	77.98
PB2P19	Pseudosphingomonas ginkgonis HMF7854 GCA 003970925.1	77.94

PB2P19	Neorhizorhabdus crusticola MIMD3 GCA 003391115.1	77.94
PB2P19	Pseudosphingomonas kaistensis DSM 16846 GCA 011927725.1	77.92
PB2P19	Pseudosphingomonas lutea KCTC 23642 GCA 014396785.1	77.91
PB2P19	Sphingomonas bisphenolicum AO1 GCA 024349785.1	77.88
PB2P19	Pseudosphingomonas sinipercae HDW15C GCA 011302055.1	77.86
PB2P19	Pseudosphingomonas arenae SYSU D00720 GCA 016924655.1	77.77
PB2P19	Sphingomonas alba SE158 GCA 023516555.1	77.75
PB2P19	Pseudosphingomonas segetis YJ09 GCA 009720245.1	77.73
PB2P19	Solisphingomonas morindae NBD5 GCA 023822065.1	77.73
PB2P19	Sphingomonas telluris SM33 GCA 022568775.1	77.67
PB2P19	Sphingomonas brevis RB56-2 GCA 023516505.1	77.64
PB2P19	Pseudosphingomonas sabuli sand1-3 GCA 014352855.1	77.63
PB2P19	Sphingomonas xanthus AE3 GCA 007998985.1	77.49
PB2P19	Sphingomonas hankyongi SE220 GCA 023516575.1	77.38
PB2P19	Sphingomonas anseongensis RG327 GCA 023516495.1	77.37
PB2P19	Pseudosphingomonas piscis HDW15B GCA 011300455.1	77.35
PB4P5	Parasphingomonas hylomeconis CCTCC AB 2013304 GCA 025370105.1	86.71
PB4P5	Parasphingomonas qilianensis CGMCC 1.15349 GCA 039614825.1	85.65
PB4P5	**GB1N7**	82.55
PB4P5	Parasphingomonas aliaeris DH-S5 GCA 016743815.1	81.19
PB4P5	**RB3P16**	81.03
PB4P5	Parasphingomonas glacialis CGMCC 1.8957 GCA 014653575.1	80.99
PB4P5	Parasphingomonas alpina DSM 22537 GCA 014490665.1	80.88
PB4P5	Parasphingomonas echinoides ATCC 14820 GCA 000241465.1	80.41
PB4P5	**ZT3P38**	80.38
PB4P5	**RT2P30**	80.28
PB4P5	**PB2P19**	80.18
PB4P5	Parasphingomonas psychrolutea CGMCC 1.10106 GCA 014636175.1	80.12
PB4P5	Parasphingomonas panacis DCY99 GCA 001717955.1	80.01
PB4P5	Parasphingomonas populi 3 月 7 日 GCA 004208535.1	79.95
PB4P5	Sphingomonas ginsenosidivorax KHI67 GCA 007995065.1	79.94
PB4P5	Sphingomonas oligophenolica CGMCC 1.10181 GCA 039615115.1	79.88
PB4P5	Sphingomonas aurantiaca MA101b GCA 003050705.1	79.84
PB4P5	Sphingomonas taxi ATCC 55669 GCA 000764535.1	79.77
PB4P5	Sphingomonas aerolata NW12 GCA 003046295.1	79.77
PB4P5	Sphingomonas liriopis RP10 GCA 024211255.1	79.72
PB4P5	Sphingomonas albertensis DOAB 1063 GCA 014358075.1	79.52
PB4P5	**LB3N6**	79.50
PB4P5	**LB2R24**	79.44
PB4P5	Sphingomonas lycopersici MMSM20 GCA 026130605.1	79.40
PB4P5	Alterisphingomonas mali NBRC 15500 GCA 001598415.1	79.38
PB4P5	Sphingomonas faeni MA-olki GCA 003053745.1	79.37
PB4P5	**ZB1N12**	79.36
PB4P5	Alterisphingomonas pruni NBRC 15498 GCA 001598455.1	79.34

PB4P5	Alteristakelama naasensis DSM 100060 GCA 011762145.1	79.33
PB4P5	Parasphingomonas aracearum WZY 27 GCA 003345355.1	79.33
PB4P5	Alterisphingomonas asaccharolytica NBRC 15499 GCA 001598355.1	79.30
PB4P5	Sphingomonas tagetis MG17 GCA 024211275.1	79.27
PB4P5	Sphingomonas melonis DAPP-PG 224 GCA 000379045.1	79.26
PB4P5	Alteristakelama pituitosa NBRC 102491 GCA 001598435.1	79.25
PB4P5	Sphingomonas aquatilis DSM 15581 GCA 014196115.1	79.25
PB4P5	Sphingomonas insulae KCTC 12872 GCA 010450875.1	79.21
PB4P5	Sphingomonas metalli CGMCC 1.15330 GCA 014641735.1	79.20
PB4P5	Parastakelama japonica DSM 22753 GCA 011762085.1	79.20
PB4P5	Humisphingomonas gilva ZDH117 GCA 003515075.1	79.19
PB4P5	**PB2P12**	79.14
PB4P5	Alteristakelama gei ZFGT-11 GCA 004792685.1	79.14
PB4P5	Alteristakelama hengshuiensis WHSC-8 GCA 000935025.1	79.13
PB4P5	Alteristakelama trueperi DSM 7225 GCA 011927635.1	79.12
PB4P5	Sphingomonas rubra CGMCC 1.9113 GCA 900115745.1	79.12
PB4P5	Sphingomonas qomolangmaensis S5-59 GCA 024496245.1	79.11
PB4P5	Alteristakelama kyeonggiensis DSM 101806 GCA 014196745.1	79.09
PB4P5	Sphingomonas adhaesiva DSM 7418 GCA 002374855.1	79.08
PB4P5	Alteristakelama leidyi DSM 4733 GCA 011761945.1	79.07
PB4P5	Alteristakelama azotifigens NBRC 15497 GCA 002091475.1	79.06
PB4P5	Alterisphingomonas panacisoli HKS19 GCA 007859635.1	79.05
PB4P5	Sphingomonas donggukensis RMG20 GCA 023674425.1	79.03
PB4P5	Sphingomonas pseudosanguinis DSM 19512 GCA 014196255.1	79.02
PB4P5	Sphingomonas jeddahensis G39 GCA 001981525.1	78.99
PB4P5	Sphingomonas ginsenosidimutans KACC 14949 GCA 002374835.1	78.97
PB4P5	Pseudostakelama cannabina DM2-R-LB4 GCA 021391395.1	78.96
PB4P5	Sphingomonas caeni LB-2 GCA 026013415.1	78.96
PB4P5	Sphingomonas carotinifaciens DSM 27347 GCA 009789535.1	78.96
PB4P5	Alteristakelama soli NBRC 100801 GCA 001591025.1	78.94
PB4P5	Sphingomonas endophytica DSM 101535 GCA 014199415.1	78.93
PB4P5	Sphingomonas elodea ATCC 31461 GCA 000226955.2	78.92
PB4P5	Sphingomonas jinjuensis YC6723 GCA 014197105.1	78.92
PB4P5	Novistakelama desiccabilis DSM 16792 GCA 014196135.1	78.91
PB4P5	Alteristakelama psychrotolerans Cra20 GCA 002796605.1	78.90
PB4P5	Sphingomonas citri RRHST34 GCA 019429485.1	78.88
PB4P5	Sphingomonas parapaucimobilis NBRC 15100 GCA 000787715.1	78.84
PB4P5	Sphingomonas abaci DSM 15867 GCA 014199625.1	78.84
PB4P5	Alteristakelama suaedae XS-10 GCA 007833215.1	78.83
PB4P5	Sphingomonas yabuuchiae DSM 14562 GCA 014199595.1	78.83
PB4P5	Sphingomonas dokdonensis DSM 21029 GCA 002197685.1	78.82
PB4P5	**PB1R3**	78.82
PB4P5	Sphingomonas yunnanensis YIM 3 GCA 019898765.1	78.82
PB4P5	Sphingomonas phyllosphaerae FA2 GCA 000427645.1	78.80

PB4P5	Alteristakelama koreensis JSS26 GCA 002797435.1	78.79
PB4P5	Alteristakelama pokkalii L3B27 GCA 003096275.1	78.78
PB4P5	Parastakelama baiyangensis L-1-4 w-11 GCA 005144715.1	78.78
PB4P5	Sphingomonas beigongshangi REN5 GCA 016820445.1	78.77
PB4P5	Alterisphingomonas radiodurans S9-5 GCA 020866845.1	78.77
PB4P5	Sphingomonas sanguinis NBRC 13937 GCA 001591005.1	78.73
PB4P5	Sphingomonas folli RHCKR7 GCA 019429525.1	78.72
PB4P5	Alteristakelama canadensis FWC47 GCA 026013525.1	78.69
PB4P5	Sphingomonas zeae DSM 100049 GCA 014197135.1	78.66
PB4P5	Sphingomonas citricola RHCKR47 GCA 019429535.1	78.65
PB4P5	Sphingomonas hominis HHU CXW GCA 013328205.1	78.63
PB4P5	Alteristakelama turrisvirgatae MCT13 GCA 001721295.1	78.63
PB4P5	Sphingomonas paucimobilis NCTC11030 GCA 900457515.1	78.57
PB4P5	**LT1P40**	78.55
PB4P5	Sphingomonas olei NM83 B4-11 GCA 004801655.1	78.55
PB4P5	Alteristakelama xinjiangensis DSM 26736 GCA 014199255.1	78.54
PB4P5	Novistakelama panni DSM 15761 GCA 022664435.1	78.50
PB4P5	Parastakelama spermidinifaciens 9NM-10 GCA 002351485.1	78.47
PB4P5	Parastakelama yantingensis DSM 27244 GCA 014199325.1	78.46
PB4P5	Sphingomonas mucosissima DSM 17494 GCA 002197665.1	78.46
PB4P5	Sphingomonas palmae JS21-1 GCA 900109565.1	78.43
PB4P5	Novistakelama hankookensis KCTC 22579 GCA 022664465.1	78.42
PB4P5	Sphingomonas corticis 36D10-4-7 GCA 012035195.1	78.42
PB4P5	Alteriyabuuchia sanxanigenens NX02 GCA 000512205.2	78.39
PB4P5	Sphingomonas naphthae KACC 18716 GCA 028607085.1	78.39
PB4P5	Sphingomonas nostoxanthinifaciens AK-PDB1-5 GCA 019930585.1	78.34
PB4P5	Neorhizorhabdus vulcanisoli CECT 8804 GCA 011761305.1	78.32
PB4P5	Parayabuuchia changbaiensis NBRC 104936 GCA 000974765.1	78.27
PB4P5	Sphingomonas gellani S6-262 GCA 900110035.1	78.27
PB4P5	Edaphosphingomonas laterariae LNB2 GCA 900188165.1	78.26
PB4P5	Yabuuchia cavernae K2R01-6 GCA 003590775.1	78.25
PB4P5	Solisphingomonas chungangi MAH-6 GCA 009763135.1	78.24
PB4P5	Alterirhizorhabdus solaris R4DWN GCA 007785815.1	78.22
PB4P5	Pararhizorhabdus prati CGMCC 1.15645 GCA 014643515.1	78.21
PB4P5	Yabuuchia colocasiae JCM 31229 GCA 019880585.1	78.20
PB4P5	Edaphosphingomonas fennica K101 GCA 003034225.1	78.17
PB4P5	Sphingomonas changnyeongensis C33 GCA 009913435.1	78.16
PB4P5	Sphingomonas horti MAH-20 GCA 009753715.1	78.13
PB4P5	Edaphosphingomonas haloaromaticamans P3 GCA 001853345.1	78.09
PB4P5	Parayabuuchia flavalba ZLT-5 GCA 004796535.1	78.08
PB4P5	Sphingomonas ursincola KR-99 GCA 013607875.1	78.06
PB4P5	Rhizorhabdus crocodyli CCP-7 GCA 004005865.1	78.06
PB4P5	Neorhizorhabdus oleivorans FW-11 GCA 003050615.1	78.05
PB4P5	Pseudostakelama guangdongensis CGMCC 1.12672 GCA 900199185.1	78.05

PB4P5	Pararhizorhabdus jatrophae S5-249 GCA 900113315.1	78.05
PB4P5	Alterirhizorhabdus profundi LMO-1 GCA 009739515.1	78.04
PB4P5	Neorhizorhabdus crusticola MIMD3 GCA 003391115.1	78.03
PB4P5	Solisphingomonas quercus XMGL2 GCA 018863195.1	78.00
PB4P5	Rhizorhabdus montanisoli ZX GCA 008274695.1	77.99
PB4P5	Solisphingomonas oligoaromativorans DSM 102246 GCA 011762195.1	77.99
PB4P5	Pararhizorhabdus montana W16RD GCA 001956315.1	77.98
PB4P5	Sphingomonas lenta 1PNM-20 GCA 002288825.1	77.93
PB4P5	Sphingomonas bisphenolicum AO1 GCA 024349785.1	77.89
PB4P5	Solisphingomonas morindae NBD5 GCA 023822065.1	77.87
PB4P5	Pseudosphingomonas astaxanthinifaciens DSM 22298 GCA 000711715.1	77.83
PB4P5	Flavisphingomonas formosensis CC-Nfb-2 GCA 009755815.1	77.78
PB4P5	Sphingomonas parva 17J27-24 GCA 004564275.1	77.76
PB4P5	Sphingomonas aerophila DSM 100044 GCA 014199305.1	77.75
PB4P5	Pseudosphingomonas sinipercae HDW15C GCA 011302055.1	77.67
PB4P5	Pseudosphingomonas kaistensis DSM 16846 GCA 011927725.1	77.67
PB4P5	Parayabuuchia jejuensis DSM 27651 GCA 011927695.1	77.66
PB4P5	Pseudosphingomonas mesophila SYSUP0001 GCA 003499275.1	77.65
PB4P5	Pseudosphingomonas jaspsi DSM 18422 GCA 000585415.1	77.64
PB4P5	Pseudosphingomonas sabuli sand1-3 GCA 014352855.1	77.56
PB4P5	Sphingomonas alba SE158 GCA 023516555.1	77.55
PB4P5	Pseudosphingomonas lutea KCTC 23642 GCA 014396785.1	77.54
PB4P5	Pseudosphingomonas rhizophila KACC 19189 GCA 014396585.1	77.53
PB4P5	Sphingomonas glaciei S8-45 GCA 023380025.1	77.51
PB4P5	Allosphingosinicella deserti GL-C-18 GCA 003012735.1	77.42
PB4P5	Pseudosphingomonas ginsengisoli KCTC 12630 GCA 003332855.1	77.40
PB4P5	Pseudosphingomonas segetis YJ09 GCA 009720245.1	77.38
PB4P5	Sphingomonas cremea G124 GCA 021502585.1	77.34
PB4P5	Pseudosphingomonas ginkgonis HMF7854 GCA 003970925.1	77.34
PB4P5	Sphingomonas xanthus AE3 GCA 007998985.1	77.30
PB4P5	Sphingomonas brevis RB56-2 GCA 023516505.1	77.25
PB4P5	Sphingomonas anseongensis RG327 GCA 023516495.1	77.20
PB4P5	Pseudosphingomonas piscis HDW15B GCA 011300455.1	77.06
PB1R3	Sphingomonas parapaucimobilis NBRC 15100 GCA 000787715.1	88.49
PB1R3	Sphingomonas yabuuchiae DSM 14562 GCA 014199595.1	88.21
PB1R3	Sphingomonas sanguinis NBRC 13937 GCA 001591005.1	88.14
PB1R3	Sphingomonas pseudosanguinis DSM 19512 GCA 014196255.1	87.54
PB1R3	Sphingomonas zeae DSM 100049 GCA 014197135.1	87.43
PB1R3	Sphingomonas paucimobilis NCTC11030 GCA 900457515.1	86.65
PB1R3	Sphingomonas carotinifaciens DSM 27347 GCA 009789535.1	81.95
PB1R3	Sphingomonas abaci DSM 15867 GCA 014199625.1	81.29
PB1R3	Sphingomonas metalli CGMCC 1.15330 GCA 014641735.1	81.10
PB1R3	Sphingomonas melonis DAPP-PG 224 GCA 000379045.1	80.80
PB1R3	Sphingomonas liriopis RP10 GCA 024211255.1	80.79

PB1R3	Sphingomonas aquatilis DSM 15581 GCA 014196115.1	80.60
PB1R3	Sphingomonas jinjuensis YC6723 GCA 014197105.1	80.50
PB1R3	Sphingomonas taxi ATCC 55669 GCA 000764535.1	80.41
PB1R3	Sphingomonas adhaesiva DSM 7418 GCA 002374855.1	80.34
PB1R3	Sphingomonas rubra CGMCC 1.9113 GCA 900115745.1	80.27
PB1R3	Sphingomonas ginsenosidivorax KHI67 GCA 007995065.1	80.27
PB1R3	Sphingomonas beigongshangi REN5 GCA 016820445.1	80.26
PB1R3	Sphingomonas insulae KCTC 12872 GCA 010450875.1	80.23
PB1R3	Sphingomonas endophytica DSM 101535 GCA 014199415.1	80.19
PB1R3	Sphingomonas ginsenosidimutans KACC 14949 GCA 002374835.1	80.19
PB1R3	Sphingomonas aerolata NW12 GCA 003046295.1	80.14
PB1R3	**PB2P19**	80.09
PB1R3	Novistakelama hankookensis KCTC 22579 GCA 022664465.1	80.02
PB1R3	Sphingomonas phyllosphaerae FA2 GCA 000427645.1	79.95
PB1R3	Sphingomonas aurantiaca MA101b GCA 003050705.1	79.94
PB1R3	Sphingomonas gellani S6-262 GCA 900110035.1	79.85
PB1R3	Novistakelama panni DSM 15761 GCA 022664435.1	79.84
PB1R3	Sphingomonas palmae JS21-1 GCA 900109565.1	79.79
PB1R3	Sphingomonas citricola RHCKR47 GCA 019429535.1	79.76
PB1R3	Sphingomonas folli RHCKR7 GCA 019429525.1	79.73
PB1R3	Sphingomonas citri RRHST34 GCA 019429485.1	79.67
PB1R3	Parasphingomonas aracearum WZY 27 GCA 003345355.1	79.62
PB1R3	Sphingomonas corticis 36D10-4-7 GCA 012035195.1	79.60
PB1R3	Sphingomonas dokdonensis DSM 21029 GCA 002197685.1	79.56
PB1R3	Novistakelama desiccabilis DSM 16792 GCA 014196135.1	79.56
PB1R3	Parastakelama yantingensis DSM 27244 GCA 014199325.1	79.54
PB1R3	Alteristakelama pituitosa NBRC 102491 GCA 001598435.1	79.52
PB1R3	Sphingomonas elodea ATCC 31461 GCA 000226955.2	79.49
PB1R3	Sphingomonas albertensis DOAB 1063 GCA 014358075.1	79.48
PB1R3	Pseudostakelama cannabina DM2-R-LB4 GCA 021391395.1	79.48
PB1R3	**RT2P30**	79.46
PB1R3	**ZB1N12**	79.45
PB1R3	Sphingomonas yunnanensis YIM 3 GCA 019898765.1	79.45
PB1R3	Alteristakelama pokkalii L3B27 GCA 003096275.1	79.45
PB1R3	Sphingomonas hominis HHU CXW GCA 013328205.1	79.45
PB1R3	Sphingomonas donggukensis RMG20 GCA 023674425.1	79.42
PB1R3	Sphingomonas olei NM83 B4-11 GCA 004801655.1	79.42
PB1R3	Alteristakelama kyeonggiensis DSM 101806 GCA 014196745.1	79.40
PB1R3	Alteristakelama trueperi DSM 7225 GCA 011927635.1	79.40
PB1R3	Alteristakelama azotifigens NBRC 15497 GCA 002091475.1	79.37
PB1R3	Alteristakelama leidyi DSM 4733 GCA 011761945.1	79.34
PB1R3	Parasphingomonas hylomeconis CCTCC AB 2013304 GCA 025370105.1	79.34
PB1R3	Parastakelama spermidinifaciens 9NM-10 GCA 002351485.1	79.34
PB1R3	Sphingomonas qomolangmaensis S5-59 GCA 024496245.1	79.34

PB1R3	Humisphingomonas gilva ZDH117 GCA 003515075.1	79.32
PB1R3	**LB3N6**	79.32
PB1R3	**PB2P12**	79.32
PB1R3	Sphingomonas faeni MA-olki GCA 003053745.1	79.32
PB1R3	**LB2R24**	79.30
PB1R3	Alterisphingomonas mali NBRC 15500 GCA 001598415.1	79.28
PB1R3	Parasphingomonas echinoides ATCC 14820 GCA 000241465.1	79.24
PB1R3	Alteristakelama hengshuiensis WHSC-8 GCA 000935025.1	79.23
PB1R3	Sphingomonas jeddahensis G39 GCA 001981525.1	79.23
PB1R3	Alterisphingomonas pruni NBRC 15498 GCA 001598455.1	79.19
PB1R3	**RB3P16**	79.18
PB1R3	Sphingomonas lycopersici MMSM20 GCA 026130605.1	79.18
PB1R3	**ZT3P38**	79.17
PB1R3	Alterisphingomonas asaccharolytica NBRC 15499 GCA 001598355.1	79.16
PB1R3	Parasphingomonas populi 3 月 7 日 GCA 004208535.1	79.11
PB1R3	**GB1N7**	79.11
PB1R3	Alteristakelama koreensis JSS26 GCA 002797435.1	79.10
PB1R3	Alteristakelama suaedae XS-10 GCA 007833215.1	79.09
PB1R3	Alteristakelama xinjiangensis DSM 26736 GCA 014199255.1	79.09
PB1R3	Alterisphingomonas panacisoli HKS19 GCA 007859635.1	79.04
PB1R3	Alteristakelama canadensis FWC47 GCA 026013525.1	79.03
PB1R3	Sphingomonas lenta 1PNM-20 GCA 002288825.1	79.03
PB1R3	Sphingomonas tagetis MG17 GCA 024211275.1	79.03
PB1R3	Parastakelama japonica DSM 22753 GCA 011762085.1	79.02
PB1R3	Parasphingomonas panacis DCY99 GCA 001717955.1	79.02
PB1R3	Sphingomonas caeni LB-2 GCA 026013415.1	79.02
PB1R3	Parastakelama baiyangensis L-1-4 w-11 GCA 005144715.1	79.02
PB1R3	Alteristakelama turrisvirgatae MCT13 GCA 001721295.1	79.01
PB1R3	Parasphingomonas aliaeris DH-S5 GCA 016743815.1	79.01
PB1R3	Sphingomonas mucosissima DSM 17494 GCA 002197665.1	79.01
PB1R3	Parasphingomonas alpina DSM 22537 GCA 014490665.1	78.98
PB1R3	Alteristakelama naasensis DSM 100060 GCA 011762145.1	78.98
PB1R3	Sphingomonas oligophenolica CGMCC 1.10181 GCA 039615115.1	78.97
PB1R3	Parasphingomonas glacialis CGMCC 1.8957 GCA 014653575.1	78.95
PB1R3	Pseudostakelama guangdongensis CGMCC 1.12672 GCA 900199185.1	78.94
PB1R3	Parasphingomonas qilianensis CGMCC 1.15349 GCA 039614825.1	78.91
PB1R3	Sphingomonas changnyeongensis C33 GCA 009913435.1	78.89
PB1R3	Pararhizorhabdus jatrophae S5-249 GCA 900113315.1	78.88
PB1R3	**PB4P5**	78.87
PB1R3	Sphingomonas aerophila DSM 100044 GCA 014199305.1	78.86
PB1R3	Sphingomonas naphthae KACC 18716 GCA 028607085.1	78.81
PB1R3	Yabuuchia colocasiae JCM 31229 GCA 019880585.1	78.81
PB1R3	Alterisphingomonas radiodurans S9-5 GCA 020866845.1	78.80
PB1R3	Solisphingomonas oligoaromativorans DSM 102246 GCA 011762195.1	78.78

PB1R3	Sphingomonas horti MAH-20 GCA 009753715.1	78.78
PB1R3	Rhizorhabdus crocodyli CCP-7 GCA 004005865.1	78.76
PB1R3	Rhizorhabdus montanisoli ZX GCA 008274695.1	78.72
PB1R3	Alteristakelama gei ZFGT-11 GCA 004792685.1	78.69
PB1R3	Parayabuuchia changbaiensis NBRC 104936 GCA 000974765.1	78.69
PB1R3	**LT1P40**	78.68
PB1R3	Alteriyabuuchia sanxanigenens NX02 GCA 000512205.2	78.65
PB1R3	Sphingomonas nostoxanthinifaciens AK-PDB1-5 GCA 019930585.1	78.64
PB1R3	Alterirhizorhabdus solaris R4DWN GCA 007785815.1	78.61
PB1R3	Yabuuchia cavernae K2R01-6 GCA 003590775.1	78.57
PB1R3	Alteristakelama soli NBRC 100801 GCA 001591025.1	78.55
PB1R3	Edaphosphingomonas haloaromaticamans P3 GCA 001853345.1	78.54
PB1R3	Alteristakelama psychrotolerans Cra20 GCA 002796605.1	78.54
PB1R3	Parayabuuchia flavalba ZLT-5 GCA 004796535.1	78.53
PB1R3	Edaphosphingomonas fennica K101 GCA 003034225.1	78.53
PB1R3	Edaphosphingomonas laterariae LNB2 GCA 900188165.1	78.46
PB1R3	Neorhizorhabdus oleivorans FW-11 GCA 003050615.1	78.44
PB1R3	Solisphingomonas chungangi MAH-6 GCA 009763135.1	78.39
PB1R3	Parayabuuchia jejuensis DSM 27651 GCA 011927695.1	78.38
PB1R3	Solisphingomonas quercus XMGL2 GCA 018863195.1	78.37
PB1R3	Parasphingomonas psychrolutea CGMCC 1.10106 GCA 014636175.1	78.32
PB1R3	Sphingomonas ursincola KR-99 GCA 013607875.1	78.29
PB1R3	Sphingomonas bisphenolicum AO1 GCA 024349785.1	78.25
PB1R3	Pararhizorhabdus prati CGMCC 1.15645 GCA 014643515.1	78.25
PB1R3	Flavisphingomonas formosensis CC-Nfb-2 GCA 009755815.1	78.22
PB1R3	Pararhizorhabdus montana W16RD GCA 001956315.1	78.16
PB1R3	Pseudosphingomonas astaxanthinifaciens DSM 22298 GCA 000711715.1	78.12
PB1R3	Alterirhizorhabdus profundi LMO-1 GCA 009739515.1	78.11
PB1R3	Solisphingomonas morindae NBD5 GCA 023822065.1	78.10
PB1R3	Sphingomonas caseinilyticus NSE70-1 GCA 023516455.1	78.09
PB1R3	Pseudosphingomonas ginkgonis HMF7854 GCA 003970925.1	78.06
PB1R3	Neorhizorhabdus crusticola MIMD3 GCA 003391115.1	78.06
PB1R3	Pseudosphingomonas jaspsi DSM 18422 GCA 000585415.1	78.05
PB1R3	Pseudosphingomonas rhizophila KACC 19189 GCA 014396585.1	78.05
PB1R3	Neorhizorhabdus vulcanisoli CECT 8804 GCA 011761305.1	78.04
PB1R3	Pseudosphingomonas ginsengisoli KCTC 12630 GCA 003332855.1	77.93
PB1R3	Sphingomonas alba SE158 GCA 023516555.1	77.90
PB1R3	Pseudosphingomonas kaistensis DSM 16846 GCA 011927725.1	77.90
PB1R3	Sphingomonas parva 17J27-24 GCA 004564275.1	77.88
PB1R3	Pseudosphingomonas mesophila SYSUP0001 GCA 003499275.1	77.86
PB1R3	Pseudosphingomonas sinipercae HDW15C GCA 011302055.1	77.80
PB1R3	Pseudosphingomonas sabuli sand1-3 GCA 014352855.1	77.78
PB1R3	Allosphingosinicella deserti GL-C-18 GCA 003012735.1	77.78
PB1R3	Sphingomonas glaciei S8-45 GCA 023380025.1	77.71

PB1R3	Sphingomonas brevis RB56-2 GCA 023516505.1	77.70
PB1R3	Pseudosphingomonas arenae SYSU D00720 GCA 016924655.1	77.67
PB1R3	Pseudosphingomonas piscis HDW15B GCA 011300455.1	77.67
PB1R3	Pseudosphingomonas lutea KCTC 23642 GCA 014396785.1	77.58
PB1R3	Sphingomonas anseongensis RG327 GCA 023516495.1	77.52
PB1R3	Pseudosphingomonas segetis YJ09 GCA 009720245.1	77.42
PB1R3	Pseudosphingomonas edaphi DAC4 GCA 003583725.1	77.41
PB1R3	Sphingomonas xanthus AE3 GCA 007998985.1	77.40

Table S3. Closest reference strains in the NCBI database based on ANI values with the genomes of 12 novel species.

Strains	Reference strain	ANI (%)
LT1P40	Sphingomonas tagetis MG17 GCA_024211275.1	82.47
**LB2R24**	Sphingomonas sp. PP-F2F-A104-K0414 GCA_004340945.1	**95.54**
**LB2R24**	Sphingomonas sp. PFN1.45 GCA_040407455.1	**95.48**
**LB2R24**	Sphingomonas sp. LR59 GCA_036855945.1	**95.46**
**LB2R24**	Sphingomonas sp. PP-CC-3A-396 GCA_004343105.1	**95.29**
**LB2R24**	Sphingomonas sp. LR55 GCA_036856015.1	**95.21**
**LB3N6**	Sphingomonas sp. ERG1.4 GCA_023195535.1	**97.81**
**LB3N6**	Sphingomonas sp. ERG1.7 GCA_040407395.1	**97.70**
RB3P16	Sphingomonas sp. UYEF23 GCA_040546165.1	88.40
RT2P30	Sphingomonas sp. AR OL41 GCA_029911635.1	93.13
ZT3P38	Sphingomonas sp. GCA_041661825.1	84.92
ZB1N12	Sphingomonas faeni W4I17 GCA_030817315.1	94.61
GB1N7	Sphingomonas sp. Leaf357 GCA_001423845.1	89.67
PB2P12	Sphingomonas sp. Leaf205 GCA_920984725.1	89.44
PB2P19	Sphingomonas ginsenosidivorax KHI67 GCA_007995065.1	87.17
PB4P5	Sphingomonas hylomeconis CCTCC AB 2013304 GCA_025370105.1	86.71
**PB1R3**	Sphingomonas sp. CFBP8993 GCA_034044895.1	**97.45**
**PB1R3**	Sphingomonas sp. I4 GCA_037861125.1	**97.24**

Table S5. Phenotypic characteristics of the 12 novel strains. +, Positive; -, negative; ND, not detected.

	LT1P40^T	LB2R24^T	LB3N6^T	RB3P16^T	RT2P30^T	ZT3P38^T	ZB1N12^T	GB1N7^T	PB2P12^T	PB2P19^T	PB4P5^T	PB1R3^T
**Growth temperature range (℃)**	0-30	0-25	0-25	0-30	0-35	0-35	0-25	0-25	0-25	0-25	0-25	0-37
**pH range for growth**	4-11	4-9	4-9	4-9	5-8	4-11	5-10	4-9	4-8	5-8	5-9	4-10
**NaCl range for growth (w/v, %)**	0-1.0	0-1.5	0-3.0	0-1.5	0-0.05	0-1.0	0-1.5	0-1.0	0-1.5	0-1.0	0-0.5	0-1.5
**Flagellum**	+	+	+	+	+	+	+	+	+	+	+	+
**Reduce nitrate to nitrite**	-	-	-	-	-	-	-	-	-	-	-	-
**Indole production**	-	-	-	-	-	-	-	-	-	-	-	-
**H_2S production**	-	-	-	-	-	-	-	-	-	-	-	-
**Citrate utilization**	-	+	-	-	-	-	-	-	-	-	-	+
**Voges-Proskauer test**	-	+	+	+	-	+	+	+	+	+	-	+
**Hydrolysis of Tween 80**	-	-	-	-	-	-	-	-	-	-	-	-
**Hydrolysis of starch**	+	-	-	-	-	-	-	-	-	-	-	-
**Hydrolysis of casein**	-	-	-	-	-	-	-	+	-	-	-	-
**Hydrolysis of gelatin**	-	-	+	-	-	-	+	+	-	-	-	-
**Hydrolysis of esculin**	+	+	+	+	+	+	+	+	+	+	+	+
**Enzymatic activities:**												
catalase	+	+	+	+	+	+	+	+	+	+	+	+
oxidase	+	+	+	+	+	+	+	+	+	+	+	+
alkaline phosphatase	+	+	+	+	+	+	+	+	+	+	+	+
esterase(C4)	+	+	+	+	+	+	-	+	+	+	+	+
esterase lipase(C8)	+	+	+	+	+	+	-	+	+	+	+	+
lipase(C14)	+	+	+	+	+	-	-	-	+	+	+	-
leucine arylamidase	+	+	+	+	+	+	+	+	+	+	+	+
valine arylamidase	+	+	+	+	+	+	+	+	+	+	+	+
cystine arylamidase	+	+	+	+	+	+	+	+	+	+	+	+
trypsin	+	+	+	-	-	-	+	+	+	-	+	+
α-chymotrypsin	+	-	-	+	+	-	-	+	-	+	+	+
acid phosphatase	+	+	+	+	+	+	+	+	+	+	+	+
naphthol-AS-BI-phosphohydrolase	+	+	+	+	+	+	+	+	+	+	+	+
α-galactosidase	-	-	-	-	+	-	+	-	+	-	+	+
β-glucuronidase	-	-	-	+	+	-	-	-	-	-	-	+
α-glucosidase	+	+	+	+	+	+	+	+	+	+	-	+
β-glucosidase	+	+	+	+	+	+	+	-	+	+	+	+
N-acetyl-β-glucosaminidase	+	-	+	-	-	+	+	+	-	-	-	+
α-fucosidase	-	-	+	-	-	-	-	-	-	-	-	-
tryptophane deaminase	-	-	-	+	-	-	-	-	-	-	-	-
β-galactosidase	+	+	+	+	+	+	+	+	+	+	+	+
**Acid produced from:**												
D-glucose	-	+	-	+	+	-	-	-	+	-	-	+
L-rhamnose	-	-	-	+	-	+	-	-	-	+	-	-
D-sucrose	-	+	-	+	+	-	-	-	+	-	-	+
D-melibiose	-	-	-	-	-	-	-	-	+	-	-	+
amygdalin	-	-	-	+	-	-	-	-	+	-	-	+
L-arabinose	-	+	+	+	+	-	+	-	+	-	-	+

Utilization of carbon source：

dextrin	+	+	+	+	ND	-	+	+	-	+	-	+
D-maltose	-	+	+	+	+	-	+	+	+	+	-	+
D-trehalose	-	+	+	-	-	+	+	+	+	+	-	+
D-cellobiose	+	+	+	+	+	+	+	-	+	+	-	+
gentiobiose	+	+	+	+	+	-	+	-	+	+	+	+
sucrose	-	+	+	+	ND	+	+	+	+	-	+	+
D-turanose	-	+	+	+	+	-	+	-	-	+	+	+
stachyose	-	+	+	-	ND	-	-	-	-	-	+	+
D-raffinose	-	+	+	-	-	-	+	-	-	-	-	+
D-lactose	-	+	+	-	+	-	+	-	-	-	+	+
D-melibiose	-	+	+	-	-	-	+	-	-	+	+	+
β-methyl-D-glucoside	-	+	+	-	ND	-	+	-	-	-	-	+
D-salicin	-	+	+	-	ND	-	+	-	-	+	+	+
N-acetyl-D-glucosamine	+	+	+	+	ND	+	+	+	+	-	+	+
N-acetyl-β-D-mannosamine	-	+	-	-	ND	-	-	-	-	-	-	+
N-acetyl-D-galactosamine	-	-	-	-	ND	+	-	-	-	-	-	-
N-acetyl neuraminic acid	-	-	-	-	ND	-	-	-	-	-	-	-
α-D-glucose	-	+	+	+	+	+	+	+	+	+	-	+
D-mannose	-	+	+	+	-	+	+	+	+	+	-	+
D-fructose	+	+	+	+	-	-	+	+	+	+	-	+
D-galactose	-	+	+	+	+	-	+	+	+	+	+	+
3-methyl glucose	-	-	-	-	ND	-	-	-	-	-	-	-
D-fucose	+	-	-	+	-	+	+	+	+	-	+	+
L-fucose	+	-	+	-	-	-	+	+	+	-	+	+
L-rhamnose	+	-	-	+	-	+	+	-	-	+	-	-
inosine	-	-	-	-	ND	-	-	-	-	-	-	-
D-sorbitol	-	+	-	-	-	-	-	-	-	-	-	-
D-mannitol	-	-	-	-	-	-	-	-	-	-	-	-
D-arabitol	-	-	-	-	-	-	-	-	-	-	-	-
myo-inositol	-	-	-	-	ND	-	-	-	-	-	-	-
glycerol	-	-	-	-	-	-	-	+	-	-	-	-
D-glucose-6-PO_4	-	+	-	-	ND	-	-	-	-	-	-	+
D-fructose-6-PO_4	+	+	+	+	ND	+	+	+	+	+	+	+
D-aspartic acid	-	-	-	-	ND	-	-	-	-	-	-	-
D-serine	-	-	-	-	ND	-	-	-	-	-	-	-
gelatin	-	-	-	+	ND	-	+	+	+	-	-	+
glycyl-L-proline	+	+	+	+	ND	+	+	+	+	+	-	+
L-alanine	+	+	-	+	ND	-	+	+	+	-	-	+
L-arginine	-	-	-	-	ND	-	-	-	-	-	-	-
L-aspartic acid	+	+	-	+	ND	-	+	+	+	-	-	-
L-glutamic acid	-	+	+	+	ND	+	+	+	+	+	-	+
L-histidine	+	-	+	+	ND	+	+	+	-	+	-	+
L-pyroglutamic acid	-	-	-	-	ND	-	-	-	-	-	-	-
L-serine	+	+	-	-	ND	-	-	-	-	-	-	+
pectin	+	+	+	+	ND	+	+	+	+	-	-	+

D-galacturonic acid	+	+	+	+	ND	+	+	-	+	-	+	+
L-galactonic acid lactone	+	+	+	-	ND	+	+	-	+	-	+	-
D-gluconic acid	-	+	+	-	ND	+	+	-	+	-	-	-
D-glucuronic acid	+	-	+	+	ND	+	+	-	+	+	-	+
glucuronamide	+	+	+	+	ND	+	+	+	+	+	+	+
mucic acid	-	-	-	-	ND	-	-	-	-	-	-	-
quinic acid	-	-	-	+	ND	+	-	+	-	-	-	-
D-saccharic acid	-	-	-	-	ND	-	-	-	-	-	-	-
p-hydroxy- phenylacetic acid	-	-	-	-	ND	-	-	-	-	-	-	-
methyl pyruvate	-	-	+	-	ND	-	+	+	+	+	-	+
D-lactic acid methyl ester	-	-	-	-	ND	-	-	-	-	-	-	-
L-lactic acid	-	-	-	-	ND	+	-	-	-	-	-	+
α-keto-glutaric acid	-	+	+	-	ND	-	+	+	+	+	+	+
D-malic acid	-	-	-	-	ND	-	-	-	-	-	-	-
L-malic acid	+	+	+	+	ND	+	+	+	+	+	+	+
bromo-succinic acid	+	+	+	+	ND	+	+	+	+	+	-	+
Tween 40	+	+	+	+	ND	+	+	+	+	+	-	+
γ-amino butyric acid	-	-	-	-	ND	-	-	-	-	-	-	-
α-hydroxy butyric acid	-	-	-	-	ND	-	-	-	-	-	-	+
β-hydroxy-D,L-butyric acid	+	+	-	+	ND	+	+	+	+	-	+	+
α-keto-butyric acid	-	+	-	+	ND	-	-	-	-	-	-	+
acetoacetic acid	+	-	-	+	ND	-	-	-	-	+	-	+
propionic acid	-	+	-	-	ND	-	+	-	-	-	-	+
acetic acid	+	+	+	-	ND	+	+	-	+	+	-	+
formic acid	-	-	-	-	ND	+	+	-	-	-	-	-
potassium gluconate	ND	ND	ND	ND	+	ND	ND	ND	ND	ND	ND	ND
L-arabinose	ND	ND	ND	ND	+	ND	ND	ND	ND	ND	ND	ND
D-xylose	ND	ND	ND	ND	+	ND	ND	ND	ND	ND	ND	ND
D-saccharose	ND	ND	ND	ND	+	ND	ND	ND	ND	ND	ND	ND
